# The GenomeAsia 100K Project enables genetic discoveries across Asia

**DOI:** 10.1038/s41586-019-1793-z

**Published:** 2019-12-04

**Authors:** GenomeAsia100K Consortium, Jeffrey D. Wall, Eric W. Stawiski, Aakrosh Ratan, Hie Lim Kim, Changhoon Kim, Ravi Gupta, Kushal Suryamohan, Elena S. Gusareva, Rikky Wenang Purbojati, Tushar Bhangale, Vadim Stepanov, Vladimir Kharkov, Markus S. Schröder, Vedam Ramprasad, Jennifer Tom, Steffen Durinck, Qixin Bei, Jiani Li, Joseph Guillory, Sameer Phalke, Analabha Basu, Jeremy Stinson, Sandhya Nair, Sivasankar Malaichamy, Nidhan K. Biswas, John C. Chambers, Keith C. Cheng, Joyner T. George, Seik Soon Khor, Jong-Il Kim, Belong Cho, Ramesh Menon, Thiramsetti Sattibabu, Akshi Bassi, Manjari Deshmukh, Anjali Verma, Vivek Gopalan, Jong-Yeon Shin, Mahesh Pratapneni, Sam Santhosh, Katsushi Tokunaga, Badrul M. Md-Zain, Kok Gan Chan, Madasamy Parani, Purushothaman Natarajan, Michael Hauser, R. Rand Allingham, Cecilia Santiago-Turla, Arkasubhra Ghosh, Santosh Gopi Krishna Gadde, Christian Fuchsberger, Lukas Forer, Sebastian Schoenherr, Herawati Sudoyo, J. Stephen Lansing, Jonathan Friedlaender, George Koki, Murray P. Cox, Michael Hammer, Tatiana Karafet, Khai C. Ang, Syed Q. Mehdi, Venkatesan Radha, Viswanathan Mohan, Partha P. Majumder, Somasekar Seshagiri, Jeong-Sun Seo, Stephan C. Schuster, Andrew S. Peterson

**Affiliations:** 1 Institute for Human Genetics, University of California, San Francisco, San Francisco, CA USA; 2 Department of Molecular Biology, Genentech, South San Francisco, CA USA; 3 Department of Bioinformatics and Computational Biology, Genentech, South San Francisco, CA USA; 4 Center for Public Health Genomics, University of Virginia, Charlottesville, VA USA; 5 The Asian School of the Environment, Nanyang Technological University, Singapore, Singapore; 6 Singapore Centre for Environmental Life Sciences Engineering, Nanyang Technological University, Singapore, Singapore; 7 Bioinformatics Institute, Macrogen, Seoul, South Korea; 8 Precision Medicine Center, Seoul National University Bundang Hospital, Gyeonggi-do, South Korea; 9 MedGenome Labs, Bengaluru, India; 10 Department of Human Genetics, Genentech, South San Francisco, CA USA; 11 Institute of Medical Genetics, Tomsk National Medical Research Center, Tomsk, Russian Federation; 12 Russian Academy of Sciences, Tomsk, Russian Federation; 13 Tomsk State University, Tomsk, Russian Federation; 14 National Institute of BioMedical Genomics, Netaji Subhas Sanatorium, Kalyani, India; 15 Lee Kong Chian School of Medicine, Nanyang Technological University, Singapore, Singapore; 16 Department of Pathology and Jake Gittlen Laboratories for Cancer Research, Penn State College of Medicine, Hershey, PA USA; 17 Department of Human Genetics, University of Tokyo, Tokyo, Japan; 18 Department of Biomedical Sciences, Seoul National University Graduate School, Seoul, South Korea; 19 Genomic Medicine Institute (GMI), Medical Research Center, Seoul National University, Seoul, South Korea; 20 Department of Family Medicine, Seoul National University Hospital, Seoul, South Korea; 21 Precision Medicine Institute, Macrogen, Gyeonggi-do, South Korea; 22 Emerge Ventures, Singapore, Singapore; 23 Genome Medical Science Project, Toyama, Japan; 24 National Center Biobank Network (NCBN), National Center for Global Health and Medicine (NCGM), University of Tokyo, Tokyo, Japan; 25 School of Environment and Natural Resource Science, Faculty of Science and Technology, Universiti Kebangsaan Malaysia, Bangi, Malaysia; 26 Division of Genetics and Molecular Biology, Institute of Biological Sciences, Faculty of Science, University of Malaya, Kuala Lumpur, Malaysia; 27 Department of Genetic Engineering, SRM Institute of Science and Technology, Kattankulathur, India; 28 Department of Opthalmology, Duke University Medical Center, Durham, NC USA; 29 Department of Medicine, Duke University Medical Center, Durham, NC USA; 30 GROW Research Laboratory, Narayana Nethralaya Foundation, Bengaluru, India; 31 Department of Biostatistics, University of Michigan, Ann Arbor, MI USA; 32 Institute for Biomedicine, Eurac Research, Bolzano, Italy; 33 Institute of Genetic Epidemiology, Department of Genetics and Pharmacology, Medical University of Innsbruck, Innsbruck, Austria; 34 Genome Diversity and Diseases Laboratory, Eijkman Institute for Molecular Biology, Jakarta, Indonesia; 35 Complexity Institute, Nanyang Technological University, Singapore, Singapore; 36 Anthropology Department, Temple University, Philadelphia, PA USA; 37 Papua New Guinea Institute for Medical Research, Goroka, Papua New Guinea; 38 School of Fundamental Sciences, Massey University, Palmerston North, New Zealand; 39 Division of Biotechnology, University of Arizona, Tucson, AZ USA; 40 Center for Human Genetics, Sindh Institute of Urology and Transplantation, Karachi, Pakistan; 41 Madras Diabetes Research Foundation, Chennai, India; 42 Dr. Mohan’s Diabetes Specialities Centre, Chennai, India; 43 Human Genetics Unit, Indian Statistical Institute, Kolkata, India; 44 Present Address: Seven Rivers Genomic Medicines, A division of MedGenome, Foster City, CA USA; 45 Present Address: SciGenom Research Foundation, Chennai, Tamil Nadu India

**Keywords:** Genetic variation, Genetic variation

## Abstract

The underrepresentation of non-Europeans in human genetic studies so far has limited the diversity of individuals in genomic datasets and led to reduced medical relevance for a large proportion of the world’s population. Population-specific reference genome datasets as well as genome-wide association studies in diverse populations are needed to address this issue. Here we describe the pilot phase of the GenomeAsia 100K Project. This includes a whole-genome sequencing reference dataset from 1,739 individuals of 219 population groups and 64 countries across Asia. We catalogue genetic variation, population structure, disease associations and founder effects. We also explore the use of this dataset in imputation, to facilitate genetic studies in populations across Asia and worldwide.

## Abstract (web summary)

Using whole-genome sequencing data from 1,739 individuals, the GenomeAsia 100K Project catalogues genetic variation, population structure and disease associations to facilitate genetic studies in Asian populations and increase representation in genetics studies worldwide.

## Main

The underrepresentation of non-European individuals in human genetic studies^1^ limits the applicability of the results for a large proportion of the world’s population^2^. Reference genome datasets^3–12^ are needed to characterize population-specific variation, enable efficient imputation of variants that are not directly genotyped, and extend genome-wide association studies (GWAS) to additional populations. The value of population-specific reference datasets is well recognized and projects based in the United States and Europe have provided deep characterization of specific populations (for example, Ashkenazi Jews^12^ and individuals from the Netherlands^3^ and Iceland^13^) and, in particular, data from individuals of Nordic countries have provided examples of how reference genome datasets can be used to drive comprehensive genetic studies across an entire population^14^. In Africa, populations show complex genetic patterns, smaller blocks of linkage disequilibrium and higher levels of heterozygosity, which provides unique value for genetic studies. Across the continent, early reference genome datasets for diverse populations are being built as part of H3Africa and other studies^5,15^. A Korean reference genome as well as Japanese and Chinese reference genome datasets have been created, and the formation of large biobanks such as BioBank Japan^16^ and the China Kadoorie Biobank^17^ will accelerate the pace of discovery of disease associations across east Asia.

A shared recognition of the value of coordinated efforts and the need for reference genome datasets that would be useful for the complex populations of Asia has led to the formation of the GenomeAsia consortium (http://www.genomeasia100k.com). The consortium serves to facilitate and coordinate sequencing efforts among consortium members to maximize the value of the genomic sequence data that is produced and to facilitate efforts by national or other regional groups. Here we describe the GenomeAsia Pilot (GAsP) project, which consists of analyses of the whole-genome sequencing data of 1,739 individuals from 219 population groups across Asia, with the ultimate goal of providing a useful genomic resource and facilitating genetic studies in Asia. We use the data that was generated in this pilot to analyse population structure and history, and as the basis for designing larger-scale genomic studies. Furthermore, we explore disease-associated loci as an initial comparison of differences between populations. We show that the variant data produced by this project improve variant filtering for the discovery of disease-associated genes of rare diseases. We show that Asia has sizable founder populations and that further studies in these populations may be useful for the discovery of rare-disease-associated genes. We also report an initial survey of loss-of-function alleles found in the GAsP project.

## The GAsP dataset

**Fig. 1:**
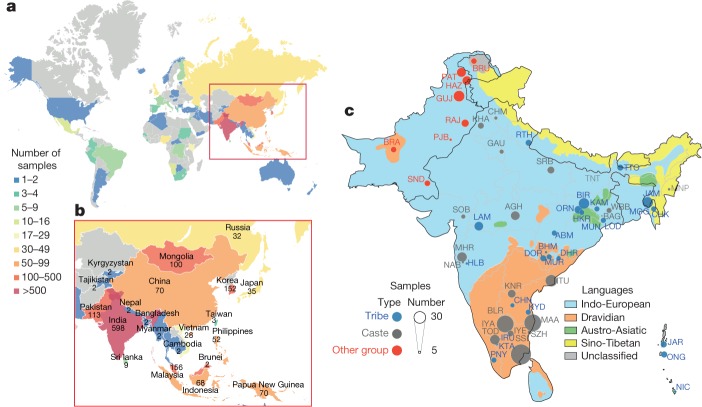
Sampling distribution of GAsP. **a**, **b**, Sample sizes. **c**, Location, language and social hierarchy associated with samples from south Asia. Groups with fewer than three samples are not plotted. See Supplementary Table 1a for definitions and descriptions of samples and population groups included in each geographically defined set.

For the GAsP project, we generated 1,267 high-coverage (average 36×) whole-genome sequences and analysed these together with 596 publicly available human genome sequences from previous sequencing studies (Supplementary Information 1, 2 and Supplementary Tables 1a–c, 2a). The 1,739 samples were enriched for individuals from population isolates to capture the broadest wealth of genetic diversity; the dataset includes 598 sequences from India, 156 from Malaysia, 152 from South Korea, 113 from Pakistan, 100 from Mongolia, 70 from China, 70 from Papua New Guinea, 68 from Indonesia, 52 from the Philippines, 35 from Japan and 32 from Russia (Fig. 1a–c and Supplementary Table 1a–c). To facilitate comprehensive and comparative analysis of human genetic variation, we included sequencing data from African, European and American samples (Supplementary Table 1a, b). The sequenced samples originate from 7 global regions, 64 different countries of origin and 219 population groups. About 80% of the samples come from Asia and emphasize population groups that are underrepresented in previous genetic studies (Fig. 1a, b, Supplementary Tables 1a–c, 2b and Supplementary Information 1, 2). Each global region and population group was assigned a unique three-letter code for future reference (see Supplementary Table 1a for three-letter code designations). Within Asia, the sampling of many distinct population groups allowed us to analyse the relationship between geography, physical characteristics and genetic variation. In south and southeast Asia, in particular, we sampled across diverse populations to gather new insights into how groupings defined on the basis of caste and language relate to genetic diversity, admixture with extinct hominins and other genetically described characteristics.

## Population structure

**Fig. 2:**
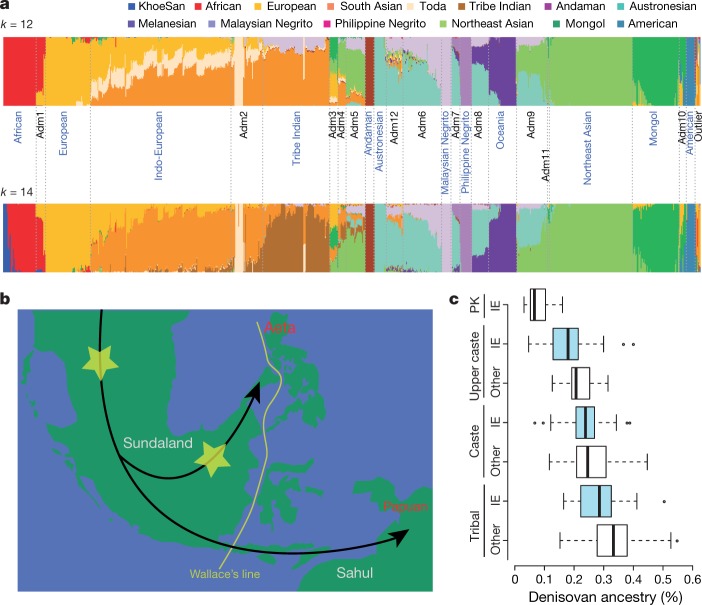
Population structure and admixture. **a**, ADMIXTURE plots for *k* = 12 and *k* = 14 illustrating the identification of 12 reference groups. **b**, Proposed modern human migration route into southeast Asia during the Last Glacial Maximum with potential locations of Denisovan admixture (yellow asterisks). Green indicates the above water landmass at the glacial maximum and white outlines indicate present-day shorelines. **c**, Estimates of Denisovan ancestry in south Asians, stratified by social/cultural group and language. IE, Indo-European. Adivasi Indo-European, *n* = 30; Adivasi non-Indo-European, *n* = 196; caste Indo-European, *n* = 68; caste non-Indo-European, *n* = 155; upper caste Indo-European, *n* = 49; upper caste non-Indo-European, *n* = 19; Pakistani Indo-European, *n* = 79. The centre line indicates the median; box limits show the middle 50%; whiskers extend two standard deviations from the mean; points are outliers.

**Extended Data Fig. 1:**
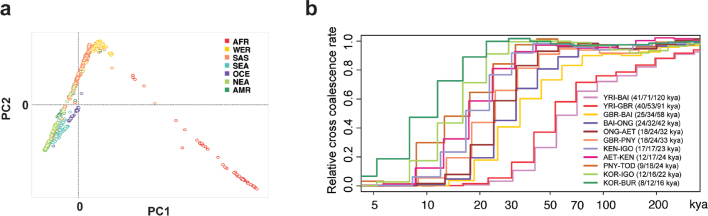
Diversity and divergence times of GAsP samples. **a**, PCA plot of study samples. Africa (AFR), *n* = 102; West Eurasia (WER), *n* = 111; South Asia (SAS), *n* = 642; Southeast Asia (SEA), *n* = 162; Oceania (OCE), *n* = 68; Northeast Asia (NEA), *n* = 346; Americas (AMR), *n* = 26. The samples included in each of these geographically defined groups are described in Supplementary Table 1a. **b**, MSMC cross-coalescence rates showing divergence time estimates between different groups. The point estimate of the date was given at which 25%,50% and 75% of lineages in the pair of populations have coalesced into a commonancestral population.

Knowledge of the complex history of Asian populations informs optimal sampling for larger-scale biomedical sequencing efforts. We applied standard approaches for detecting recent positive selection, quantifying the population structure and inferring the history of the different populations, including principal component analysis^18^, multiple sequentially Markovian coalescent (MSMC)^19^, ADMIXTURE^20^, *F*_ST,_ uniparental analyses and the analysis of the Y chromosome and mitochondrial haplogroups (Fig. 2, Extended Data Fig. 1 and Supplementary Information 3–10). Our results generally recapitulate the broad inferences of previous studies, and ADMIXTURE plots show complex structure within south and southeast Asia (Fig. 2a). In particular, India, Malaysia and Indonesia contain multiple ancestral populations as well as multiple admixed groups. On the basis of MSMC cross-coalescence rates, which reflect the increase in coalescence times of haplotypes sampled from different populations relative to haplotypes sampled from the same population^19^, we estimate that the oldest population splits in southeast Asia and Oceania involve Melanesians and/or Negritos, who show a substructure from approximately 40 thousand years ago and evidence of separation around 20–30 thousand years ago (Extended Data Fig. 1b and Supplementary Information 3). The population structure provides genetic information on classically defined population groups to aid future studies. For example, using multiple analytical approaches (Supplementary Information 3, 6), we confirmed that the anthropologically classified ‘Negrito’ groups from India, Malaysia and the Philippines, are genetically more closely related to their geographical neighbours than they are to other Negrito groups^21,22^, suggesting that dark skin colour is probably an environmental adaptation (for example, to high levels of solar radiation) and not an indicator of shared ancestry.

Our dense sampling of Asian populations enables the examination of Denisovan admixture in greater detail than has been previously possible, providing information about population splits or in-flows that occurred at or after the time of admixture (Supplementary Information 10). Our estimates of Denisovan ancestry were highest in Melanesians and the Aeta, intermediate in the Ati and groups from the Indonesian island of Flores, and low (but still significantly greater than 0) in most south, east and southeast Asian populations. We found high levels of Denisovan ancestry in Philippine Negrito groups but not in Malay or Andaman Negritos; these results are qualitatively similar to what was found in a previous study that was based on single-nucleotide polymorphism (SNP) arrays^23^. The high levels of Denisovan ancestry in Melanesians and the Aeta are consistent with an admixture event into a population that is ancestral to both^23^; however, two lines of evidence suggest that the ancestors of the Aeta experienced a second Denisovan admixture event. First, multiple analyses found that the Aeta are genetically more similar to populations without appreciable Denisovan ancestry (for example, Igorot, Malay and Malay Negrito groups) than they are to Melanesians (Supplementary Information 3, 6). This can be explained by more recent gene flow from other populations without Denisovan ancestry. However, such gene flow would reduce the levels of Denisovan admixture below that found in Melanesians. More directly, we find that putative Denisovan haplotypes that are unique to the Aeta (*n* = 962) are significantly longer than putative Denisovan haplotypes shared between Aeta and Papuans (*n* = 596, mean = 16.1 kb compared with mean = 14.1 kb, Mann–Whitney *U*-test, *P* ≪ 10^−10^), or putative Denisovan haplotypes unique to Papuans (*n* = 727, mean = 16.1 kb compared with mean = 14.9 kb, Mann–Whitney *U*-test, *P* ≪ 10^−1,000^) (Supplementary Information 10), supporting a scenario in which a second admixture event between the Aeta and Denisovans happened after the separation of the Aeta and Melanesians. Two distinct Denisovan admixture events are most consistent with *Homo sapiens* and Denisovans interacting within southeast Asia^23^, making it likely that admixture occurred within Sundaland (Fig. 2b) or even farther east^24,25^.

A recent study found a slightly increased amount of Denisovan ancestry in south Asians compared with a priori expectations^26^. We examined whether this was correlated with either language or social and/or caste status. South Asian samples were grouped into individuals who speak Indo-European languages and individuals who speak non-Indo-European languages (excluding individuals who speak Tibeto-Burman languages), as well as four social or cultural groups: tribal (Adivasi) groups, lower-caste groups, high-caste groups and Pakistani groups (Indo-European language speaking only). We found that the average levels of Denisovan ancestry were significantly different between the four social or cultural groups (Mann–Whitney *U*-test, *P* < 10^−8^ for all pairwise comparisons; Fig. 2c and Supplementary Information 10). Our results are consistent with the scenario that Indo-European-speaking migrants who entered the subcontinent from the northwest admixed with an indigenous South Asian (ancestral south Indian)^27,28^ group who had higher levels of Denisovan ancestry.

## Medical relevance

**Extended Data Fig. 2:**
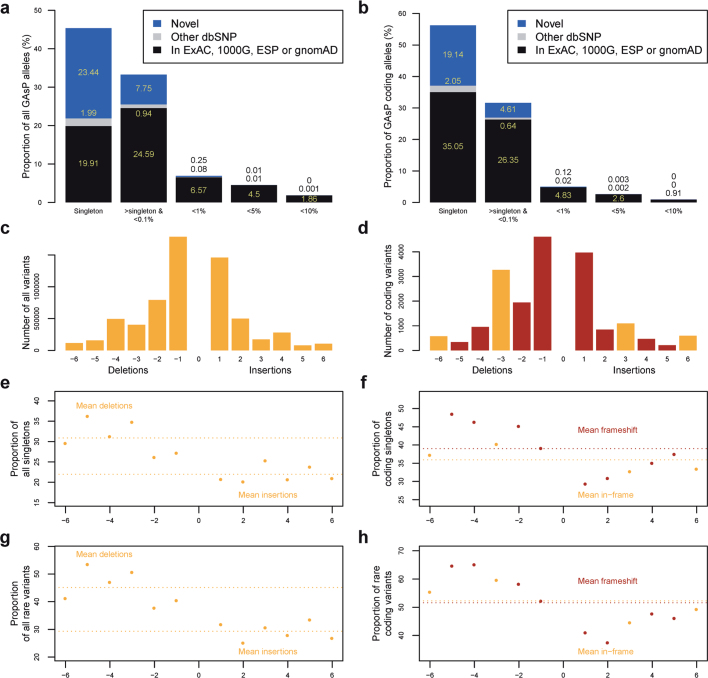
Characteristics of GAsP SNPs and indels. **a**, **b**, Comparison of all GAsP variants (**a**) or coding variants (**b**) with gnomAD, ExAC, 1000 Genomes, ESP and dbSNP data as a function of the MAF within the GAsP dataset. **c**, **d**, The number and lengths of small indels in the genome (**c**) or coding regions (**d**). **e**–**h**, Proportion of non-coding (**e**, **g**) or coding (**f**, **h**) indels that were singletons (**e**, **f**) or rare (allele frequency of <0.1%; **g**, **h**).

We evaluated the use of GAsP dataset in disease-associated genetic studies and medically relevant applications to determine how the results of larger continuing GenomeAsia studies can be used to improve human health (Supplementary Table 4a). We annotated high-quality variants using public databases including ExAC (Exome Aggregation Consortium)^29^, gnomAD^29^, 1000 Genomes Project^4^, ESP (NHLBI GO Exome Sequencing Project)^30^ and dbSNP (Extended Data Fig. 2) and focused on coding-sequence variants. Overall 23% of protein-altering variants in GAsP were not found in these data sources. As expected the majority of coding variants were singletons or very rare (Extended Data Fig. 2). However, the absolute numbers of novel variants with a minor allele frequency (MAF) ≥ 0.1% within our pan-Asian dataset is large (*n* = 194,585), and these are frequent enough to be of relevance for large-scale genetic association studies. We also searched for variants present at low frequency in the overall dataset that are present at significantly higher allele frequencies in one or more of the population groups. We found an additional 144,329 novel variants with MAF > 1% in the full GAsP dataset that were present at a frequency of greater than 1% within populations grouped by geography; South Asia, Southeast Asia, Northeast Asia or Oceania (see Supplementary Table 1a for description of samples and population groups included in each geographically defined set). These geographical regions contain many diverse population groups, and additional studies are needed to characterize patterns of genetic variation in these groups and disease relevance.

**Fig. 3:**
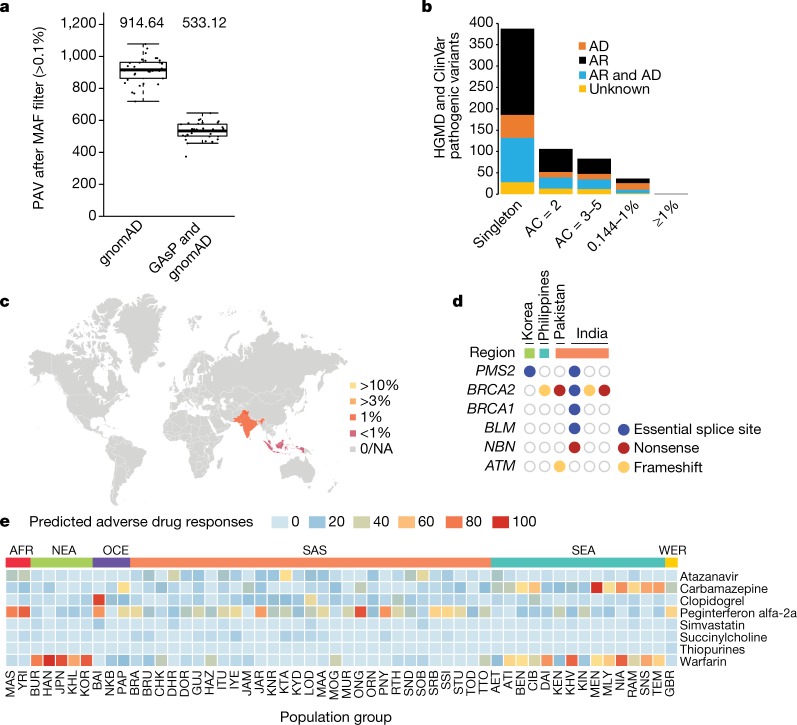
Disease-relevant variant discovery. **a**, Filtering using the GAsP dataset improves candidate variant discovery by removing population specific variants (*n* = 152). The centre line indicates the median; box limits show the upper and lower quartiles; whiskers extend 1.5× the interquartile range. **b**, Allele count (AC) and frequency distribution of variants in the GAsP dataset that are designated disease-causing in the Human Gene Mutation Database (HGMD) or pathogenic in ClinVar. Autosomal-dominant (AD) or autosomal-recessive (AR) or other (unknown) classification as per OMIM. A number of variants (*n* = 37) that had previously been reported to be pathogenic are found in the GAsP study dataset at high frequency and were reclassified (Supplementary Table 4d). **c**, Frequency of β-thalassaemia variant (chromosome 11:5248155 c.92+5G>C) across Asia shows a geographical enrichment. MAF in South Asia is 1.4%. NA, not available. **d**, Novel cancer-predisposing variants identified in the GenomeAsia dataset. **e**, Population-specific probabilities of adverse drug reactions predicted from the aggregate allele frequencies of known variants associated with response to the indicated drugs.

In rare disease genetics, databases are used to filter based on allele frequency with the idea that common alleles are unlikely to be responsible for rare highly penetrant disorders; however, in the absence of appropriate population reference datasets, allele frequencies can be misclassified and may lead to false disease associations^31^. We explored whether the GAsP variant dataset can improve the ability to identify disease-relevant variants in Asian cohorts. We analysed 152 exomes from individuals participating in the Indian Maturity Onset Diabetes in the Young (MODY) project. When both the gnomAD and GAsP datasets were used for filtering (MAF > 0.1%), we reduced the set of remaining candidate variants by approximately twofold in comparison to using the gnomAD dataset alone (Fig. 3a). In this process, we identified a common population polymorphism in *NEUROD1* (H241Q) that is probably benign but that was previously reported to be medically relevant^32,33^. We annotated variants that were identified in the GAsP dataset against the Human Gene Mutation Database (HGMD) disease-causing pathological and ClinVar pathogenic variants. This analysis identified 732 variants (686 SNPs and 46 insertions or deletions (indels)) in 514 genes (Fig. 3b, Supplementary Table 4b, c and Supplementary Information 11). We compared the 732 pathogenic variants against the gnomAD, ExAC^29^, 1000Genomes^4^, ESP^30^, dbSNP^34^, ALSPAC, TwinsUK^35^ and 1000Japanese^6^ databases to remove variants that occurred at >1%, focused on those with allele frequencies >0.15% in GAsP (38 variants), and reviewed them against the criteria defined by the American College of Medical Genetics (ACMG). This resulted in reclassification of 11 of the 38 variants (Supplementary Table 4d). We examined the geographical distribution of the remaining, revalidated but high-frequency, pathogenic disease-associated variants. As expected, most of these variants were highly enriched in Asia. For example, an HBB variant (chromosome 11: 5248155 c.92+5G>C) associated with β-thalassaemia is found almost exclusively in south Asians and at a lower frequency in southeast Asians (Fig. 3c).

We also examined our dataset for novel variants in genes known to be associated with cancer risk. We found 13 unique variants in 6 genes from 17 samples. This included frameshift, stop-gained and essential splice-site mutations in *BRCA2* (*n* = 9), *BRCA1* (*n* = 1), *ATM* (*n* = 2), *BLM* (*n* = 1), *NBN* (*n* = 2) and *PMS2* (*n* = 2) (Fig. 3d and Supplementary Table 4e). Of the two *PMS2* essential splice variants, one was found in a Korean sample. Loss-of-function mutations in *PMS2* are associated with mismatch repair defects that lead to a higher risk of cancer development. In a separate study of gall bladder cancer, we found the same essential splice site *PMS2* mutation (chromosome 7:6043690C>G) in a Korean patient whose gall bladder cancer exhibits microsatellite instability (E.W.S. and S. Seshagiri, manuscript in preparation). Identification of genetic variants that affect drug efficacy and safety through the alteration of pharmacokinetics enables application of individualized treatment^36–41^. Variation in drug responses are generally recognized and recommendations for dosing are sometimes guided by apparent or self-reported population identity despite the lack of a rigorous pharmacogenomic basis. We assessed the allele frequencies of key pharmacogenomic variants in our dataset to identify inter-population differences that have potential implications on drug testing and treatment (Fig. 3e, Supplementary Table 4g and Supplementary Information 13).

Carbamezepine, clopidigrel, peginterferon and warfarin showed the largest variation between populations in predicted adverse drug responses with groups ranging from 0 and 100 predicted adverse drug responses. For example, the HLA-B*15:02 variant, associated with risk for development of Steven Johnson syndrome^38^ in patients treated with carbamazepine was found to occur at an increased frequency in Austronesian language-speaking populations from southeast Asia (for example, 63% in the Mentawai of West Sumatra; 46.6% in the Nias of North Sumatra) compared with other groups (Supplementary Information 13). There are roughly 400 million individuals who belong to Austronesian groups that are at increased risk for carbamazepine sensitivity, including the vast majority of the people from Indonesia, Malaysia and the Philippines.

## Founder populations

Population bottlenecks produce strong founder effects and increased rates of recessive disease. In populations with strong founder effects, the loss-of-function variant frequency spectrum is skewed higher, greatly increasing power of association^42^ and providing unique advantages for the identification of genes associated with both rare and complex diseases^43,44^. We followed the approach described in a previous study on south Asian populations to characterize the degree to which genomic segments are inherited as identical by descent (IBD) in population groups in our dataset^45^.

**Fig. 4:**
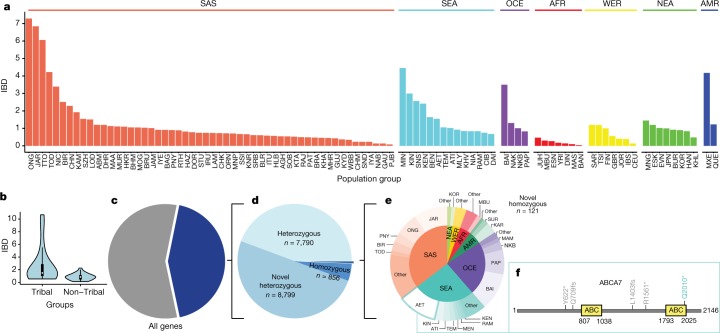
Founder effects and homozygous loss of function. **a**, IBD scores across different population groups are shown for 96 ethnicities (1,417 samples) across global regions. The scores given in the figure are relative ratios compared to that of the Finnish group. **b**, Violin plot showing IBD scores in 29 tribal groups and 25 non-tribal groups consisting of 293 and 336 samples, respectively. The centre line indicates the median; box limits show 1.5× the interquartile range. **c**, Proportion of genes with at least one high-confidence PTV. **d**, Proportion of novel, known, heterozygous and homozygous PTVs in the GAsP dataset. **e**, Pie chart of novel homozygous PTVs plotted by region (inner circle) and population group (outer circle). Groups with less than two PTVs were grouped as other. **f**, Novel homozygous PTV Q2010* (green) found in ABCA7 localizes to the C-terminal ABC domain. Previously reported PVTs are shown in grey.

Our analysis revealed IBD scores of 1.465 and 0.817 for Finnish and British groups, consistent with previous analyses^45^. The IBD score of all of the groups was normalized relative to the Finnish group (Fig. 4a and Supplementary Information 12). Our study includes many groups with small population sizes and it is expected that endogamy paired with small population size will greatly increase IBD scores. We found that indigenous and tribal groups had IBD scores that were skewed upwards from non-tribal groups (Fig. 4b). Notably, we found that a number of Asian groups with large urban populations have IBD scores above or close to that of the Finnish population. For example, samples from an outpatient hospital in Chennai, a city with a census size of 9 million, had an IBD score that was approximately 1.3 times greater than the score for the Finnish group.

## Human knockouts

Homozygous loss-of-function alleles found in humans give us the opportunity to assess the phenotypic effect of specific gene loss and can provide important information about opportunities for treating disease^46,47^. To assess the contents of our dataset, we examined high-confidence protein-truncating variants (PTVs). We found 17,566 PTVs with at least 1 PTV in approximately 43% of all protein-coding genes (*n* = 8,766; Fig. 4c). Among the PTVs, most were heterozygous variants unique to our dataset (*n* = 8,799; Fig. 4d), similar to the PTV data from ExAC^25^ (67% singletons). A smaller number were homozygous and had been reported in gnomAD, dbSNP or 1000 Genomes Project (*n* = 856). In addition, within our dataset were 121 homozygous PTVs that have not previously been reported (Supplementary Table 5). These novel homozygous PTVs were mostly found in groups with high IBD scores such as the Jarawa and Onge from the Andaman Islands (Fig. 4e). The novel homozygous PTVs include an allele of the *ABCA7* gene, Q2010*, that is found in only the Aeta population (Fig. 4f). Heterozygosity for loss-of-function alleles of *ABCA7* has been shown to increase susceptibility to Alzheimer’s disease in European populations^48^.

## Imputation panel

We carried out preliminary work to evaluate the utility of the pilot dataset for imputation. For this analysis, we downsampled whole-genome sequence data from South Asian, Southeast Asian and Northeast Asian population groups (see Supplementary Table 1a for samples included in each of these geographically defined sets) 30× to the genotypes represented on the Illumina Global Screening Array v.1 genotyping array, and compared the imputation using either phase 3 of the 1000 Genomes Project or the GAsP reference panels. We found, as described by Illumina, that imputation accuracy of the 1000 Genomes Project reference panel is consistently well below 90% for east Asian and south Asian samples whereas using the GAsP reference panel we achieved accuracies ranging from 93 to 95%. To accelerate evaluation and broad utility, we have placed the data on the Michigan Imputation Server (https://imputationserver.sph.umich.edu/index.html).

## Discussion

Understanding the genetic basis of human disease will benefit from an increase in the number and scale of disease-association studies that are carried out in Asian populations. In the pilot phase of the GenomeAsia project, the sample set that we analysed allowed us to address a wide range of questions regarding the history of specific Asian population groups and to map out strategies for additional sequencing efforts. We plan for a staged and coordinated approach, to include the generation of genomic population-specific reference datasets and imputation panels, and use this approach for the production of custom SNP arrays as a catalyst for disease-association studies. This approach is particularly useful in founder populations, such as recent studies in the founder populations of Finland^49^, as well as other populations. This will be particularly valuable in Asia^14,50^, which has founder effects that have not only previously been demonstrated in isolated populations, but are also evident in major urban centres.

Analysis of the GAsP dataset allows us to map out strategies for efforts focused on specific population centres in Asia as well as the generation of important tools that will increase our understanding of how genetic variants affect disease susceptibility and drug responses. The dataset improves the ability to filter out low-probability candidates for highly penetrant disorders, to identify putatively pathogenic variants that are found at high frequency in particular populations and improve the ability to infer pathogenicity of identified variants. The identification of novel homozygous PTVs in this study expands the catalogue of genes in which homozygous loss of function appears to be tolerated and, when combined with phenotype information, this will provide important biological insights into gene function. The ability to define gene function in humans through the study of the phenotypic effects of loss-of-function mutations is becoming an increasingly valuable approach^51^ and the study of additional variants and populations in which homozygosity occurs at high rates will add to the global resources for carrying out human knockout studies.

## Methods

### Data reporting

No statistical methods were used to predetermine sample size. The experiments were not randomized and the investigators were not blinded to the allocation during analysis.

### Samples

We accessed publicly available high-coverage, whole-genome FASTQ files from previous studies of human genetic variation^52–55^ and combined these with 1,267 high-coverage genomes generated as part of this project. Full details on the samples chosen for sequencing and the informed consent processes for these samples can be found in Supplementary Information 1. We restricted our analyses to genomes generated using Illumina short-read sequencing technology.

### Whole-genome sequencing

Whole-genome sequencing libraries were prepared using standard protocols (Illumina) and sequenced on Illumina Hiseq 2500/4000 or X10 machines. We obtained paired-end (2 × 100 bp or 2 × 150 bp) for each sample.

### Filtering, alignment and variant calling

We aligned the Illumina short-read sequences to the GRCh37+decoy reference genome with BWA-mem^56^ using the default parameters. Putative PCR duplicates were flagged using SAMBLASTER^57^. The SAM outputs were converted to BAM format, and sorted by chromosomal coordinates using Sambamba^58^, and all BAM files for the same samples were merged.

The sex of the samples was inferred from the coverage of the autosomes and the sex chromosomes, and confirmed from the submitted metadata with the samples. All samples that had an average coverage less than 20-fold or for which we found a difference in the inferred and reported sex were removed from further analysis. We used verifyBamID^59^ to identify contamination using the chip-free mode and samples for which swaps or contamination was identified were removed from subsequent analyses. A contamination level of 3% was used as a cut-off, and this left us with 1,739 samples that were used for all downstream analyses.

We identified the single-nucleotide substitutions and small indels variants in the 1,739 samples using the reference model (gVCF-based) workflow for joint analysis in GATK^60^. Variants were called individually in each sample using the HaplotypeCaller in ‘-ERC GVCF’ mode to produce a record of genotype likelihoods and annotations at each site in the genome. Multi-allelic variants are reported in the GenomeAsia browser but were not included in the analysis. A gVCF file was created for every sample, and a subsequent joint genotyping analysis of all gVCFs was done to identify the variants in the cohort. We followed the GATK-recommended best practices for variant recalibration to create a final VCF file and recalibrated the variants to select 99% of the true sites from the training set for VQSR^61^. The VCF files were zipped using bgzip and indexed using tabix.

### Identification of first-degree relative pairs

Several of the reported analyses require filtering to remove related samples. We used KING^62^ to identify such first-degree relative pairs. We first used vcftools^63^ and plink^64^ to convert the VCF file into the required input format for KING. The estimated kinship coefficient was restricted to 0.177–0.354 as described in the KING manual to identify the first-degree relative pairs, and the results were confirmed from the submitted metadata. The number of unrelated samples by country-of-origin is shown in Supplementary Table 1.1.

### Quantifying population structure and changes in population size

We restricted our attention to 7,966,132 autosomal markers (that is, SNPs) with MAF ≥ 0.01 and call rate ≥ 98%. In some analysis, severe linkage disequilibrium pruning was applied as follows: sliding windows of size 50 (that is, the number of markers used for linkage disequilibrium testing at a time) and window increments of 5 markers; for any pair of SNPs in a window, the first marker of the pair was discarded if *r*^2^ > 0.2. After linkage disequilibrium pruning, 1,089,227 SNPs were retained for analysis. All data-filtering procedures were conducted in PLINK v.1.9^64^.

Analyses of population structure was performed using the quality-control-positive linkage-disequilibrium-pruned set of 1,089,227 autosomal SNPs. Principal component analysis (PCA)^18^ was conducted across all available populations in EIGENSTRAT v.6.1.4. Results were visualized in Tableau v.9.3. We applied unsupervised hierarchical clustering of individuals using the maximum likelihood method implemented in ADMIXTURE v.1.3.0^20^ using default input parameters. The ‘--cv’ flag was adopted to perform the cross-validation procedure and to calculate the optimal *k* value.

We used MSMC^5^ to estimate changes in population size and split times. This analysis used two different phased genome datasets (using Shapeit v.2^65^ and Eagle2^66^). The details for the phasing are described in Supplementary Information 4. Chromosome 6 was excluded from the analysis owing to possible phasing errors in the HLA region. We used four haplotypes (two individual genomes) for estimating changes in population size in a population and eight haplotypes (two genomes from each of a pair of populations) for the estimation of population split times. We assumed a mutation rate of μ = 1.25 × 10^−8^ per site per generation and an average generation time of 29 years, as in previous studies^8,19^.

### Comparison with 1000 Genomes Project genotype calls

We filtered the variant calls to include only biallelic SNPs with <10% missing genotype calls that were within the 1000 Genomes Project strict mask (available at ftp://ftp.1000genomes.ebi.ac.uk/vol1/ftp/release/20130502/supporting/accessible_genome_masks/20141020.strict_mask.whole_genome.bed). Then, for each of the 119 overlapping samples considered individually, we calculated variant discordance rates for those filtered SNPs that (1) had a genotype call in both the 1000 Genomes Project data and the GAsP data; and (2) had a ‘variant’ call (that is, a non-homozygous reference genotype call) in at least one of the datasets. These discordance rates were then stratified by the estimated MAF in the GAsP dataset.

### Patterns of allele sharing

We used a parsimony-based analysis of allele sharing^55^ that focused on SNPs that were not present in sub-Saharan Africans or in archaic humans (further details are provided in Supplementary Information 8).

### Archaic admixture

We used a method similar to the ‘enhanced’ *D*-statistic approach^8,67^ to estimate levels of Neanderthal and Denisovan ancestry in each non-African sample. The estimates were calibrated assuming 0% Denisovan ancestry in the British population, 4% Denisovan ancestry in the Papuan population and 2% Neanderthal ancestry in the British population (full details are provided in Supplementary Information 9).

### Determination of high-quality variants for medically related analyses

High-quality variants were defined as variants that (1) had a read-depth ≥ 5 and genotype-quality ≥ 20; (2) were contained in the high-confidence regions as described by Genome in a Bottle (ftp://ftp-trace.ncbi.nlm.nih.gov/giab/ftp/release/NA12878_HG001/NISTv3.3.2/GRCh37/supplementaryFiles/HG001_GRCh37_GIAB_highconf_CG-IllFB-IllGATKHC-Ion-10X-SOLID_CHROM1-X_v.3.3.2_highconf.bed) and (3) passed the gnomAD_Filter. Variant annotation was carried out using SnpEff^68^ (v.4.1).

### IBD scores

Groups with at least two samples were considered for analysis. We restricted our analysis to genomic regions with high-confidence calls and removed related samples based on reported relationship, kinship, PCA and IBD analyses. The scores given in the figure are relative ratios compared to that of the Finnish group.

### PTVs

PTVs are defined as high-quality variants that were annotated as having a strong impact on the protein (such as frameshifts, essential splice sites or premature stop codons). We restricted calls to high-confidence regions determined by Genome in a Bottle as described above and filtered for high-confidence PTVs using the LOFTEE program^69^. We used a similar strategy for additional filtering of variants as proposed previously^47^ and flagged variants with ≤7 reads covering the variant site; ≤80% of reads had the variant, were not in the bottom 1 percentile of phyloP or gerpRS^65^ scores and for which the affected transcripts made up less than 50% of all expression as specified by GTEx.

### Enriched medically relevant variants

We compared variant allele counts for Asian and Oceania samples from the GenomeAsia cohort to allele counts present in non-Asian gnomAD samples (European (non-Finnish), European (Finnish), Latino, African or other) for variants found in a set of 124 medically relevant genes. The genes used were 115 genes used for prenatal screening^70^ as well as the cancer-associated genes *BRCA1*, *BRCA2*, *TP53*, *MEN1*, *MLH1*, *MSH2*, *MSH6*, *PMS1* and *PMS2A*. A Fisher’s exact test was used to calculate variations that were significantly overrepresented in the GenomeAsia subsamples and corrected for multiple testing using the Bonferroni method. We further accessed variants for these genes that had not previously been reported. All variants were further filtered as being damaging as determined by having a high impact on the protein (stop codon, essential splice site or frameshift mutation) or were predicted to be damaging by the Polyphen2 program. A cumulative comparison of allele counts for all over-represented and novel variants was performed and compared to non-Asian gnomAD to calculate a *P* value, odds ratio and relative difference in cumulative allele frequency (GenomeAsia cumulative allele frequency minus gnomAD non-Asian allele frequency). Reported *P* values were corrected for multiple testing using the Bonferroni method.

### Reporting summary

Further information on research design is available in the Nature Research Reporting Summary linked to this paper.

## Online content

Any methods, additional references, Nature Research reporting summaries, source data, extended data, supplementary information, acknowledgements, peer review information; details of author contributions and competing interests; and statements of data and code availability are available at 10.1038/s41586-019-1793-z.

## Supplementary information

Supplementary Information This file contains Supplementary Notes 1-13

Reporting Summary

Supplementary Tables

## Data Availability

For each variant, summary data for genotype quality, allele depth and population-specific allele counts were calculated before removing all genotype data. This dataset is available without requirement for login or other form of restriction for browsing or for download at https://browser.genomeasia100k.org. Individual level VCF data files representing the 1,180 newly sequenced genomes from individuals of 74 population groups are freely available to any qualified investigator without restriction. Chinese samples sequenced were from Corriell cell lines and are not subject to Chinese government regulation. The data are also available from the European Genome Archive (EGA) under accession number EGAS00001002921. The procedure for accessing individual level data are as follows: access forms can be obtained from the GenomeAsia website (https://browser.genomeasia100k.org), and once filled out and sent to dataaccess@genomeasia100k.org the request will undergo administrative review and instructions for downloading the data will be returned to the requestor. Access to individual level data from Malaysian samples are subject to additional restrictions. The complete dataset of sequences of unrelated individuals (1,667 samples) has been phased and can be used for imputation through the Michigan Imputation Server (https://imputationserver.sph.umich.edu/index.html). The goal of the GenomeAsia100K consortium is to facilitate and accelerate genetic studies in Asian populations by coordinating sequencing efforts among its members. To achieve this goal, we are committed to continuing to make data publicly available and accessible. As data are contributed to the consortium by individual members, it will be made immediately available in summary form or as imputation reference panels where appropriate. Data will be made available in individual form wherever possible and not limited by the bounds of informed consent, national privacy laws and regulations, or other external restrictions that may apply.
