# Supplementary material for: The GenomeAsia 100K Project enables genetic discoveries across Asia
Source: Nature. 2019 Dec 4;576(7785):106–11. doi: 10.1038/s41586-019-1793-z (PMC7054211; doi:10.1038/s41586-019-1793-z)
Supplement: Supplementary file 1 — This file contains Supplementary Notes 1-13 [file 41586_2019_1793_MOESM1_ESM.pdf]

nature

https://doi.org/10.1038/s41586-019-1793-z

Supplementary information

The GenomeAsia 100K Project enables genetic discoveries across Asia

In the format provided by the authors and unedited

GenomeAsia100K Consortium

Supplementary Information

Title		Page
S1	Samples, Consent & Sequencing	02
S2	Mapping, filtering and variant calls	09
S3	Phasing and MSMC	16
S4	Population structure and admixture	26
S5	Fst analysis	34
S6	Using patterns of allele sharing to construct population trees	37
S7	Selective sweep	42
S8	Analyses of the non-recombining portion of the Y chromosome	46
S9	Mitochondrial and Y-chr distribution in population groups	50
S10	Estimating Neanderthal and Denisovan ancestry	57
S11	Identification of disease-causing variants in GAsP dataset	67
S12	IBD analysis	70
S13	Allele frequencies of key pharmacogene variants	73

Supplementary note S1 – Samples, consent and sequencing

R. Rand Allingham[1], Khai C. Ang[2], Keith C. Cheng[2], Arkasubhra Ghosh[3], Seik Soon Khor[4], Byung Ju Kim[5], J. Stephen Lansing[6], Changhoon Kim[7], Partha P. Majumder[8], Badrul M. Md-Zain[9], Syet Q. Mehdi[10], Viswanathan Mohan[11], Madasamy Parani[12], Jeong-Sun Seo[5,7,13], Jong-Yeon Shin[14], Herawati Sudoyo[15], Katsushi Tokunaga[4], Radha Venkatesan[11], Jeffrey D. Wall[16] and Stephan Schuster[17]

authors responsible for this section: Jeff Wall - Jeff.Wall@ucsf.edu and Stephan Schuster - SCSchuster@ntu.edu.sg

[1]Department of Ophthalmology, Duke University Medical Center, Durham, North Carolina 27710, USA. [2]Department of Pathology and Jake Gittlen Laboratories for Cancer Research, Penn State College of Medicine, Hershey, Pennsylvania 17033, USA. [3]GROW Research Laboratory, Narayana Nethralaya Foundation, Bangalore, Karnataka 560010, India. [4]Department of Human Genetics, University of Tokyo, Tokyo 113-0033, Japan. [5]Precision Medicine Institute, Macrogen Inc., Gyeonggi-do 13605, Korea. [6]Complexity Institute, Nanyang Technological University, Singapore 639798. [7]Bioinformatics Institute, Macrogen Inc., Seoul 08511, Korea; and Gong-Wu Genomic Medicine Institute (G2MI), Seoul National University Bundang Hospital, Gyeonggi-do 13605, Korea. [8]National Institute of BioMedical Genomics, Netaji Subhas Sanatorium, Kalyani 741251, West Bengal, India; and Human Genetics Unit, Indian Statistical Institute, Kolkata, West Bengal 700108, India. [9]School of Environment and Natural Resource Science, Faculty of Science and Technology, Universiti Kebangsaan Malaysia, 43600 Bangi, Selangor, Malaysia. [10]Center for Human Genetics, Sindh Institute of Urology and Transplantation, Karachi 74200, Pakistan. [11]Madras Diabetes Research Foundation and Dr. Mohan's Diabetes Specialties Centre, Chennai, Tamil Nadu 600086, India. [12]Department of Genetic Engineering, SRM University, Kattankulathur, Tamil Nadu 603203, India. [13]Departments of Biomedical Sciences, Seoul National University Graduate School, Seoul 03080, Korea. [14]Department of Clinical Diagnosis, Macrogen Inc., Seoul 08511, Korea. [15]Genome Diversity and Diseases Laboratory, Eijkman Institute for Molecular Biology, Jakarta 10430, Indonesia. [16]Institute for Human Genetics, University of California, San Francisco, California 94143, USA. [17]Singapore Centre for Environmental Life Sciences Engineering, Nanyang Technological University, Singapore 637551.

A full list of samples included in this project is provided in Supplemental Table 1 (which can be downloaded separately). This table also includes information on country-of-origin, 1st degree relatives, estimated archaic ancestry proportions and homozygous PTVs. Specific information on the new samples obtained from different populations is

provided below. The table provides the author responsible for the specific samples.

KOR, BUR, MNG and KHL: We recruited 150 unrelated Koreans and 100 Mongolians from existing studies. The Korean samples (with Korean ancestry) were selected from studies with IRB numbers C-1705-048-852, C-1701-131-828 and 0806-023-246. For the Mongolian subjects, 87 Buryats, 12 Khalkhs and 1 with unknown ancestry (assumed to be Mongolia), were recruited from the GENDISCAN project, where 2,008 volunteers were recruited in Dashbalbar, Dornod Province, Mongolia, a geographically isolated region in Northeast Asia (IRB Number H-0307-105-002). For each study, Informed consents were obtained from all study subjects and the study protocols were approved by IRB of the Seoul National University Hospital.

BLR: Samples from type 2 diabetes patients undergoing treatment for diabetic retinopathy were obtained from the outpatient population of an eye hospital in Bangalore. The study was approved by the Institutional Ethics Committee of Narayana Nethralaya and adhered to the tenets of the Declaration of Helsinki. All samples were obtained with written informed consent of the subjects.

MAA: Type 2 diabetic subjects were recruited from Dr. Mohan's Diabetes Specialties Centre, a large diabetes center in Chennai (formerly Madras) city in southern India, which has a population of about 6 million people. All patients underwent a structured assessment including detailed family history.

The samples were obtained under appropriate informed consent with study review and approval obtained from the local human studies review panel. The reported investigations have been carried out in accordance with the principles of the Declaration of Helsinki.

Consented and de-identified patients' blood samples were used for extraction of DNA. EDTA anti-coagulated venous blood samples were collected from all study subjects, and the genomic DNA was isolated from whole blood by proteinase K digestion followed by phenol-chloroform extraction. Subsequently genomic DNA was precipitated in ethanol. The quality and quantity were assessed spectrophotometrically.

GBR, DAI, KHV, STU, ITU, YRI, HAN and MAS: DNA samples from de-identified individuals from the International HapMap and 1000 Genomes Projects were purchased from the Coriell Institute for Medical Research.

MEN, NIA, BEN, CIB, RAM, BAI(GA000500 – GA000516) and PAP (GA000518, GA000519, GA000521 and GA000523): The Indonesian samples used in this study were collected by J. Stephen Lansing, Herawati Sudoyo, and a team from the Eijkman

Institute for Molecular Biology, with the assistance of Indonesian Public Health clinic staff. All collections followed protocols for the protection of human subjects established by institutional review boards at the Eijkman Institute, Nanyang Technological University, and the University of Arizona. Permission to conduct research in Indonesia was granted by the State Ministry of Research and Technology. Genotyping and analyses of newly reported non-Indonesian samples were approved by the institutional review board at the University of Arizona. The generation of whole genome sequencing data for the samples was approved by Nanyang Technological University institutional review board (IRB-2014-12-011). The non-Indonesian samples were donated by collaborators for the purpose of academic research. Details regarding the collection of these samples can be found in Friedlaender et al. (2008).

AET and ATI: All aspects of this study adhere to the Declaration of Helsinki. Approval for each study described below was obtained through the Commission for Indigenous Peoples (Philippines), the Duke University Investigational Review Board and the University of Pennsylvania Investigational Review Board for the National Geographic's Genographic Project.

Unrelated members of the indigenous Aeta and Ati tribal populations of the Philippines were recruited after informed consent was obtained. Genomic DNA was isolated from venous blood collected in EDTA tubes. The goal of the project was to perform vision and general health screening (height, weight, blood pressure, medical and ophthalmic history) and to determine major causes of vision loss among middle-aged to older members of the Aeta and Ati. Subjects were selected to exclude known 1st or 2nd degree relatives. Assistance was provided by each village leader or "*kapitan*" in accordance with local custom and approval of the Commission for Indigenous Peoples (Philippines). Social workers contacted the leader (*kapitan*) of each village (*barangay)* to assist with the conduct of the study. The *kapitans* were asked to discuss the nature of the study, risks and benefits and, for willing participants, to randomly select unrelated couples and single family members from unrelated families for the screening without regard to visual status.

KEN, KIN, SNS, SNC, SNB and TEM: Written consent was obtained from each participant and this study was approved by the IRB committees of Penn State University (29269EP) and Universiti Kebangsaan Malaysia (UKM 1.5.3.5/244/FST-001-2010). We proceeded with permission from Jabatan Hal Ehwal Orang Asli, Malaysia (JHEOA) (PP.30.032 Jld 15(16)).

Participants from among the Orang Asli populations were recruited with the help of JHEOA and the Malaysian Ministry of Health's district health clinics at participating Orang Asli villages in 2010. Recruitment took place during the monthly health drive at

each village. Place of birth, ancestry of parents and grandparents, and number of siblings were obtained by interview.

5mL of each participant's blood was collected and mixed with an equal amount of storage buffer pH8, containing 100mM Tris HCl, ethylenediaminetetraacetic (EDTA) acid (100mM) and 2% sodium dodecyl sulfate. DNA from blood was extracted using phenol/ chloroform or the Qiagen DNA Blood kits [#51106 & 51185]. DNA integrity was checked by agarose gel electrophoresis and the quantity determined using a Qubit fluorometer.

IRU (GA000632 and GA00633) and SZH: Blood samples were collected from a self-declared healthy male and female from the Irula group (IRU) from Tamil Nadu, India.

For SZH, samples from two South Indian families affected with inherited retinal degeneration were collected based on clinical evaluations. In the first family, blood was collected from the two affected and two unaffected persons. In the second family, blood was drawn from the two affected and three unaffected persons.

The institutional ethics committee of SRM University, Kattankulathur, India, approved both studies.

JPN (GA001480 – GA001510): The Japanese samples used in this study were part of the THC (Tokyo Healthy Controls) who reside in the Tokyo area. All samples were de-identified of personal identifying information. Informed consents were obtained from all study subjects and the study protocols were approved by the IRB (G2583) of the Graduate school of Medicine, University of Tokyo.

GUJ, RAJ, BRA, SND, BRU, PAT, HAZ: DNA samples were collected by Syed Qasim Mehdi (deceased) with IRB approval from the University of Karachi, Pakistan.

JAR, ONG, AGH, DHR, DOR, MUR, ABM, BAG, BIR, BHM, HKR, KAM, LOD, MUN, ORN, TNT, WBB, CHM, KHA, SRB, CHK, JAM, MNP, MOG, TTO, GAU, HLB, RTH, CHN, IYA, IYE, KYD, KNR, LAM, PNY, TOD, KTA, MHR, NAB, SOB, NIC: DNA samples were collected by the National Institute of Biomedical Genomics (India) by Partha Majumder. Approximate sampling location and IRB approval information are given below.

Table S1.1

Populations	Code	Approximate Sampling Location	IRB Approval Obtained from
Abujmaria	ABM	Raipur, Chattisgarh	Pandit Ravishankar Shukla University, Dept of Anthropology, Raipur
Agharia	AGH	Sundergarh, Orissa	Indian Statistical Institute, Kolkata and Regional Medical Research Centre, Bhubaneswar
Bagdi	BAG	Medinipur, West Bengal	Indian Statistical Institute, Kolkata
Birhor	BIR	Chaibasa, Jharkhand	Indian Statistical Institute, Kolkata
BisonHornMaria	BHM	Raipur, Chattisgarh	Pandit Ravishankar Shukla University, Dept of Anthropology, Raipur
Chakma	CHK	Tripura	Tripura University
Chamar	CHM	Punjab & Haryana	Guru Nanak Dev University, Amritsar
Chenchu	CHN	Visakhapatnam, Andhra Pradesh	Madras University, Taramani, Chennai
Dhurwa	DHR	Chaibasa, Jharkhand	Indian Statistical Institute, Kolkata
Dorla	DOR	Raipur, Chattisgarh	Pandit Ravishankar Shukla University, Dept of Anthropology, Raipur
Gaud	GAU	Sundergarh, Orissa	Indian Statistical Institute, Kolkata and Regional Medical Research Centre, Bhubaneswar
Halba	HLB	Raipur, Chattisgarh	Pandit Ravishankar Shukla University, Dept of Anthropology, Raipur
Hill Korwa	HKR	Chaibasa, Jharkhand	Indian Statistical Institute, Kolkata
Iyengar	IYA	Chennai, Tamilnadu	Madras University, Taramani, Chennai

Iyer	IYE	Chennai, Tamilnadu	Madras University, Taramani, Chennai
Jamatia	JAM	Tripura	Tripura University
Jarawa	JAR	Andaman & Nicobar Islands	Regional Medical Research Centre, Port Blair
Kamar	KAM	Raipur, Chattisgarh	Pandit Ravishankar Shukla University, Dept of Anthropology, Raipur
Koya Dora	KYD	Andhra Pradesh	Madras University, Taramani, Chennai
Khatri	KHA	Amritsar	Guru Nanak Dev University, Amritsar
Konda Dora	KHD	Visakhapatnam, Andhra Pradesh	Madras University, Taramani, Chennai
Konda Reddy	KNR	Andhra Pradesh	Madras University, Taramani, Chennai
Kota	KTA	Nilgiri Hills	Bharathiar University, Dept. of Environmental Sciences, Coimbatore, Tamilnadu
Lambada	LAM	Nilgiri Hills	Bharathiar University, Dept. of Environmental Sciences, Coimbatore, Tamilnadu
Lodha	LOD	Medinipur, West Bengal	Indian Statistical Institute, Kolkata
Mahar	MHR	Punjab & Haryana	Indian Statistical Institute, Kolkata
Manipuri	MNP	Manipur	Tripura University
Mog	MOG	Tripura	Tripura University
Munda	MUN	Bihar	Indian Statistical Institute, Kolkata
Muria	MUR	Raipur, Chattisgarh	Pandit Ravishankar Shukla University, Dept of Anthropology, Raipur
Nav Buddha	NAB	Maharashtra	B.J. Medical College, Pune

Nicobarese	NIC	Andaman & Nicobar Islands	Regional Medical Research Centre, Port Blair
Onge	ONG	Andaman & Nicobar Islands	Regional Medical Research Centre, Port Blair
Oraon	ORN	Bihar	Indian Statistical Institute, Kolkata
Paniya	PNY	Kerala	Bharathiar University, Dept. of Environmental Sciences, Coimbatore, Tamilnadu
RanaTharu	RTH	Uttar Pradesh	Indian Statistical Institute, Kolkata
Saryupari Brahmin	SRB	Raipur, Chattisgarh	Pandit Ravishankar Shukla University, Dept of Anthropology, Raipur
Sourastra Brahmin	SOB	Maharashtra	B.J. Medical College, Pune
Tanti	TNT	Kolkata, West Bengal	Indian Statistical Institute, Kolkata
Toda	TOD	Nilgiri Hills	Bharathiar University, Dept. of Environmental Sciences, Coimbatore, Tamilnadu
Toto	TTO	Jalpaiguri, West Bengal	Indian Statistical Institute, Kolkata
West Bengal Brahmin	WBB	Kolkata, West Bengal	Indian Statistical Institute, Kolkata

Reference

Friedlaender JS, Friedlaender FR, et al. (2008). The genetic structure of Pacific islanders. PLoS Genet 4: e19.

Supplementary note S2 – Mapping, filtering and variant calling

Aakrosh Ratan
Center for Public Health Genomics
University of Virginia School of Medicine, Charlottesville, Virginia 22908

author responsible for this section: Aakrosh Ratan - ar7jq@virginia.edu

Summary:

To minimize confounding effects that can arise when data are processed differently(1) we limited samples to those sequenced using the Illumina sequencing platform, and used a uniform pipeline for mapping, filtering and variant calling. We focused on a set of 1,739 genomes (1,236 new and 503 previously generated genomic sequences) that satisfied strict quality-control filters. After excluding first-degree relative pairs the analyses described primarily centered on a subset of 1,667 individuals. We observed a total of 63,178,770 high-quality SNPs and 3,849,750 indels in the genomes from these samples.

To aid in comparisons across different continents and different data sets, our high-coverage sequencing included 119 individuals who were previously sequenced at low (~7X) coverage as part of the 1000 Genomes Project(2). We tabulated the concordance rate between our variant calls and those of the 1000 Genomes Project for these duplicate samples. Overall, the variant concordance rate is 99.63% (see Methods). This suggests that despite the low coverage of the 1000 Genomes Project samples, the imputed genotypes from these genomes are, on average, highly accurate. As expected, the average variant discordance rates increase with decreasing minor allele frequency (MAF), ranging from 0.18% for SNPs with 1000 Genomes Project MAF > 0.05 to 8.12% for SNPs with MAF ≤ 0.002 (**Supplementary Table S2b**). These discordance rates are generally lower than the comparable rates estimated by the 1000 Genomes Consortium(2). This may reflect in part

the increased genotype discordance caused by platform-specific biases between Illumina and Complete Genomics sequencing(3).

Alignment of short reads

Software used

- BWA version 0.7.13 (https://github.com/lh3/bwa)
- SAMBLASTER version 0.1.22 (https://github.com/GregoryFaust/samblaster)
- Sambamba version 0.6.1 (https://github.com/lomereiter/sambamba)
- BAMreport version 0.0.2 (https://github.com/aakrosh/BAMreport)
- verifyBamID version 1.1.3 (http://genome.sph.umich.edu/wiki/VerifyBamID)

Methods

We aligned the Illumina short-read sequences to the GRCh37+decoy reference sequence with BWA mem using the default parameters. Putative PCR duplicates were flagged using SAMBLASTER, which was also used to add MC and MQ tags to all the output paired-end alignments, and separate the split-reads, the discordant pairs, and the unmapped sequences from the resulting output. The SAM outputs were converted to BAM format, and sorted by chromosomal coordinates using Sambamba. All BAM files for the same samples were merged using Sambamba, and BAMreport was then used to generate the alignment statistics and metrics.

The sex of the samples was inferred from the coverage of the autosomes and the sex chromosomes and confirmed using the submitted metadata with the samples. All samples that had an average coverage < 20, or where we found a difference in the inferred and reported sex were ignore from further analysis. verifyBamID was run to identify contaminations using a chip-free mode, and samples where swaps or contamination was identified were ignored from subsequent analyses. Contamination level 3% was used as a cut-off, and this left us with 1,739 samples that were used for all subsequent analyses.

Figure S2.1: Scatterplot showing the generated sequences vs. the aligned non-duplicate bases for 1,739 samples in the study. The horizontal line corresponds to a coverage of 20-fold. All samples included in this study have an average coverage greater than 20

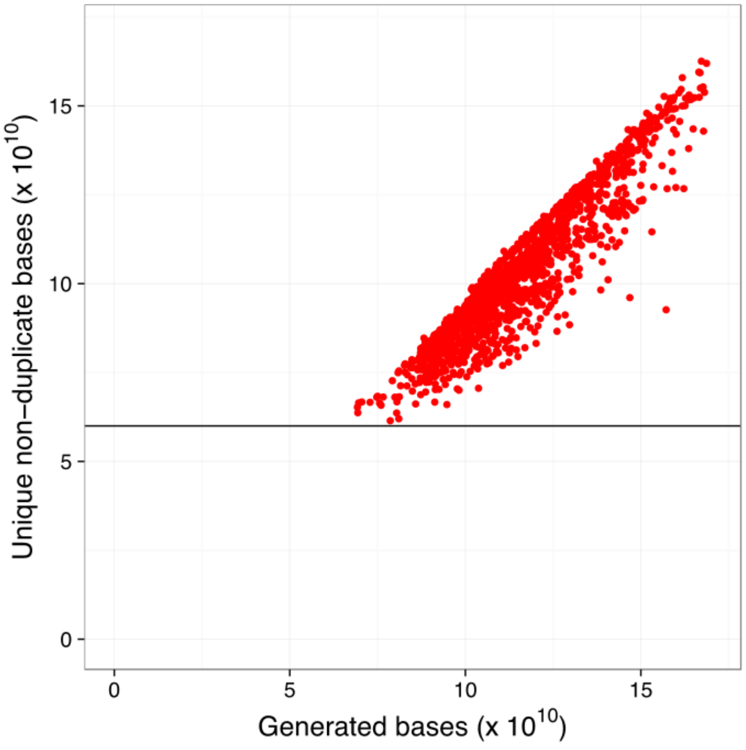

Identification of SNVs and small indels

Software used

- GATK version 3.5 (https://software.broadinstitute.org/gatk/)
- vcfanno version 0.1.0-dev (https://github.com/brentp/vcfanno)
- htslib version 1.3.1-64-g74bcfd7 (https://github.com/samtools/htslib)
- vcftools version 0.1.14 (https://vcftools.github.io/index.html)
- plink version 1.90b3.40 (http://zzz.bwh.harvard.edu/plink/)
- king version 1.4 (http://people.virginia.edu/~wc9c/KING/)
- rtg-tools version 3.7 (https://github.com/RealTimeGenomics/rtg-tools)

Methods

We identified the single nucleotide substitutions and small indel variants in the 1,739 samples using the the reference model (gVCF-based) workflow for joint analysis using

GATK. Variants were called individually in each sample using the HaplotypeCaller in "-ERC GVCF" mode to produce a record of genotype likelihoods and annotations at each site in the genome. A gVCF file was created for every sample, and a subsequent joint genotyping analyses of all the gVCFs was done to identify the variants in the cohort. We followed the GATK recommended best practices for variant recalibration to create a final VCF file and recalibrated the variants to select 99% of the true sites from the training set for VQSR. The VCF files were zipped using bgzip and indexed using tabix.

The Genome in a Bottle Consortium (GIAB) is a public-private-academic consortium with the overarching goal to enable translation of whole-genome human sequencing to clinical practice. As part of their efforts they have released a set of high confidence variants calls from some samples including NA12878 and NA24385 (ftp://ftp-trace.ncbi.nlm.nih.gov/giab/ftp/release/), both which are included in this study, albeit at lower coverage. We used rtg-tools to calculate the precision and sensitivity of our call-set using *vcfeval*, for these two samples. The table below shows precision and sensitivity for both substitution and indel calls made using the GATK Haplotypecaller when the evaluation is restricted to the confident regions as reported by GIAB.

**Sample**	**Variants**	**Precision**	**Sensitivity**
NA12878	Substitutions	0.9999	0.9082
NA12878	Indels	0.9699	0.9034
NA24385	Substitutions	0.9999	0.9104
NA24385	Indels	0.9632	0.9060

Comparison of variants, especially complex variants including indels can be challenging. When we consider all locations including MNPs and indels with multiple alternate alleles, the precision and sensitivity for NA12878 indels is 0.9699 and 0.9034 respectively. Similarly, the values of precision and sensitivity for NA24385 are 0.9632 and 0.9060 when indels at all locations are considered.

In order to add annotations to the VCF file, we downloaded the ancestral sequences for Homo sapiens (GRCh37) from ftp://ftp.ensembl.org/pub/release-72/fasta/ancestral_alleles. We converted the ancestral sequences from fasta to VCF format using an in-house python script. We downloaded the genotypes for the sequenced Neanderthal and Denisovan genomes from http://cdna.eva.mpg.de/neandertal/altai/AltaiNeandertal/VCF/ and http://cdna.eva.mpg.de/neandertal/altai/Denisovan/ respectively. We used vcfanno to add the information about the ancestral allele, and the genotypes of the two hominids to the VCF file.

Several of the analyses require selection of unrelated samples. In order to identify the related samples, we first used vcftools and plink to convert the files into required formats, and then used KING to infer relationships among the sequenced samples. KING accurately identified all but one of the first-order relationships between the samples (as evident from the metadata submitted with the samples) when the estimated kinship coefficient was restricted to [0.177, 0.354]. GA000638 and GA000639 were reported as being first-degree relatives in the supplied metadata, and this was confirmed using PLINK which found that the PI-HAT values for the siblings was a little lower than those for other siblings.

Additionally, the final annotated VCF files were converted into a simple tabular format with the following columns using an in-house python script to facilitate some analyses:

1. chromosome
2. 1-based position of the substitution
3. reference allele
4. alternate allele
5. ancestral allele
6. Neanderthal genotype
7. Denisovan genotype
8. Genotype of samples in order

We also generated another tabular file in the same format that was restricted to the locations in the 1000 Genomes Strict Accessibility Mask (http://www.internationalgenome.org/announcements/genome-accessibility-information-now-available-1000-genomes-browser-2012-09-06/)

Figure S2.2: Substitutions identified in the sampled populations.

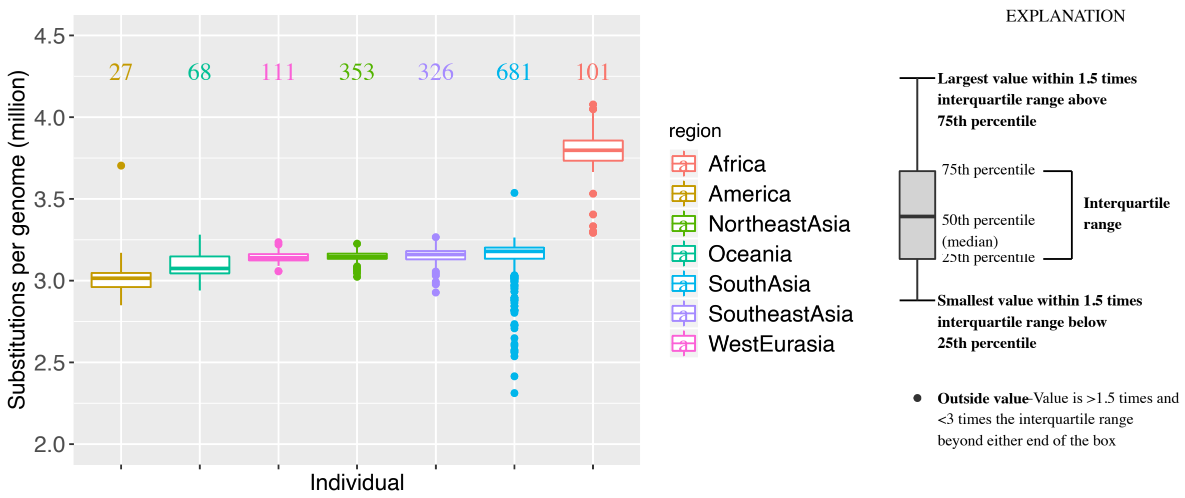

Figure S2.3: Singletons identified as fraction of all substitution calls made for the individual in the sampled populations.

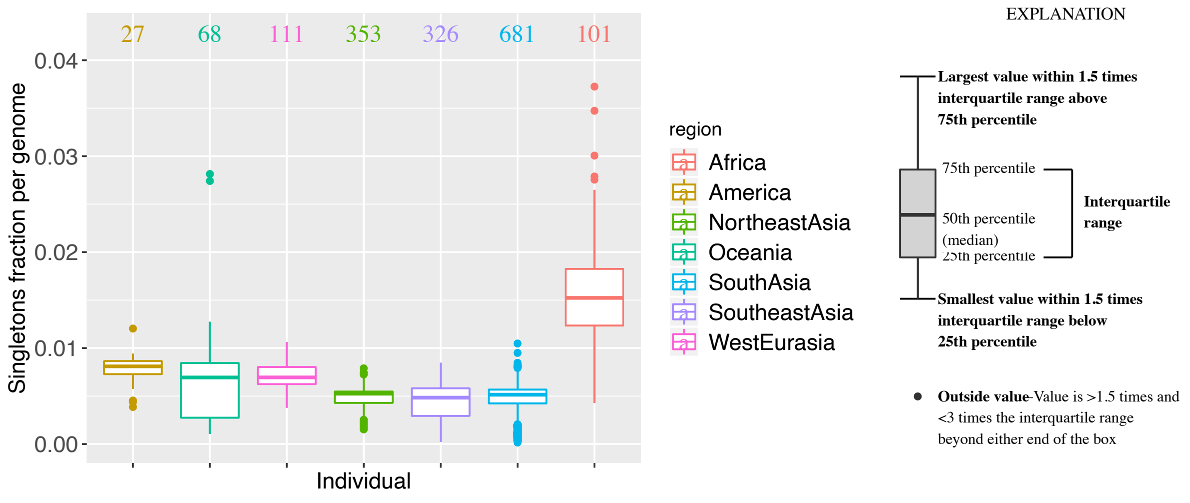

Figure S2.4: Most of the variation identified in the genomes from the sampled populations is rare variation.

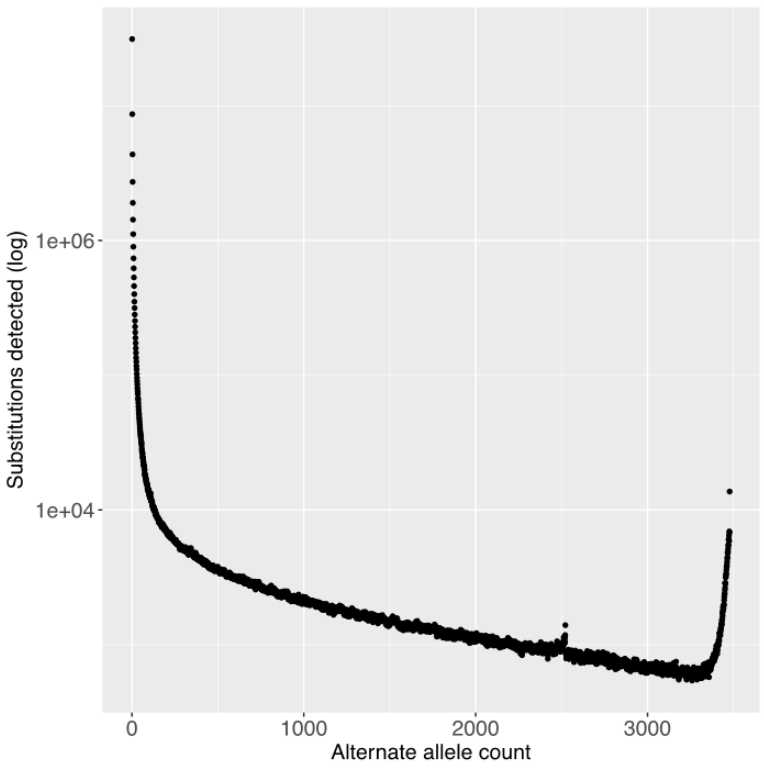

We ran “CollectVariantCallingMetrics” from the Picard toolkit on both the SNP and indel calls to evaluate the quality of our variant callset. The results from the run are included in Supplementary Tables S2b and S2c. We present a few of the metrics as boxplots stratified based on the Continental region.

Figure S2. 5: The ratio of heterozygous to homozygous SNPs identified for the individual in the sampled populations.

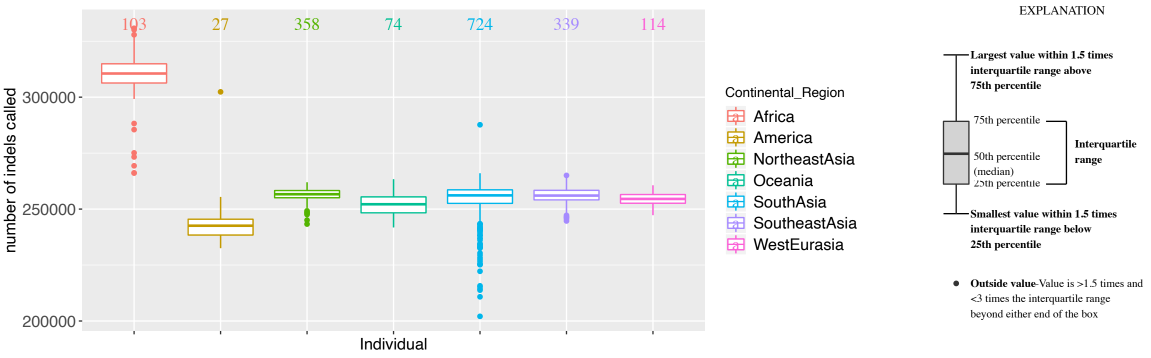

Figure S2. 6: The percentage of identified SNPs that are also found in dbSNP version 138 for the individual in the sampled populations.

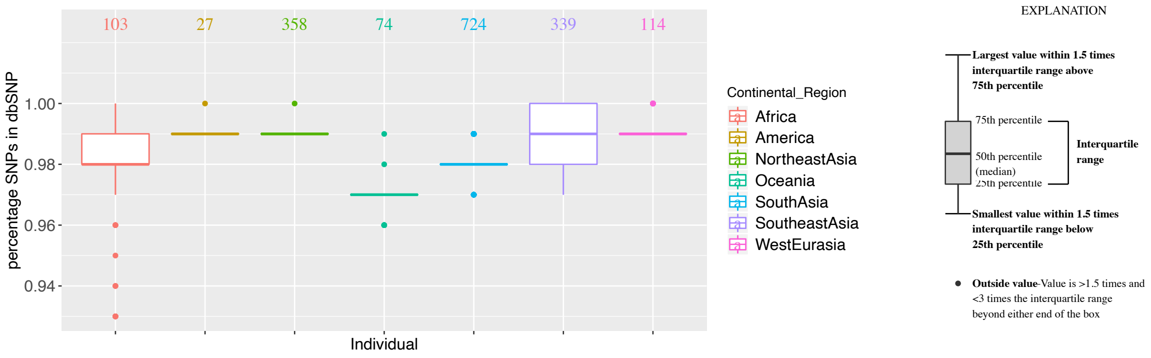

Figure S2. 7: The number of indels identified for the individual in the sampled populations.

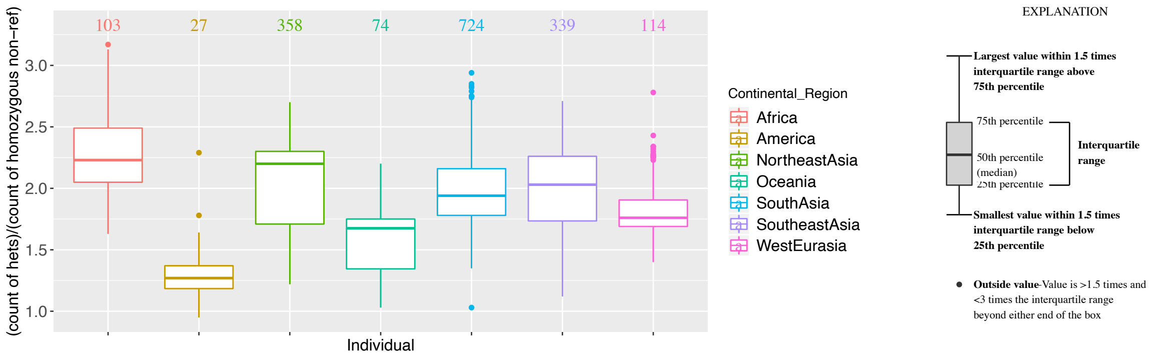

Figure S2. 8: The ratio of heterozygous to homozygous indels identified for the individual in the sampled populations.

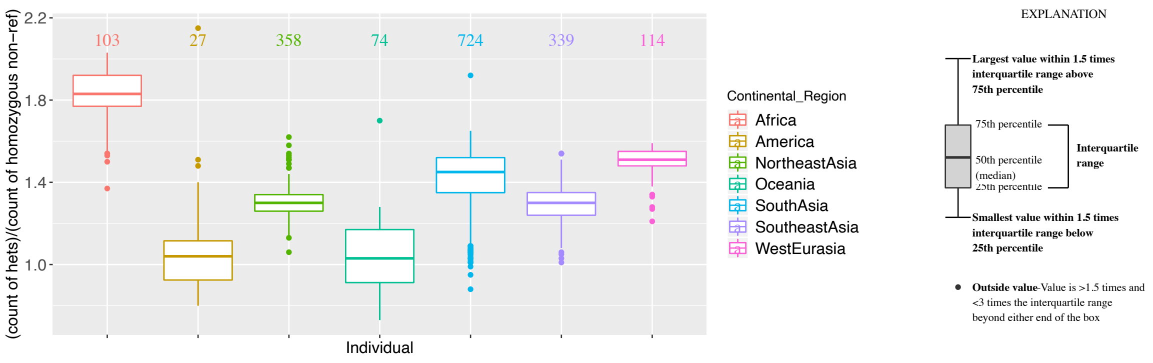

Figure S2. 9: The percentage of identified indels that are also found in dbSNP version 138 for the individual in the sampled populations.

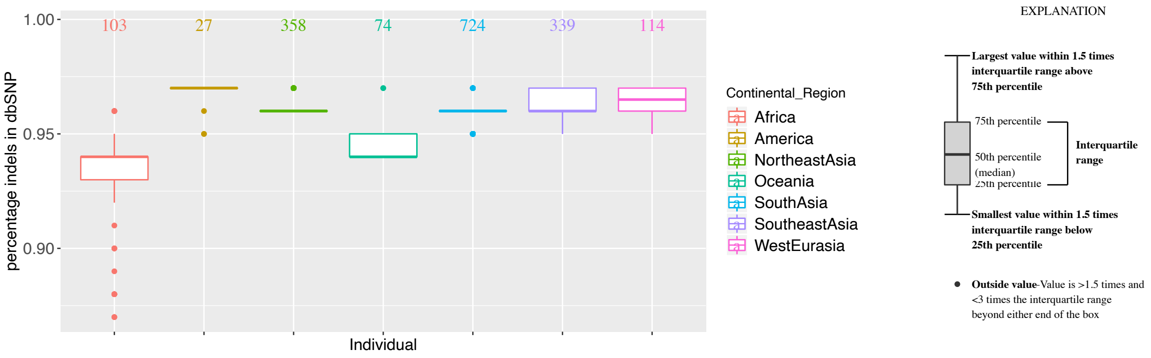

Supplementary note 3 – Phasing and MSMC

Hie Lim Kim[1,2] and Jennifer Tom[3]

[1]The Asian School of the Environment, Nanyang Technological University, Singapore 637459. [2]Singapore Centre for Environmental Life Sciences Engineering, Nanyang Technological University, Singapore 637551. [3]Department of Bioinformatics and Computational Biology, Genentech, Inc., South San Francisco, California 94080, USA.

authors responsible for this section:
Hie Lim Kim - HLKIM@ntu.edu.sg [MSMC analysis]
Jennifer Tom - tom.jennifer@gene.com [Phasing]

Summary

Our dataset contains enough samples from each population to reconstruct population size changes and split times using by the multiple sequentially Markovian coalescent (MSMC) (Schiffels and Durbin, 2014). The results could help explain the earliest demographic events in Asia subsequent to the out of Africa migration. We found that the most ancient lineages in Southeast Asia and Oceania are the Melanesians and Negritos, who show substructure from ~40 kya. Within the Negrito groups, there is evidence of separation around 20 – 30 kya. The large estimated population sizes for Philippine and Malay Negrito groups from 10 – 20 kya suggests they had prosperous settlements in the region. In contrast the Andamanese show reduced population size estimates since their split from other Negrito groups, possibly due to their geographic isolation (Figure S4.2C). The separation among Indian groups gradually occurred from between 10 – 24 kya, which is consistent with previous studies (Basu et al. 2016). The split of Northeast Asians with Austronesians is estimated at 6 – 23 kya, earlier than the estimated split time between Northeast Asians and Mongolians at 12 – 16 kya. Therefore, Koreans, Japanese and Mongolians likely share recent common ancestry. **(Note that all of these time estimates do not reflect the substantial uncertainty in *de novo* mutation rates and generation times.)**

Methods

Phasing using Shapeit

We phased the whole genomes using Shapeit v2 (Delaneau et al, 2012). Starting from the final SNP calls, we filtered SNPs based on the call rate (missing rate) and minor allele frequency to increase the accuracy of phasing inference. Among the 1667 unrelated individuals, we removed those SNPs having > 1% missing genotypes or < 0.1% minor allele frequency.

Shapeit allows incorporating known phases extracted from BAM files using the tool extractPIRs (Delaneau et al, 2013) to improve the phasing quality. We used this information using '–assemble' mode.

The phasing was inferred in two steps depending on the groups which were classified based on the ADMIXTURE result. We identified 12 reference groups (**Figure S3.1, Table S3.1**) and phased each group independently without a reference panel (since there are no reference data available for the indigenous groups). Our reasoning was that we have a large enough sample size for most groups, and the groups having a small number of samples are very homogeneous. Secondly, for the highly admixed populations, we determined the putative ancestral groups and phased the admixed populations with the appropriate reference panels (**Table S3.2**). The Admixed group 11 contains only 6 samples and was phased without MCMC iterations, as suggested by the authors of Shapeit.

In the group classification, 13 samples were identified as outliers and were not included in the phasing, therefore, in total 1654 individuals were phased.

Table S3.1. Reference groups **S3.2**. Admixed groups

Group	No. of sample
Africa	77
Andaman	21
Austronesian	28
Europe	107
Indo-European	339
Mongol	111
Philippine	26
Northeast Asia	198
Oceania	66
Tribe Indian	160
American	21
Malay Negrito	24
Total	1178

Group	No. of sampl	Reference panel	phasing option
1	24	Africa, Europe	mcmc
2	73	Indo-European, Tribe Indian	mcmc
3	20	Europe, Mongol, Northeast Asia	mcmc
4	19	Tribe Indian, Northeast Asia	mcmc
5	46	Tribe Indian, Northeast Asia, Austronesian	mcmc
6	93	Malay Negrito, Austronesian	mcmc
7	22	Philippine Negrito, Austronesian, Oceania	mcmc
8	41	Austronesian, Oceania, Malay Negrito	mcmc
9	74	Austronesian, Northeast Asia, Malay Negrito	mcmc
10	18	Mongol, Northeast Asia, Europe, America	mcmc
11	6	Northeast Asia, Austronesian, Mongol	no-
12	40	Austronesian, Northeast Asia, Indo-European, Malay Negrito	mcmc
Total	476		

Phasing using Eagle

We also phased the data using the Eagle2 algorithm (Loh et al. 2016), version 2.3. We jointly phased all 1,739 samples without a reference panel. The phasing relied on the default genetic map included in the Eagle tables (genetic_map_hg19_withX.txt.gz) and the multithreading option (--numThreads 16).

MSMC

We used two different phased genome datasets as described above. Chromosome 6 was excluded in the analysis due to possible phasing errors in the HLA region. We generated input files using the tool, generate_multihetsep.py, downloaded from https://github.com/stschiff/msmc-tools. We used four haplotypes (two individual genomes) for estimating population size changes in a population and eight haplotypes (two genomes from each of a pair of populations) for population split time estimation, using MSMC2 (Schiffels and Durbin, 2014). For scaling of the time point and effective population size, we assumed a mutation rate of μ=1.25×10^{-8} per site per generation and a generation time of 29 years, as in previous studies (Schiffels and Durbin, 2014; Mallick et al, 2016).

Results

Population size changes

For each population, 4 – 5 pairs of genomes (4 haplotypes) were selected to estimate population size changes. Highly admixed individuals and closely-related pairs based on the IBD (PH_HAT>0.3) were not included in the analysis. After masking low-quality sequenced regions using SAMtools for generating input files, we also excluded those genomes having a relatively small number of SNPs. Populations were selected from the reference groups categorized for phasing (see above).

Indigenous groups such as Baining, Negritos (Aeta and Kensiu) and South Asian Adivasi (tribal groups such as the Paniya) increased in size around 10-20 kya. Native Americans and Andamanese have decreased in their population size.

Figure S3.2. Population size changes for specific populations as estimated using MSMC.

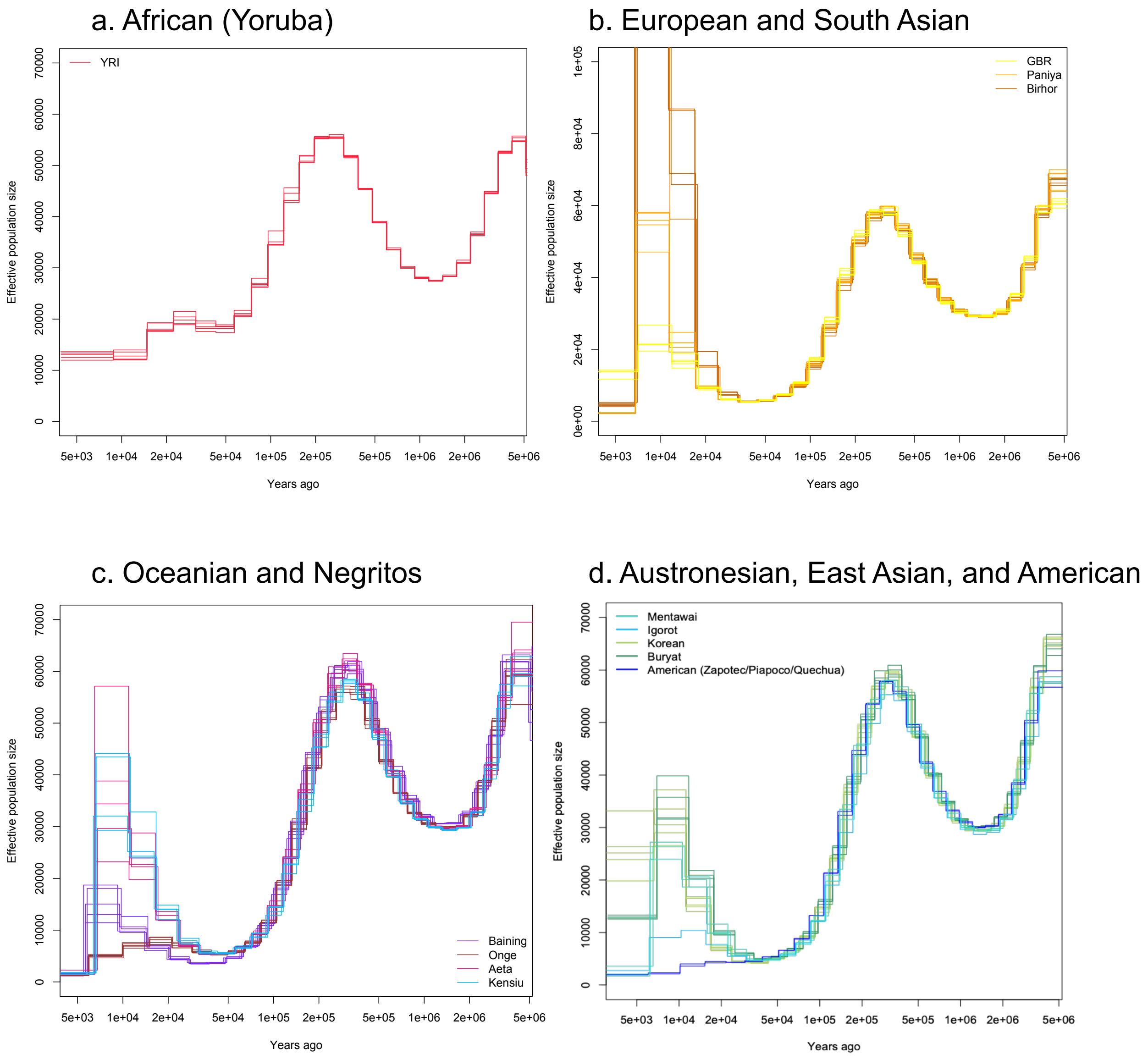

(a) Yoruba was chosen as the ancestral population of non-Africans.
(b) GBR (British) as the European ancestry, Birhor as the South Asian group without European admixture, Paniya as the least admixed South Asian group, were selected.
(c) Baining is the non-admixed Melanesian group according to the admixture analysis. Onge is one of two Andamanese. Aeta and Kensiu were selected as Philippine and Malaysian Negritos respectively.

(d) Mentawai and Igorot are the most non-admixed Austronesians. We have only two Igorot genomes, therefore, Mentawai is also included as Austronesians. Koreans as Northeast Asians and Buryat as Mongols were used for the analysis. Two genomes from each of three different Native American ethnic groups, Quechua, Zapotec, and Piapoco, were selected.

Population split times (relative cross-coalescence rates)
The individual genomes used for the population split estimates were selected from the genomes used for the population size changes.

Figure S3.3. Cross-coalescence rates for selected pairs of populations.

a. South Asia b. Southeast Asia and Oceania

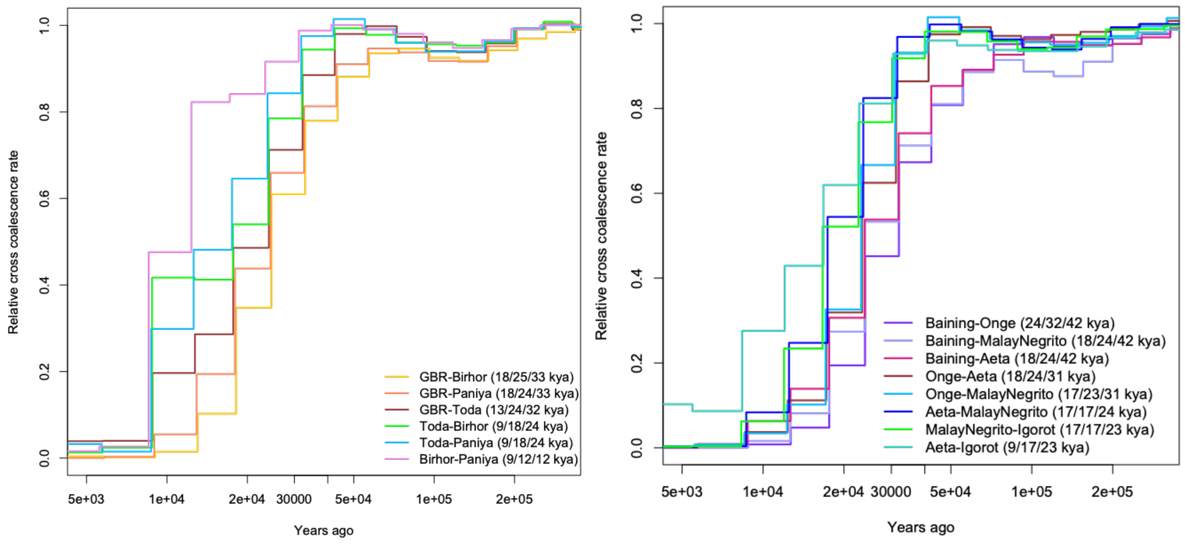

c. North Asia

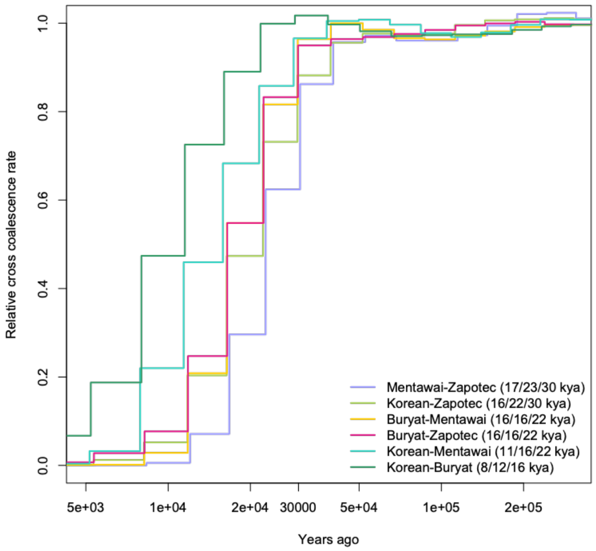

(a) South Asian populations were split with European ancestors at 24~33 kya and structured within South Asia at 12~24 kya.

(b) Melanesians and Negrito populations in Oceania and Southeast Asia are deeply diverged. The deepest split between Papuans (Baining) and Negrito ancestors is 32~42 kya. This indicates their common ancestors reached the Southeast Asia area earlier than 40 kya and already were structured maybe by geographic distance. Among the Negritos, Andaman (Onge) split first at 23~31 kya, while Philippine (Aeta) and Malaysia Negritos (Kensiu) as well as Austronesians (Igorot) split at 17~24 kya. Around 10~20 kya, the sea level in this region most dramatically has risen and Philippine was separated from the land. Igorot living in Philippine might have contacted with Philippine Negrito (Aeta) since their split is slower than the split with Malay Negrito. We applied this approach to the two Australia samples retrieved from the Simons Genome Diversity Project dataset (Mallick et al. 2016) to reconstruct even the deeper history. However, the two samples showed not good enough sequencing quality and possible admixture, therefore we did not include the samples in this analysis.

(c) The split between Native Americans and the common ancestors of Austronesians (Mentawai) and Northeast Asians (Korean) was about 22~30 kya. Austronesians were split with Mongols (Buryat) at 16~22 kya. Northeast Asians are split with Mongols 12~16 kya.

Early split with Baining

Figure S3.4. More MSMC cross-coalescence plots

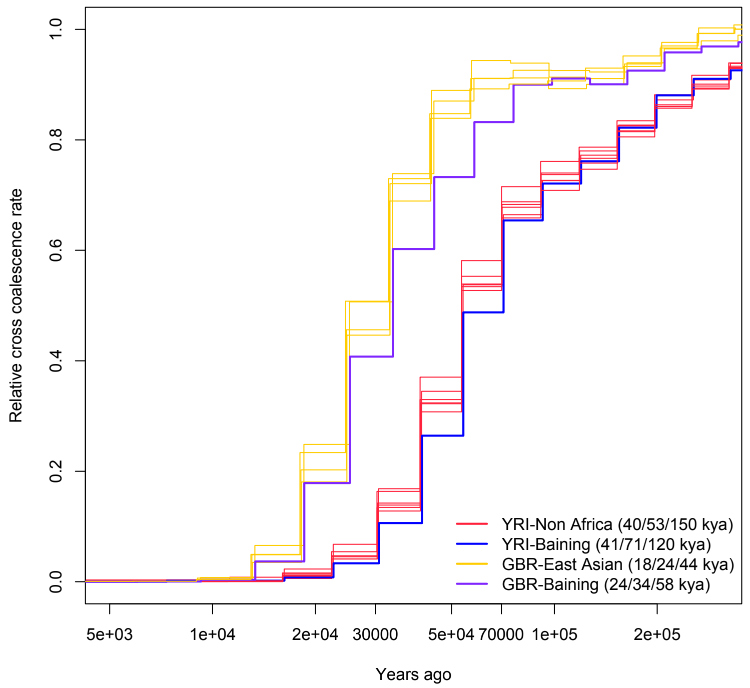

The splits of all pairs with the African representative population (Yoruba) showed very similar patterns, which suggests a single event of the out of Africa migration. However, the split with Baining (non-admixed Melanesians living in New Britain, Papua New Guinea) is apparently earlier (71 kya, 50%) than all other splits (53 kya, 50%), similar to the results of Pagani et al. (2016). The splits of Southeast and East Asian populations with Europeans (GBR) occurred consistently around 24~44 kya, while the split with Baining is 10 kya earlier, at 34~58 kya. This early split is the same with other pairs of Baining genomes or another dataset phased by different method. This could be a result of archaic admixture (e.g., from Denisovans) into the Baining but not into other non-African groups.

Comparison between different phasing algorithms

For the population size change, the estimates are very similar except for the recent history up to 10,000 years ago for some populations, between the two different phased genome datasets. Since the Eagle dataset was phased with the entire dataset as a single dataset/group, the haplotype estimation might pick up the phasing from non-related populations, and showed high peaks of population size in the recent history. For example, even the YRI genomes showed the high peak even they don't have any admixture signal in other analyses, such as ADMIXTURE and PCA.

The population split estimates are slightly shifted, about 10 kya, to recent for the Shapeit dataset (a) compared to the Eagle dataset (b). Figure 2D and the other time estimates in this supplement are from the Shapeit dataset.

Figure S3.5. The population split estimates - Shapeit dataset (a), Eagle dataset (b) and Shapeit dataset compared to the Eagle dataset (c).

a. Shapeit

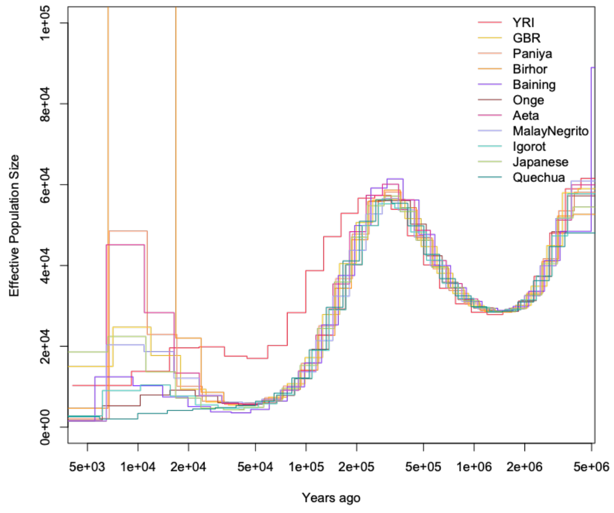

b. Eagle

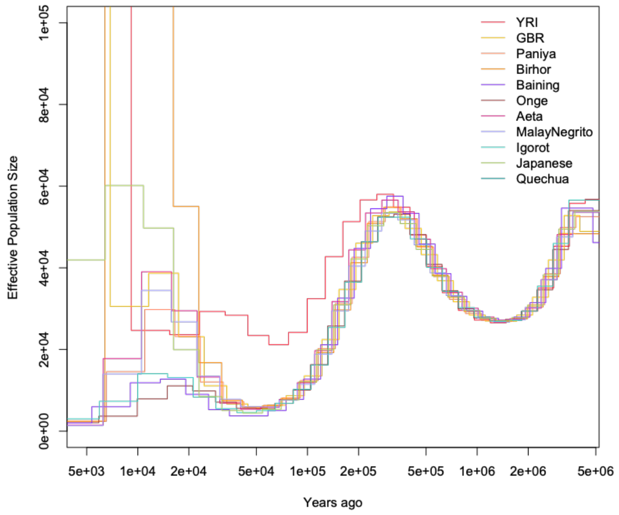

c. Population split estimation between the two datasets

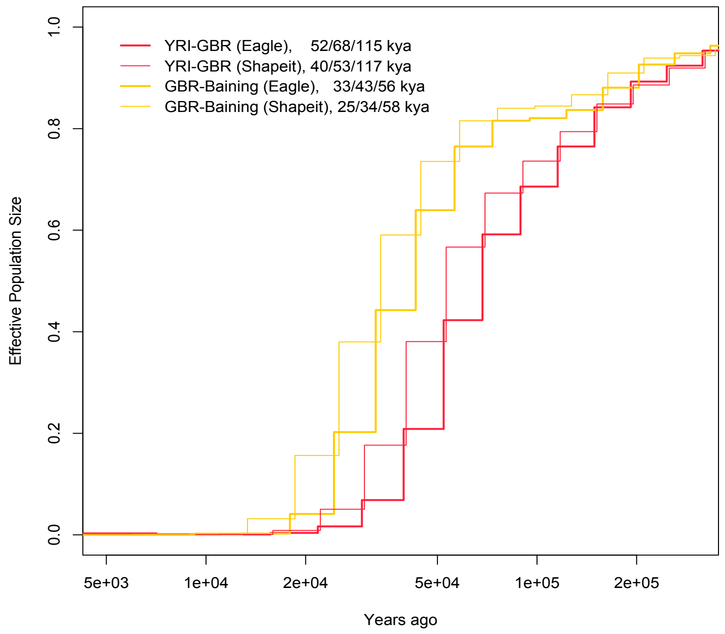

References

Basu A, Sarkar-Roy N, and Majumder PP, Genomic reconstruction of the history of extant populations of India reveals five distinct ancestral components and a complex structure, PNAS USA, 113:1594-1599, 2016.

Delaneau O, Marchini J, and Zagury JF, A linear complexity phasing method for thousands of genomes, Nat Methods, 9:179-181, 2012.

Delaneau O, Howie B, Cox A, Zagury JF, and Marchini J, Haplotype estimation using sequence reads. American Journal of Human Genetics, 93:687-696, 2013.

Loh PR, Palamara PF and Price AL, Fast and accurate long-range phasing in a UK Biobank cohort. Nature Genetics, 48:811-816, 2016.

Mallick S, Li H, Lipson M, Mathieson I, Gymrek M, et al, The Simons Genome Diversity Project: 300 genomes from 142 diverse populations, Nature, 538:201-206, 2016.

Pagani L, Lawson DJ, Jagoda E, Morseburg A, et al, Genomic analyses inform on migration events during the peopling of Eurasia, Nature, 538:238-242, 2016.

Schiffels S and Durbin R, Inferring human population size and separation history from multiple genome sequences, Nat Genet, 46:919-925, 2014.

Supplementary note 4 – Population structure and admixture

Elena Gusareva[1] and Hie Lim Kim[1,2]

[1]Singapore Centre for Environmental Life Sciences Engineering, Nanyang Technological University, Singapore 637551. [2]The Asian School of the Environment, Nanyang Technological University, Singapore 637459.

author responsible for this section:
Hie Lim Kim - Hie Lim Kim - HLKIM@ntu.edu.sg

Results

Admixture

We examined population structure and identified ancestral components with an autosomal marker dataset (1,089,227 SNPs) of 1,667 unrelated individuals, using Admixture v.1.3.0 (Alexander et al, 2009) (**Figure S4.1a**). According to CV error (**Figure S4.1b**), K=14 is the most well-represented population structure for this dataset, though the CV error values are almost same for K=10 – 14.

The results show the high diversity and complicated population structure of Asian populations. The diversity is shaped by several distinct ancestries, such as Negrito groups and Melanesians in Southeast Asia. Most ethnic groups in South and Southeast Asia are composed of several ancestries, indicating complicated admixture events during a long history in the region. Below, we assume the ancestral populations identified through the Admixture plots to have originated in the location(s) where putatively unadmixed individuals currently reside (e.g., ancestry in the light blue population is colloquially called Austronesian ancestry).

A. South Asians

The populations contain mostly European and most of the Asian ancestries. The European ancestry is found mostly in Northern Indian groups and Central Asians, while many tribal groups living in India contain Southeast Asia ancestry. Some ethnic groups living in Northeast India contain also the Northeast Asian ancestry. The Andaman Negrito groups are distinctive compared to the current South Asian populations. The Toda population is a separate ancestry component from K=10. This is broadly consistent with the F_{ST} results (Figure S5.2) and PCA analyses (see below).

B. Southeast Asians

The Mainland Southeast populations are admixed groups of Austronesians and Negritos, while the Island Southeast populations are admixed between Austronesians and Melanesians. This is explained by recent and local contacts between earlier migrants (Negritos and Melanesians) and recent migrants (Austronesians), 2,000~6,000 years ago. Even though the major populations are mostly Austronesian-language speakers in the area, the earlier settlers, currently minor groups, have largely contributed to shape the populations genetically.

C. Northeast Asians

The Southeast Asian ancestries are one of the major ancestral components of Vietnamese and Southern Chinese. However, Northeast Asians (Koreans, Japanese, and Mongolians) do not contain these ancestries. Since a split in ancestries between Mongolians and Koreans/Japanese only occurs at K=11, it is likely that Koreans and Japanese are closely related to Mongolians.

Figure S4.1.

a. Admixture plot

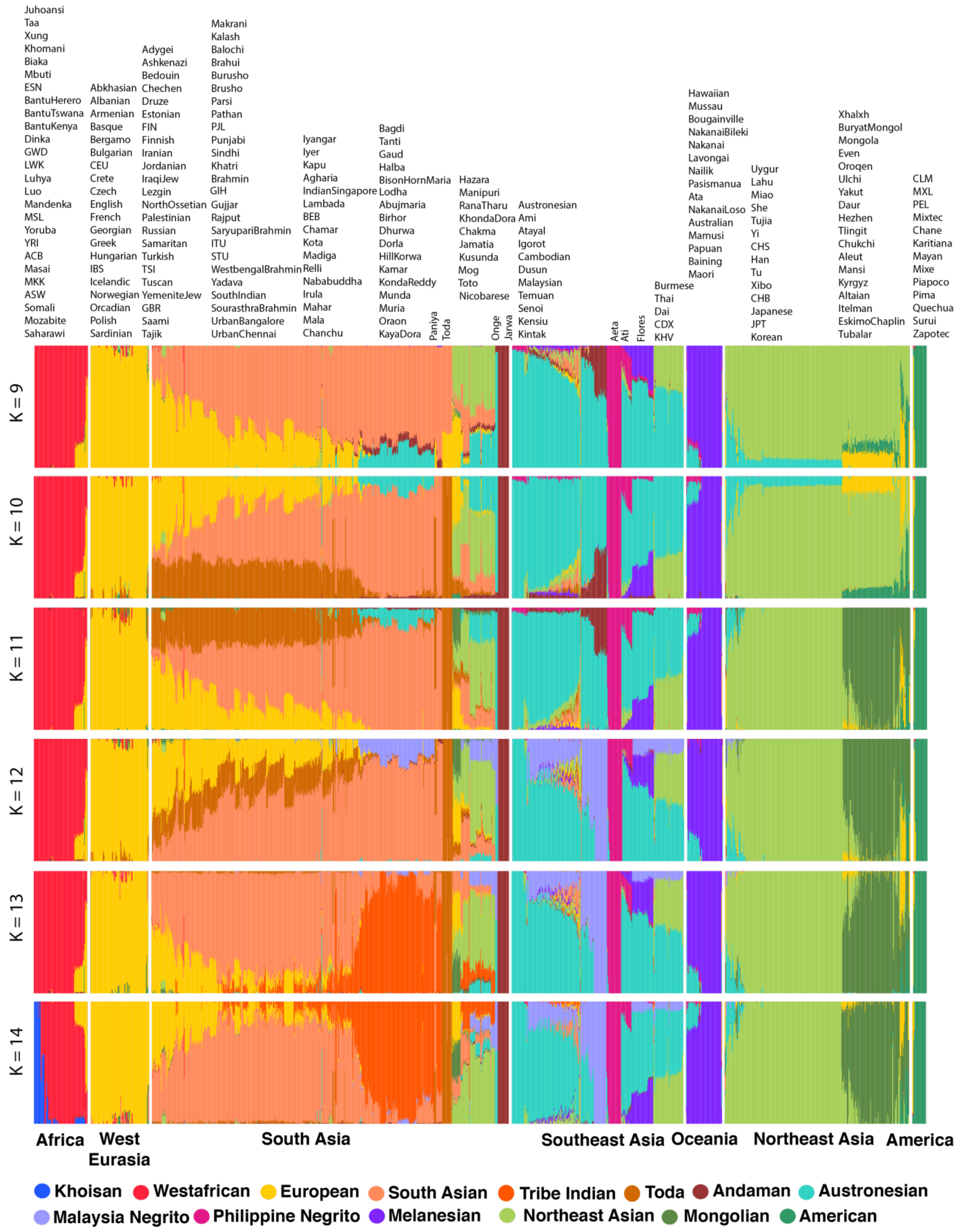

b. CV error plot

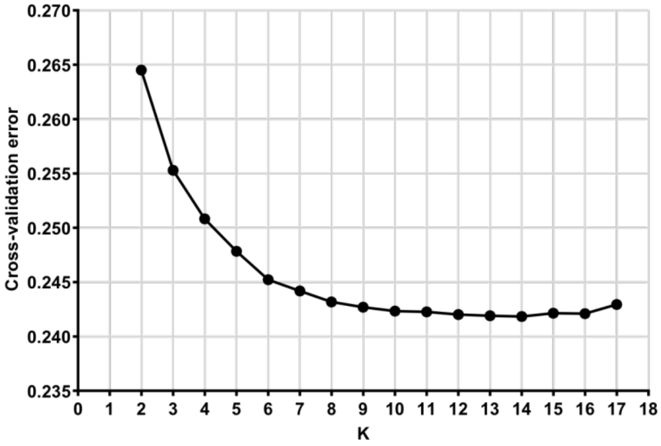

PCA

Another approach to examining population structure and genetic distance between populations is Principal component analysis (PCA). The same SNP dataset used for the Admixture analysis was applied to PCA using EIGENSTRAT v.6.1.4 (Price et al, 2006). The result for the entire dataset is in the main text (Figure 2A). Here we show the results for each population group.

Figure S4.2. a-d

NEA

Aleut n=2,Altaian n=1,Buryat n=87,CHB n=12,CHS n=1,Chukchi n=1,Daur n=1,EskimoChaplin n=1,EskimoNaukan n=2,EskimoSireniki n=2,Even n=3,Han n=7,Hezhen n=2,Itelman n=1,JPT n=2,Japanese n=33,Korean n=152,Kyrgyz n=2,Mansi n=2,Mongola n=3,Naxi n=2,Oroqen n=2,She n=2,Tlingit n=2,Tu n=2,Tubalar n=2,Tujia n=2,Ulchi n=2,Uygur n=2,Xhalxh n=12,Xibo n=2,Yakut n=2

OCE

Ata n=2,Australian n=2,Baining n=19,Bougainville n=2,Hawaiian n=1,Lavongai n=1,Mamusi n=2,Maori n=1,Mussau n=1,Nailik n=2,Nakanai n=7,Nakanai Bileki n=11,Nakanai Loso n=1,Papuan n=20,Pasismanua n=2

SAS

Abujmaria n=11,Agharia n=17,BEB n=2,Bagdi n=3,Balochi n=2,Birhor n=20,BisonHornMaria n=5,Brahmin n=2,Brahui n=11,Brusho n=8,Burusho n=2,Chakma n=11,Chamar n=6,Chanchu n=8,Dhurwa n=10,Dorla n=12,GIH n=1,Gaud n=9,Gujjar n=20,Halba n=6,Hazara n=17,HillKorwa n=10,ITU n=26,Indian n=38,Irula n=4,Iyangar n=6,Iyer n=13,Jamatia n=9,Jarwa n=12,Kalash n=2,Kamar n=11,Kapu n=2,KayaDora n=11,Khatri n=11,KhondaDora n=1,KondaReddy n=17,Kota n=8,Kusunda n=2,Lambada n=17,Lodha n=11,Madiga n=2,Mahar n=19,Makrani n=2,Mala n=2,Manipuri n=5,Mog n=20,Munda n=2,Muria n=10,Nababuddha n=4,Nicobarese n=6,Onge n=15,Oraon n=15,PJL n=3,Paniya n=11,Parsi n=1,Pathan n=17,Punjabi n=2,Rajput n=14,RanaTharu n=12,Relli n=2,STU n=9,SaryupariBrahmin n=14,Sindhi n=12,SourasthraBrahmin n=9,SouthIndian n=1,SouthIndian n=9,Tanti n=1,Toda n=20,Toto n=11,UrbanBangalore n=34,UrbanChennai n=34,WestbengalBrahmin n=10,Yadava n=2

SEA

Aeta n=29,Ami n=2,Atayal n=1,Ati n=21,Austronesian n=10,Austronesian n=15,Burmese n=2,CDX n=20,Cambodian n=2,Dai n=5,Dusun n=2,FloresBena n=11,FloresCibal n=12,FloresRampasasa n=20,Igorot n=2,KHV n=28,Kensiu n=9,Kintak n=19,Lahu n=2,Malaysian n=100,Miao n=2,SenoiCheWong n=1,SenoiSemai n=10,SenoiSmakBeri n=2,Temuan n=15,Thai n=2,Yi n=2

a. Southeast Asia

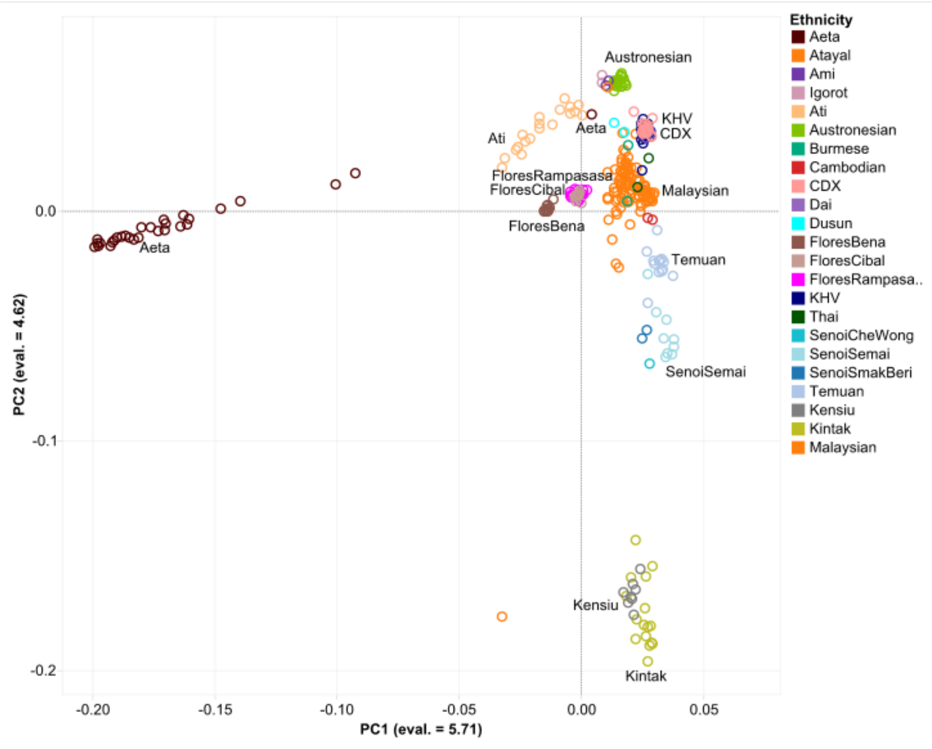

Philippine (Aeta) and Malaysian (Kintak and Kensiu) Negrito populations are the most distinctive among the major Southeast Asian populations. Most populations except for the Negrito groups are closely related or admixed with Austronesians. The Ati are a Philippine Negrito group, however, genetically they are closer to the Austronesian populations than Aeta.

b. South Asia

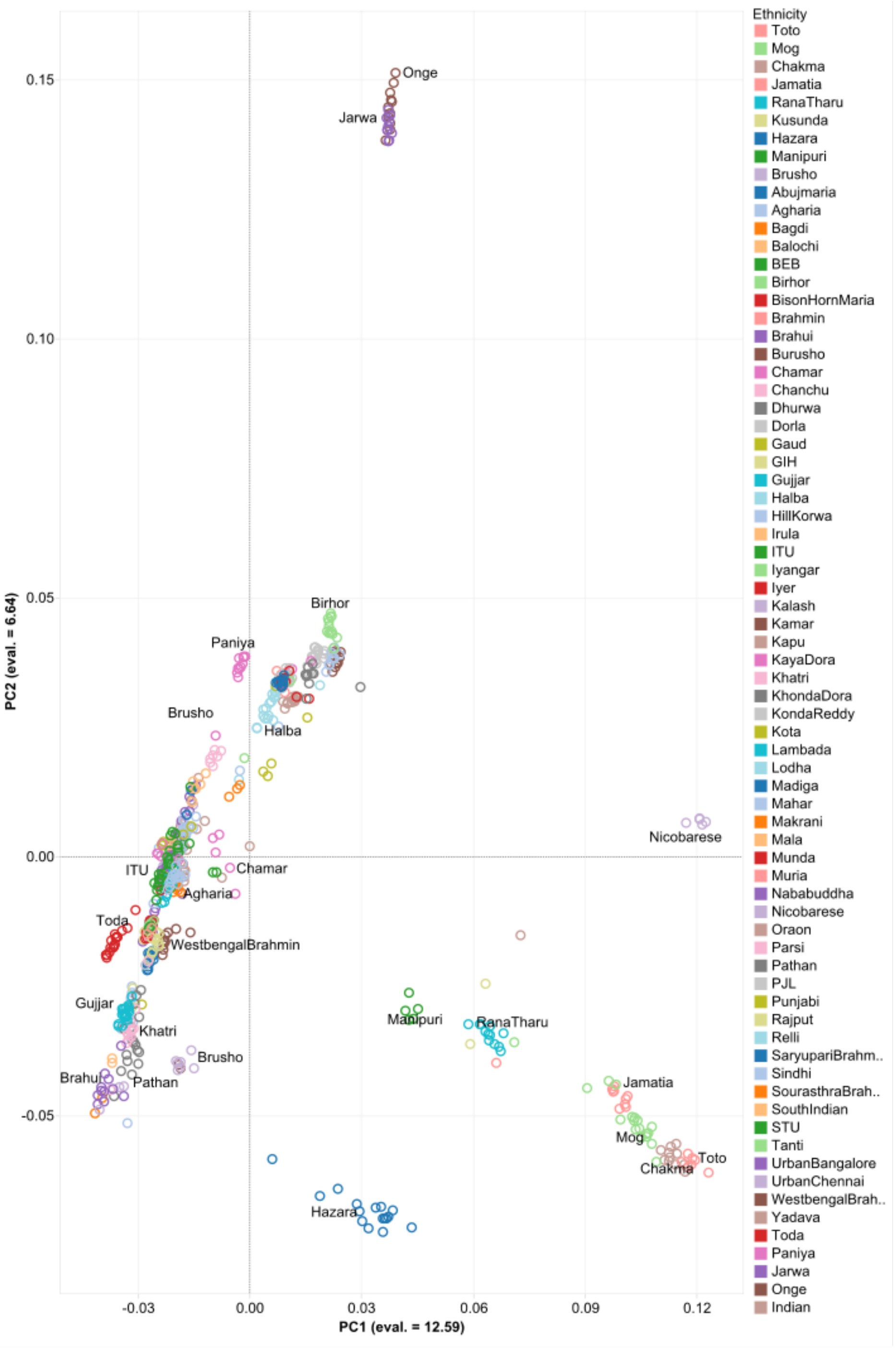

As shown in the Admixture plot in Figure S5.1, The Andaman populations (JAR and ONG) are genetically distinct compared with other South Asian groups. The Nicobarese people (NIC) are not closely related with Andamanese groups even though they live nearby each other. Most of the other populations are closely clustered except for the populations containing East Asian ancestry, such as the Toto (TTO) and Chakma (CHK).

c. Oceania

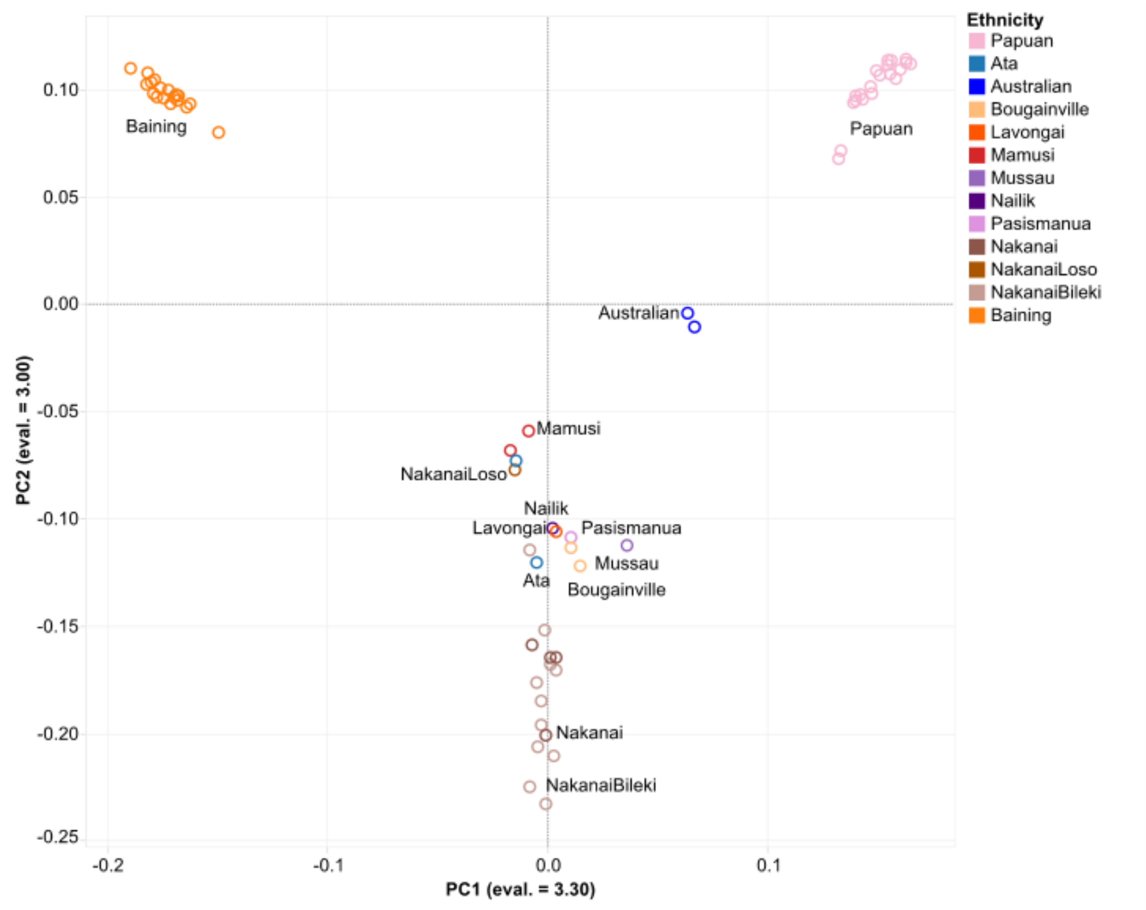

The Baining and Papuan groups are distinct from other Oceanian individuals, who appear to be admixed with Austronesians according to the Admixture analysis.

d. Northeast Asia

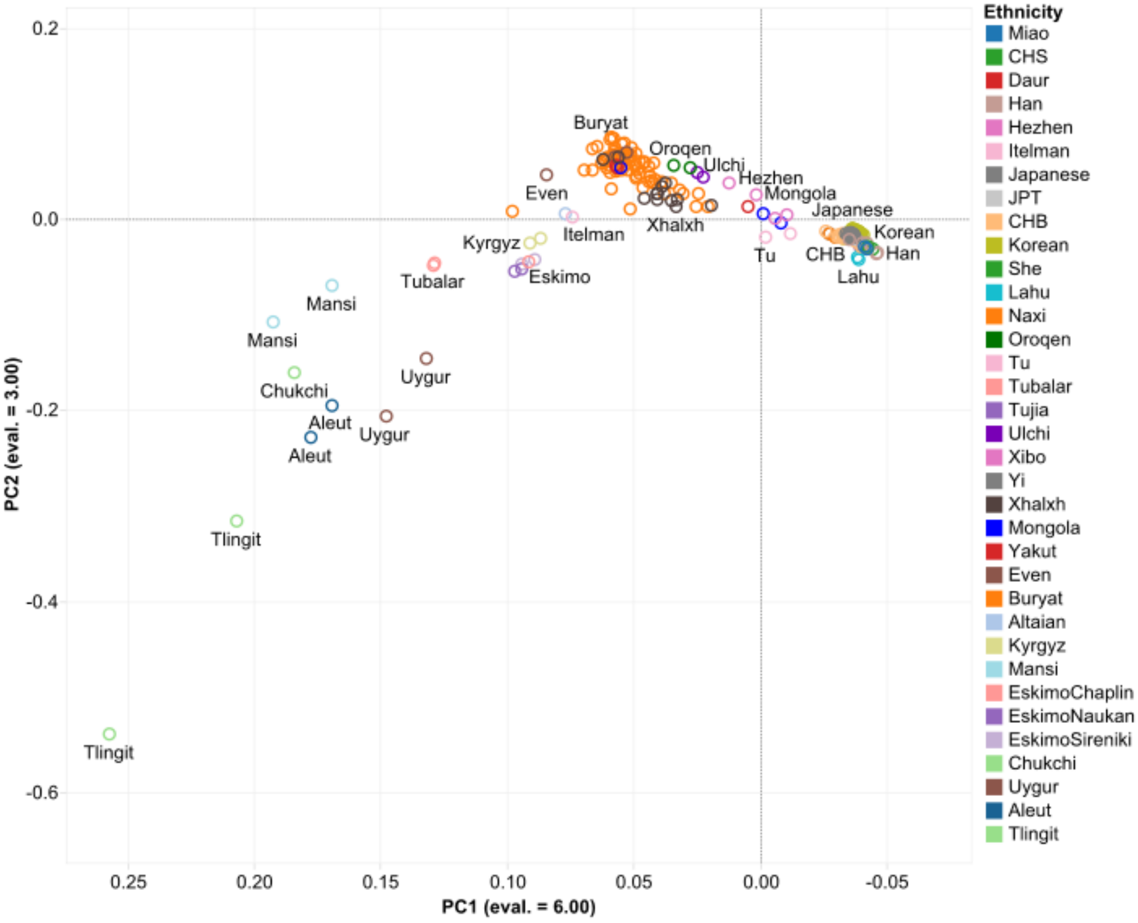

The current major populations in Northeast Asia, Japanese, Korean, and Han Chinese, are closely related each other, and Mongolian groups show larger diversity than these major populations. Many of the other North Asian individuals, such as Uygur and Tlingit people also have European ancestry components and are scattered in the PCA plot.

References

Alexander DH, Novembre J, and Lange K, Fast model-based estimation of ancestry in unrelated individuals, Genome Res. 19:1655-1664, 2009.

Price AL, Patterson NJ, Plenge RM, Weinblatt ME, Shadick NA, and Reich D, Principal components analysis corrects for stratification in genome-wide association studies, Nature Genetics, 38:904-909, 2006.

Supplementary note 5 – F_{ST} analyses

Jiani Li[1], Somasekar Seshagiri[1], Steffen Durinck[1,2]

[1]Dept. of Molecular Biology, Genentech Inc., 1 DNA Way, South San Francisco, CA 94080; [2]Dept. of Bioinformatics and Computational Biology, Genentech Inc., 1 DNA Way, South San Francisco, CA 94080.

author responsible for this section: Steffen Durinck - durinck.steffen@gene.com

Methods

FST Estimation

We calculated F_{ST} (Weir and Cockerham 1984) between pairs of regional groups (**Figure S5.1**). Consistent with the PCA results and those of previous studies, F_{ST} statistics place the sub-Saharan African group (AFR) as an outgroup to all other regional population groups, as expected under the Out of Africa model.

To examine fine-scale patterns of genetic differentiation, we also calculated pairwise F_{ST} values between populations (in the same regional group) with at least 10 individuals (**Figure S5.2**). The ethnic groups within NEA have low F_{ST} values, suggesting recent shared history. For South Asian populations, groups within Pakistan share close affinity. Consistent with MSMC and other analyses, the Jarawa (JAR) and Onge (ONG) are similar to each other, but very different from all other SAS ethnic groups. This reflects both a long period of isolation in the Andaman Islands, as well as increased genetic drift due to small population sizes. In Southeast Asia, we found that the Negrito groups of Malaysia (KIN) and the Philippines (AET and ATI) are highly differentiated from other SEA groups. Finally, our analyses also found substantial allele frequency differences between the Baining and other groups from Papua New Guinea.

Figure S5.1. F_{ST} (Weir and Cockerham 1984) between population pairs. F_{ST} was estimated for each chromosome separately and then averaged across chromosomes.

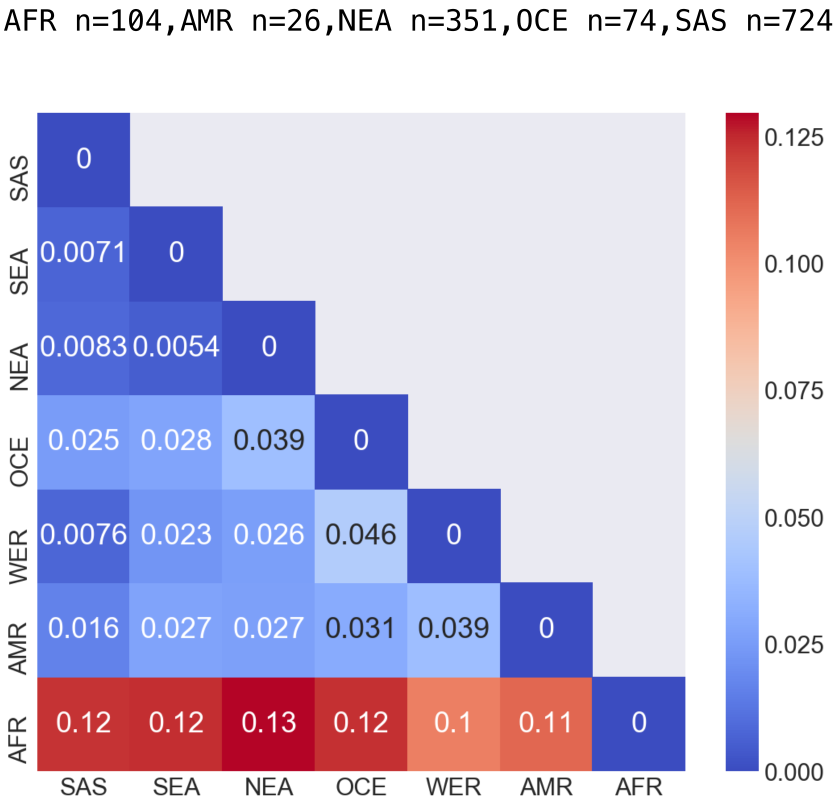

Figure S5.2. F_{ST} between populations within regional groups. F_{ST} was estimated for each chromosome separately and then averaged across chromosomes. Ethnic groups are organized based on the countries. a. Singapore, b. Vietnam. c. Philippines. d. Korea

NEA
BUR n=87,KOR n=152,KHL n=12
OCE
BAI n=19,NKB n=11,PAP n=20
SAS
ABM n=11,AGH n=17,BIR n=20,BRA n=11,CHK n=11,DHR n=10,DOR n=12,GUJ n=20,HAZ n=17,HKR n=10,ITU n=26,SSI n=38,IYE n=13,JAR n=12,KAM n=11,KYD n=11,KHA n=11,KNR n=17,LAM n=17,LOD n=11,MHR n=19,MOG n=20,MUR n=10,ONG n=15,ORN n=15,PNY n=11,PAT n=17,RAJ n=14,RTH n=12,SRB n=14,SND n=12,TOD n=20,TTO n=11,BLR n=34,MAA n=34, WBB=10
SEA

AET n=29,ATI n=21,MEN n=10,NIA n=15,BEN n=11,CIB n=12,KHV n=28,KIN n=19,MLY n=100,SNS n=10,RAM=20, TEM n=15

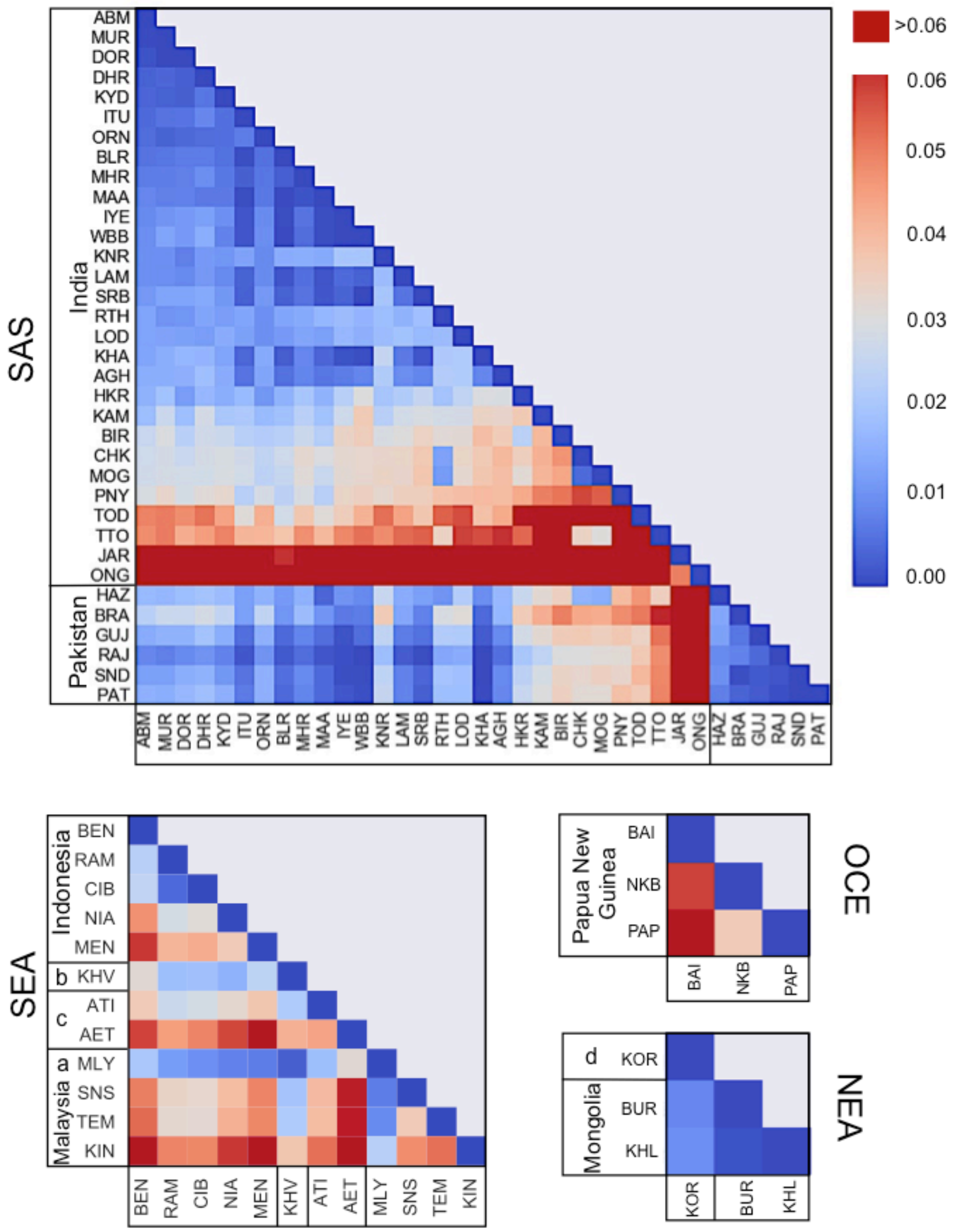

Reference

Weir BS, Cockerham CC (1984) Estimating F-Statistics for the analysis of population structure. Evolution 38: 1358-1370.

Supplementary note S6 – Using patterns of allele sharing to construct population trees

Jeffrey D. Wall[1]
[1]Institute for Human Genetics, University of California, San Francisco, California 94143, USA.

author responsible for this section: Jeff Wall - Jeff.Wall@ucsf.edu

We use a parsimony-based analysis of allele sharing, described in Wall (2017), to elucidate the historical branching orders for non-African groups. We focused on variants not present in sub-Saharan Africans or in archaic humans, and thus likely to have arisen after the dispersal of modern humans out of Africa. We also assumed that West and Central Africans are an outgroup to all non-Africans (though this assumption can be relaxed). Qualitatively, shared derived alleles within a pair of non-African genomes (roughly) correspond to mutations that occurred after the time when modern humans left Africa but before the time where the two genomes share a common ancestor (at the particular genomic location where the mutation occurred). The expected number of shared derived alleles is proportional to the average internal branch length in the unobserved genealogies of sampled individuals, averaged across the whole genome. In this context, a greater number of shared derived alleles indicates a shorter average time to the most recent common ancestor between two individuals, and thus greater genetic relatedness. While our approach is quite similar to previously proposed methods for quantifying ancestry such as D-statistics (Green et al. 2010), enhanced D-statistics (Meyer et al. 2012) or the D_{4P} statistic (Rasmussen et al. 2011), we tailored our approach to minimize the confounding effects of segregating ancestral polymorphism and admixture with archaic human groups such as Neanderthals and Denisovans. See Wall (2017) for further details.

Comparing continental populations

We considered representative populations from three main non-African groups: Europeans, East Asians and Melanesians. Given a single sequence representative from each group, there are three possible tree topologies, each containing a single internal branch and a unique phylogenetically informative variant (PIV) pattern (**Figure S8.1**). Specifically, if exactly 2 out of 3 of the sequences share a derived allele, then the genealogy at this site has (under the parsimony assumption) the two populations with a derived allele as sister groups. While the true topologies are expected to vary across the genome due to incomplete lineage sorting and additional demographic factors (e.g., migration), the relative number of PIV's supporting each topology is informative about

the average genealogical history of the samples and thus the true branching order of the populations.

We started by considering only biallelic SNPs that are homozygous ancestral in 49 West and Central Africans, Neanderthal and Denisovan. We also filtered out repetitive regions using the same filters as for the estimation of variant discordance rate with the 1000 Genomes Project data. Then, for 3 non-African (haploid) test sequences, we tabulated the fraction of PIVs supporting each topology. We used two different European populations (GBR and CEU), two different East Asian populations (Japanese and Han) and two different Melanesian populations (Papuan and Baining), and averaged across all possible combinations of haploid samples within each test population. The results were remarkably consistent across populations, with ~44% of the PIVs shared between the East Asian and Melanesian sequences (P_{AM}, **Table S6.1**).

Figure S6.1 Possible genealogies for non-African populations. We assume a single (haploid) sampled sequence from each of 4 populations, and that the African sequence is the outgroup. Each genealogy has a single, phylogenetically informative, internal branch (shown in red), leading to a unique pattern of ancestral (A) and derived (D) alleles. The proportion of phylogenetically informative sites supporting each topology are denoted by P_{EA}, P_{EM} and P_{AM} for Figures **S6.1a**, **S6.1b** and **S6.1c** respectively.

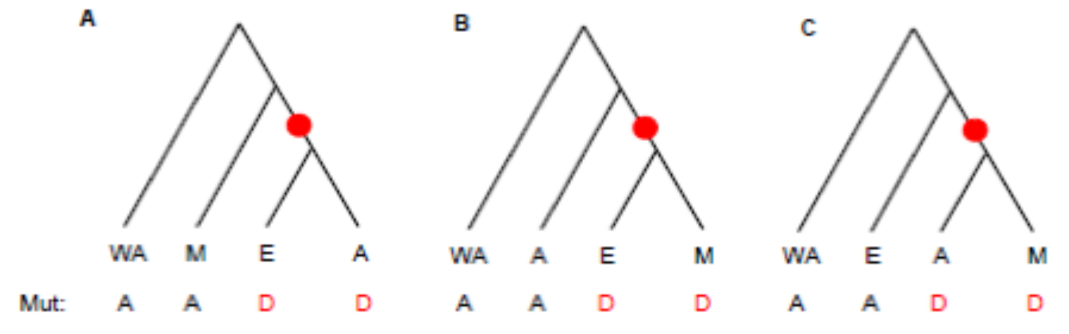

Table S6.1 Proportion of phylogenetically informative sites supporting each topology. See also **Figure S6.2.**

Populations	P_{EA} (%)	P_{EM} (%)	P_{AM} (%)
GBR, Japanese, Papuan	29.1	26.8	44.1
GBR, Japanese, Baining	28.7	26.9	44.4
GBR, Han, Papuan	29.2	26.8	44.1
GBR, Han, Baining	28.8	26.8	44.4

CEU, Japanese, Papuan	28.9	26.8	44.2
CEU, Japanese, Baining	28.5	27.0	44.5
CEU, Han, Papuan	29.0	26.7	44.2
CEU, Han, Baining	28.6	26.9	44.5

Figure S6.2 Schematic showing the proportion of informative sites supporting each possible topology of Melanesians (Me), Europeans (Eu) and East Asians (As) as well as potential migration routes consistent with these topologies.

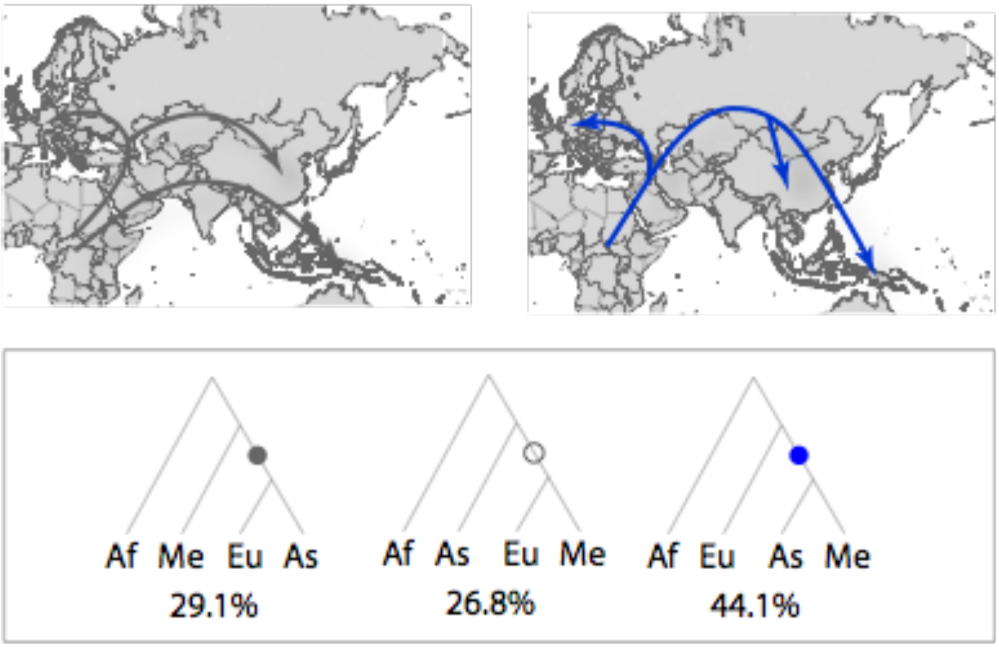

So, while the 'true' demographic history of non-African populations was certainly very complex, on average Melanesian and East Asian sequences are more genetically similar (and share a more recent common ancestor) than do European and East Asian or European and Melanesian sequences (**Figure S6.2**).

Within-continent comparisons

We used a similar approach to what is described above to generate population trees for more than 3 non-African populations at a time. Specifically, given n different test populations, we considered every possible pair of populations in turn and tabulate the

average number of shared derived alleles for single haploid representatives (of each of the two populations being considered). Then, we identified the pair of populations with the highest average number of shared derived alleles, merged them into a single group, and repeated the process with the remaining (n-1) populations. This is functionally equivalent to the neighbor-joining algorithm of Saitou and Nei (1987).

We used a bootstrap resampling approach to assess the confidence we had in individual nodes in the tree (i.e., pairs of populations that were merged). Given populations A, B and C, we quantified the support for each of the three possible branching orders by resampling the number of PIVs supporting each topology. For computational convenience, we approximated the distribution of the number of resampled PIVs with a normal distribution with variance equal to the mean (i.e., we used a normal approximation for Poisson distributed random variables). We ran 10^4 bootstrap replicates for each node, and only displayed those nodes with support value > 0.5. Results are shown in **Figure S6.3**. Within South Asian populations, we do not find a strong association between language and genetic similarity – both the Rana Tharu and Mahar, Indo-European language speaking groups, are more closely related to non-Indo-European language speaking groups than to each other. Similarly, the Brahui, a Dravidian language speaking group from Pakistan, is not closely related to any other Dravidian language speaking group.

We note in passing that the specific populations included in the analyses can have small (but noticeable) effects on the branching orders of specific populations. This explains the slightly different location of the Ati and Aeta in the figures. Also, since the method effectively averages genealogies across the genome, it has some difficulties in the placement of recently admixed populations such as the Ati.

Figure S6.3. Allele sharing trees for select (a) Southeast Asian, (b) South Asian and (c) non-African populations. Panel c also includes information on Negrito populations and Denisovan admixture estimates.

a **b**

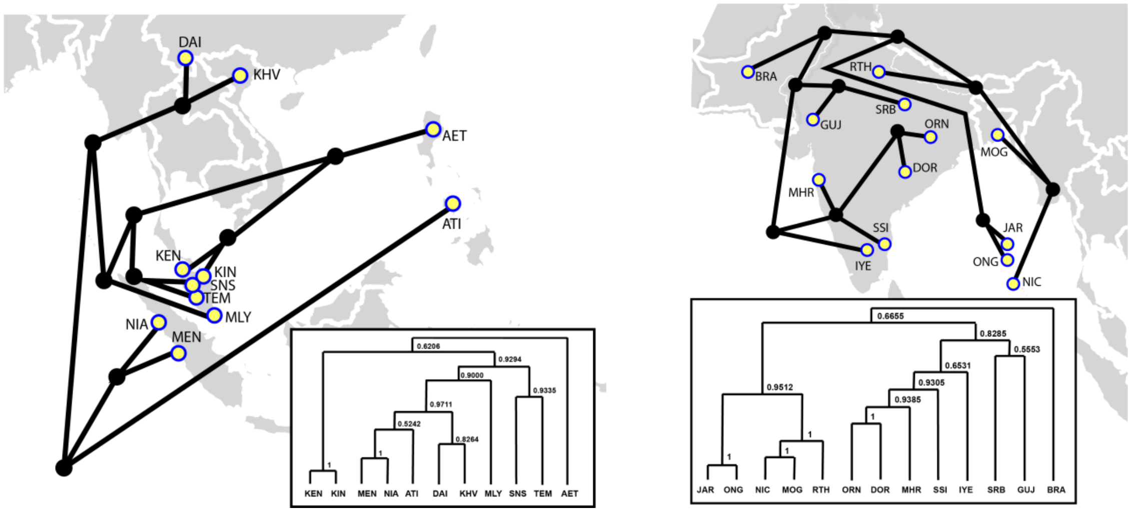

c

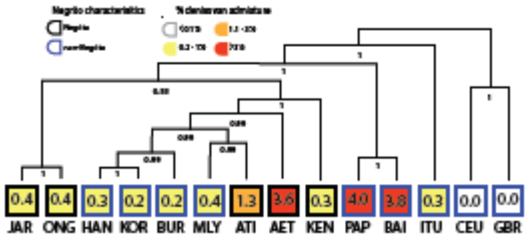

References

Green RE, Krause J, Briggs AW, Maricic T, Stenzel U, et al. (2010) A draft sequence of the Neandertal genome. Science 328: 710-722.

Meyer M, Kircher M, Gansauge MT, Li H, Racimo F, et al. (2012) A high-coverage genome sequence from an archaic Denisovan individual. Science 338: 222-226.

Rasmussen M, Guo X, Wang Y, Lohmueller KE, Rasmussen S, et al. (2011) An Aboriginal Australian genome reveals separate human dispersals into Asia. Science 334: 94-98.

Saitou N, Nei M (1987) The neighbor-joining method: a new method for reconstructing phylogenetic trees. Mol. Biol. Evol. 4: 406-425.

Wall JD (2017) Inferring human demographic histories of non-African populations from patterns of allele sharing. Am. J. Hum. Genet. 100: 766-772.

Supplemental note S7 - Footprints of positive selection

Elena S. Gusareva[1] and Hie Lim Kim[1,2]

[1]Singapore Centre for Environmental Life Sciences Engineering, Nanyang Technological University, Singapore 637551. [2]The Asian School of the Environment, Nanyang Technological University, Singapore 637459.

author responsible for this section:
Elena S. Gusareva - egusareva@ntu.edu.sg

Methods

We defined 12 reference groups based on the hierarchical clustering of individuals using Admixture (see details in Supplementary section S4), and used these to search for footprints of recent positive selection. We adopted the Integrated Haplotype Score (*iHS*) test for capturing haplotype homozygosity based signals of positive selection (Voight et al. 2006). For this test, we assumed that the ancestral allele matched the orthologous chimpanzee nucleotide base. If a SNP's ancestral state could not be determined, it was discarded from the analysis. We confined our analyses to populations with at least 20 individuals, in order to reduce small-sample artefacts with the *iHS*-score (Pickrell et al. 2009). First, the unstandardized *iHS* was calculated using Selscan v. 1.1.0 (Szpiech and Hernandez 2014). We used the maximum allowed gap between loci when assembling haplotypes of 500 Kb and "--skip-low-freq" flag to pre-filter all sites with MAF<0.05. We then standardized the *iHS* values in R v3.1.3, using the *rehh* package (Gautier and Naves 2011). In this package, *iHS* scores were further transformed into $p_{iHS} = -\log[1-2|\Phi(iHS) - 0.5|]$, where $\Phi(x)$ represent the Gaussian cumulative distribution function. Assuming *iHS* values are normally distributed (under neutrality), p_{iHS} can be interpreted as $\log_{10}(1/P)$ where P is the two-sided P-value associated to the neutral hypothesis (no selection). The *iHS*-scores were considered as outliers (and potentially indicative of positive selection) when $p_{iHS} \geq 7$.

Results

Many of the positively selected regions detected in this analysis spread over all autosomes except for chromosome 21 displayed prominent signals of positive selection in at least one of the tested groups (Figure S7.1). The number and position of the loci under selection varied across the groups. Most of the identified selection regions were either population-specific (occur exclusively in one group) or shared between only two or three groups. A strong selection signal in the HLA locus on chromosome 6 was characteristic for all the groups but Philippine Negrito (Figure S13.2).

Figure S7.1. Potential positive selection signals plotted over the autosomes by 12 reference groups. The strongest iHS scores (p_{iHS}>7) are shown.

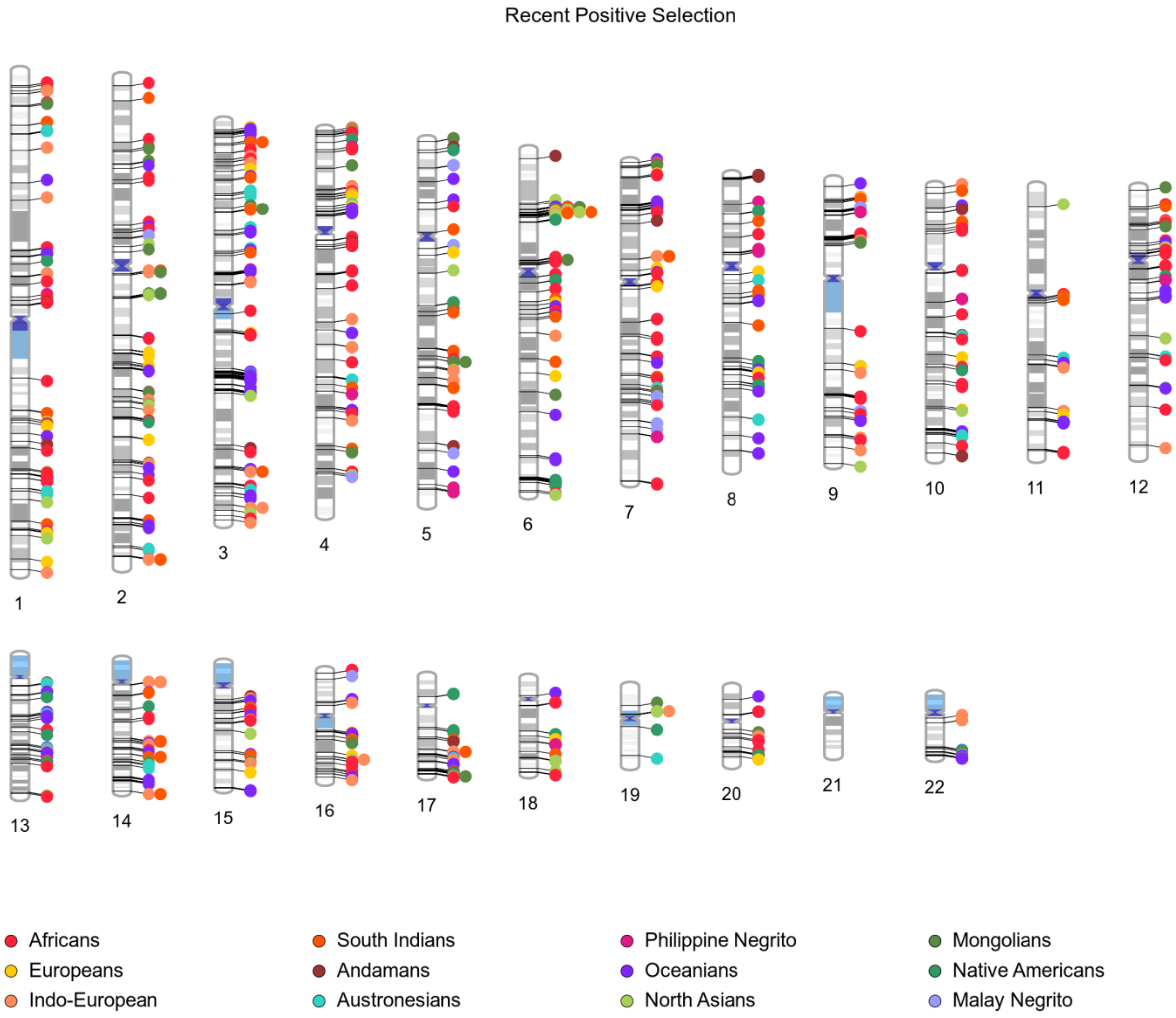

Figure S7.2. Loci of prominent positive selection on chromosome 6 plotted for 12 ancestral groups and pooled data of 1178 individuals.

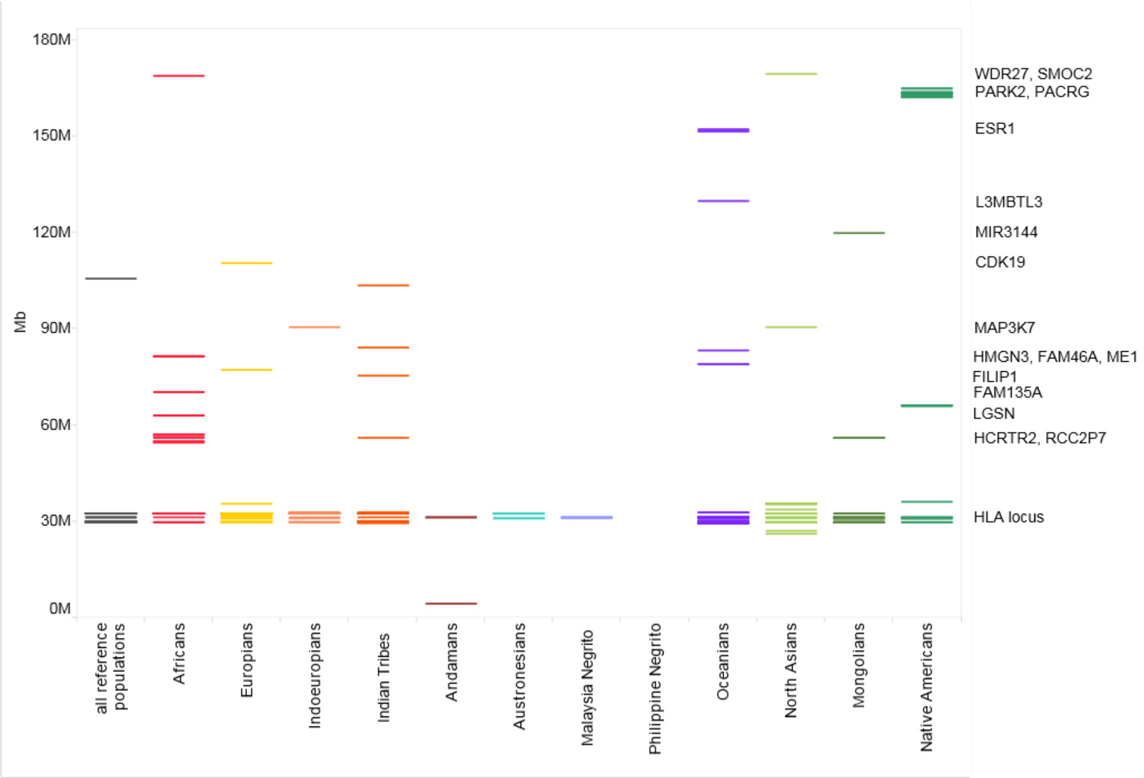

Reference

Gautier M, Naves M (2011) Footprints of selection in the ancestral admixture of a New World Creole cattle breed. Mol Ecol 20: 3128-43. doi: 10.1111/j.1365-294X.2011.05163.x

Pickrell JK, Coop G, Novembre J, Kudaravalli S, Li JZ, Absher D, Srinivasan BS, Barsh GS, Myers RM, Feldman MW, Pritchard JK (2009) Signals of recent positive selection in a worldwide sample of human populations. Genome Res 19: 826-37. doi: 10.1101/gr.087577.108

Szpiech ZA, Hernandez RD (2014) selscan: an efficient multithreaded program to perform EHH-based scans for positive selection. Mol Biol Evol 31: 2824-7. doi: 10.1093/molbev/msu211

Voight BF, Kudaravalli S, Wen X, Pritchard JK (2006) A map of recent positive selection in the human genome. PLoS Biol 4: e72. doi: 10.1371/journal.pbio.0040072

Supplementary note S8 – Analyses of the non-recombining portion of the Y chromosome

Vadim Stepanov[1]
[1]Institute of Medical Genetics, Tomsk National Medical Research Center, Russian Academy of Sciences, Tomsk 634050, Russian Federation, and Tomsk State University, Tomsk 634050, Russian Federation.

author responsible for this section: Vadim Stepanov - vadim.stepanov@medgenetics.ru

Methods

High-resolution assessment of Y-chromosome binary haplogroups for 956 male samples in the final GAsP dataset was conducted from the vcf file using an in-house Python algorithm. Composition of polymorphic Y-chromosomal sites in each sample was compared to an in-house database containing information of confirmed Y-chromosomal SNPs, their genomic position, ancestral and derived alleles, and branching on the human Y-chromosomal phylogenetic tree according to the nomenclature for the tree of human Y-chromosomal binary haplogroups developed by the human Y-Chromosome Consortium (YCC 2002) with subsequent modifications (Karafet et al. 2008; ISOGG 2017). Variants in genomic positions not present in the current ISOGG database were not used for Y haplogroup classification. Results of automatic assessment of Y haplogroup in each sample were verified manually by checking terminal and major branching SNPs.

Phylogenetic trees were constructed using software package BEAST v.1.8.4 (Drummond et al. 2012) as described in (Karmin et al., 2015). GTR substitution model was selected for the Y data. For phylogenetic trees of haplogroups and individuals BEAST analyses were run for 10 million iterations, sampling every 5,000 steps, with strict clock and 10% burn-in.

Results

One hundred and nineteen Y-chromosomal haplogroups were identified (**Supplementary Table S3d**) across the 956 male samples (representing 54 populations; includes 142 individuals for whom the gender was assigned based on Y chr data) that were analyzed. In general Y-chromosomal variability in Asian GAsP samples follows the same geographic patterns that were found in previous studies (Qamar et al. 2002; Sengupta et al., 2006; Xue et al. 2006). Haplogroups of Western Eurasian origin (R1a1, R1b, J) predominate in Pakistan and North-Western India, while in Southern and Eastern India the majority of male lineages belong to sub-clades of haplogroup H (15

sub-clades) and L1a. Eastern Eurasian haplogroups O1b and O2 (32 sub-clades in total) are the major Y chromosomal variants in East and South-East Asia. In New Guinea M and K2b1 are two predominant haplogroups.

Very specific compositions of haplogroups and low Y-chromosomal within-population diversity was found in isolated island populations – Philippine Negritos, Flores islanders and Andamanese. The Aeta and Ati, two Philippine Negrito populations, are characterized by very different male lineages. The major haplogroup in the Aeta (K2b1c) is closely related to K2b1, found in some Papuan populations, while the Ati share the majority of their Y-chromosomal variants with Austronesians. All male Flores islanders have Y haplogroups that belong to the very rare C1b1a2 sub-clade, while the Y chromosomes of all Andamanese males belong to the D1 haplogroup. The Y-chromosomal population structure corresponds well to population relationships inferred from autosomal genome variants by PCA, MSMC and Admixture analysis (supplementary section S3 and S4).

References

ISOGG. Available at http://www.isogg.org/. Date of access: March 3, 2017.

Karafet T.M., Mendez F.L., Meilerman M.B., et al. New binary polymorphisms reshape and increase resolution of the human Y chromosomal haplogroup tree. Genome Res. 2008. V.18. P.830-838.

Qamar R., Ayub Q., Mohyuddin A. et al. Y-Chromosomal DNA Variation in Pakistan. Am. J. Hum. Genet. 2002. V. 70. P. 1107-1124.

Sengupta S., Zhivotovsky L.A., King R. et al. Polarity and temporality of high resolution Y-chromosome distributions in India identify both indigenous and exogenous expansions and reveal minor genetic influence of central Asian pastoralists. Am. J. Hum. Genet. 2006. V.78. P. 202-221.

Xue Y., Zerjal T., Bao W. et al. Male Demography in East Asia: A North-South Contrast in Human Population Expansion Times. Genetics. 2006. V. 172. P. 2431-2439.

The Y-Chromosome Consortium. A nomenclature system for the tree of human Y-chromosmal binary haplogroups. Genome Research. 2002. V. 12. P. 339-348.

Drummond AJ, Suchard MA, Xie D & Rambaut A. Bayesian phylogenetics with BEAUti and the BEAST 1.7 Molecular Biology And Evolution. 2012. 29: 1969-1973.

Karmin M, Saag L, Vicente M3 et al. A recent bottleneck of Y chromosome diversity coincides with a global change in culture. Genome Res. 2015 Apr;25(4):459-66. doi: 10.1101/gr.186684.114.

Figure S8.1. Y-haplogroup distribution in GAsP dataset. a-b. across all samples (a). and samples from Indian subcontient (b).

a

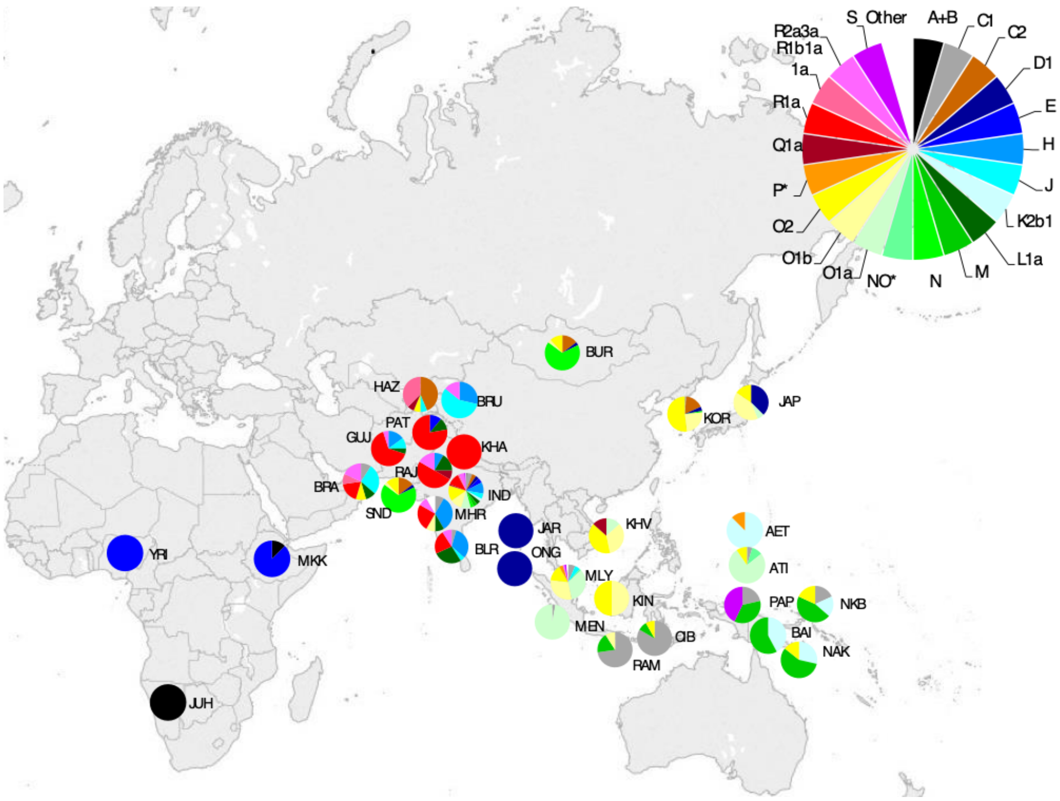

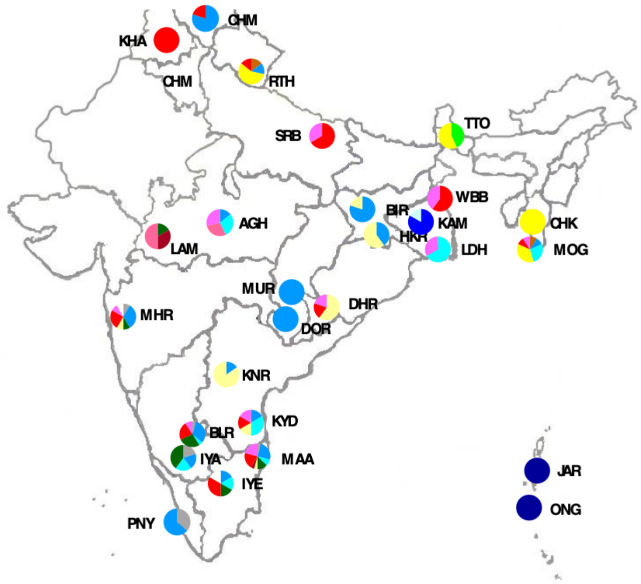

Figure S8.2. Y chromosome haplogroup phylogenetic tree based on BEAST analyses. The phylogenetic tree of 956 whole Y chromosome sequences reconstructed using BEAST. Samples have been collapsed according to haplogroups and subclades. Only main haplogroup and branches labels are shown. Colors indicate phylogenetic belonging of samples

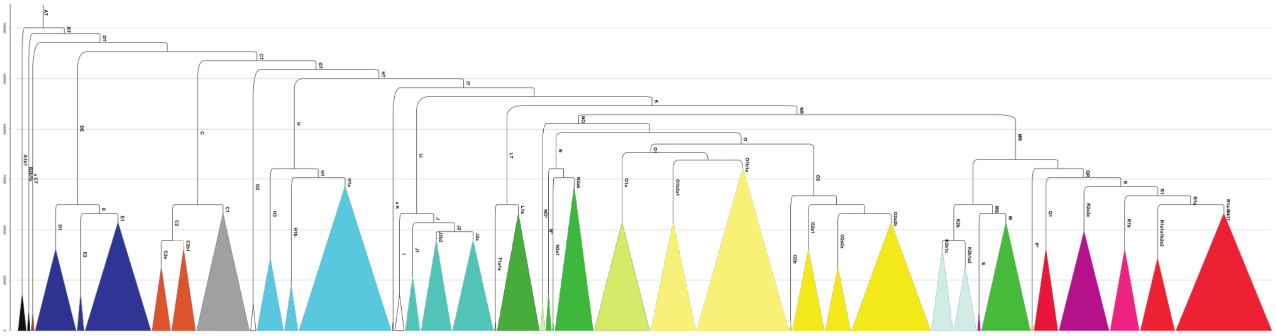

Supplementary note S9 – Mitochondrial and Y-chr distribution in population groups.

Kushal Suryamohan[1], Eric Stawiski[1,2], Somasekar Seshagiri[1]

[1]Molecular Biology Department, Genentech, Inc., South San Francisco, California 94080, USA. [2]Department of Bioinformatics and Computational Biology, Genentech, Inc., South San Francisco, California 94080, USA.

Results

Circos plots (Gu, Z. 2014) were generated for each language group or (**Figure S9.1**) orthe 7 regions based on mtDNA and Y-chr haplogroup calls for each sample in our GAsP data set (**Figure S9.2**) to reveal unique and common region-specific mtDNA-Y-chromosome haplogroup combinations. Custom-made quilt plots (**Figure S9.3 and S9.4**) were generated to understand patterns of haplogroup sharing between tribes, caste or ethnic groups in South Asian samples. These plots revealed haplogroup differences among social classes and language groups within the Indian subcontinent.

Interestingly within India, the Y haplogroup R1a was predominantly observed in upper caste groups (upper, middle or lower groups sum to ~28%) compared to Adivasi (~4.7%; p = 1.532e-08). Further, Indo-European language groups had a higher proportion of Y-chromosomal R1a haplogroup (~35%) compared to Dravidian (12% R1a; 28% H), Sino-Tibetan (14% R1a; 55% O2), or Austroasiatic (59% H) language groups ($p<0.05$) (**Figure S9.1**).

Figure S9.1. Maternal and paternal haplogroup quilt plots for South Asian samples stratified by language groups (IE – Indo-European; DR – Dravidian) and social hierarchy (Tribal, lower/middle/upper caste). Each column in the quilt plot represents a sample.

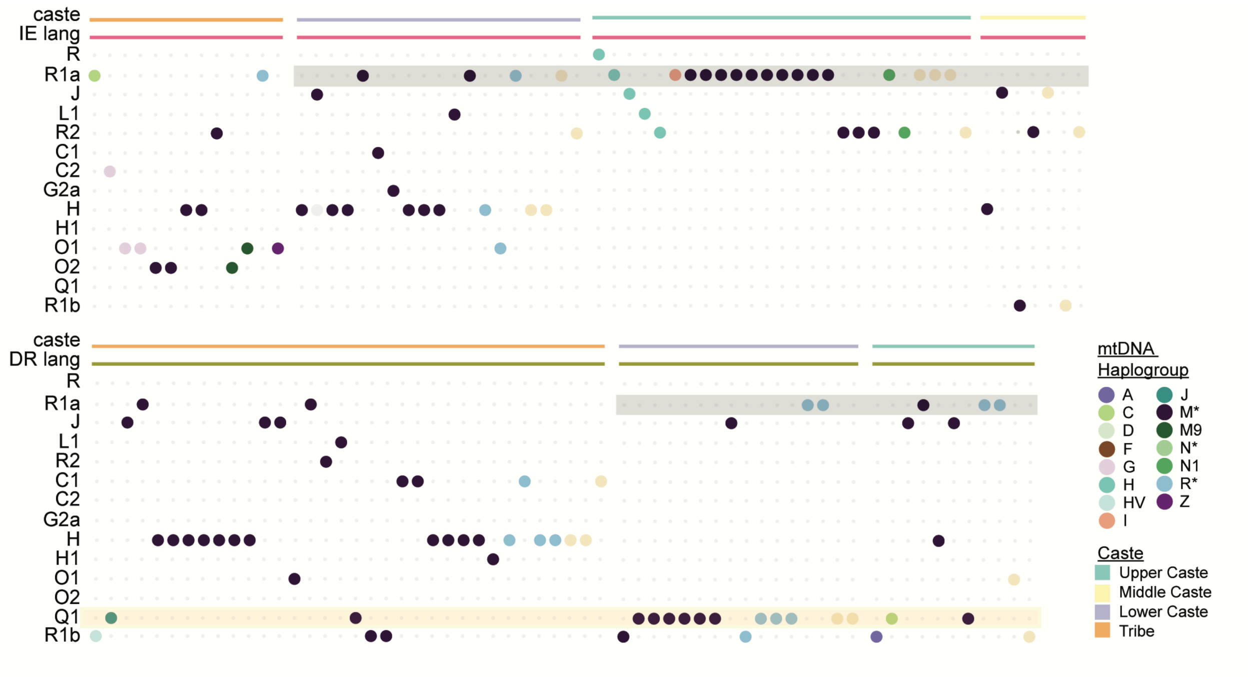

Figure S9.2 Quilt Plot Samples from two different language groups (Tibeto Burman and Austro-Asiatic) differ in Y haplogroup sharing patterns. Tibeto Burman samples predominantly carry the O2 Y-chr haplogroup while Austro-Asiatic samples carry haplogroup H. However, both sets of samples predominantly share mitochondrial haplogroups M* and R.

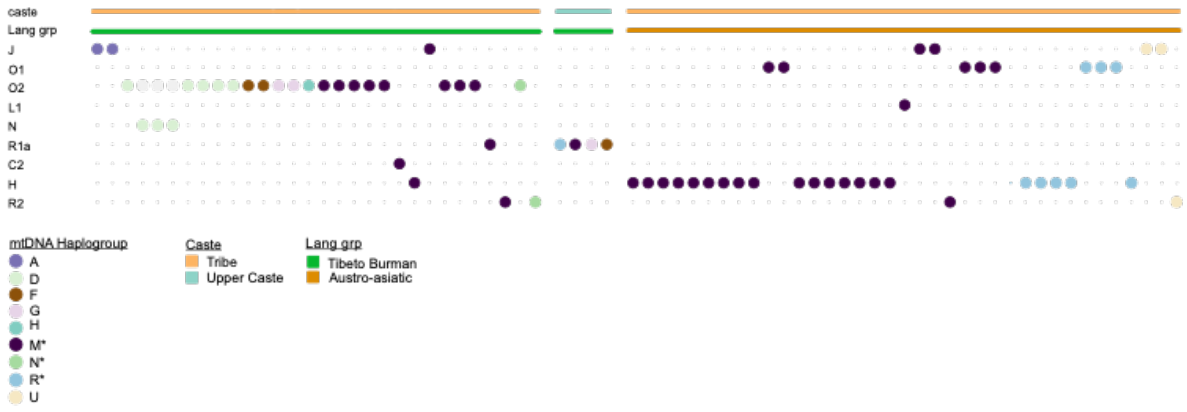

Figure S9.3. Circos plots that indicate maternal and paternal haplogroup sharing in caste groups and tribal groups within South Asia (SAS).

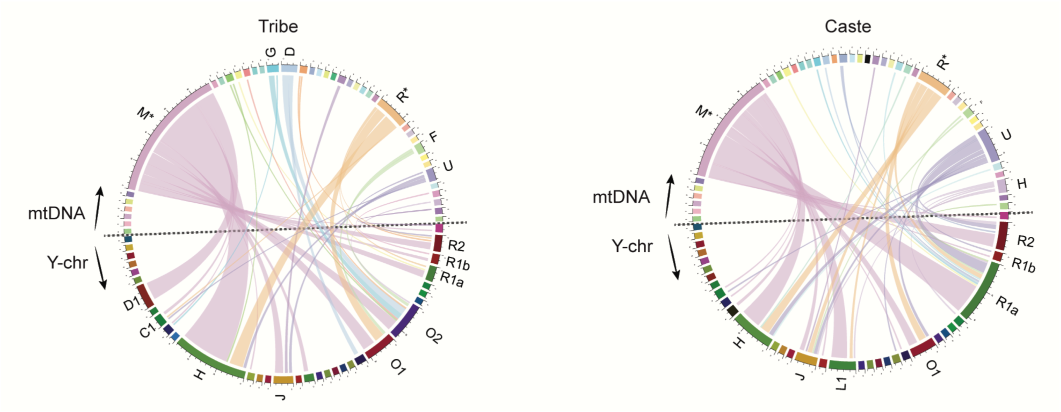

Figure S9.4 Circos Plot representation of mtDNA and Y Haplogroups for samples within a given region. a Africa (AFR); **b**, North America (AMR); **c**, Northeast Asia (NEA); **d**, Oceania (OCE); **e**, South Asia (SAS); **f**, Southeast Asia (SEA); **g**, West Eurasia. mtDNA haplogroups are preceded by ‘m|’ and Y-chr haplogroups by ‘Y|’, with each haplogroup ordered by approximate time of divergence.

a (AFR)

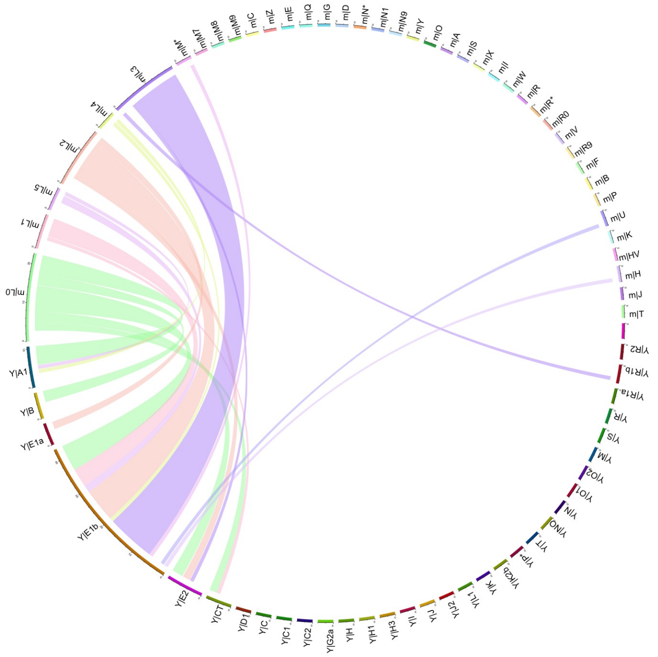

b (AMR)

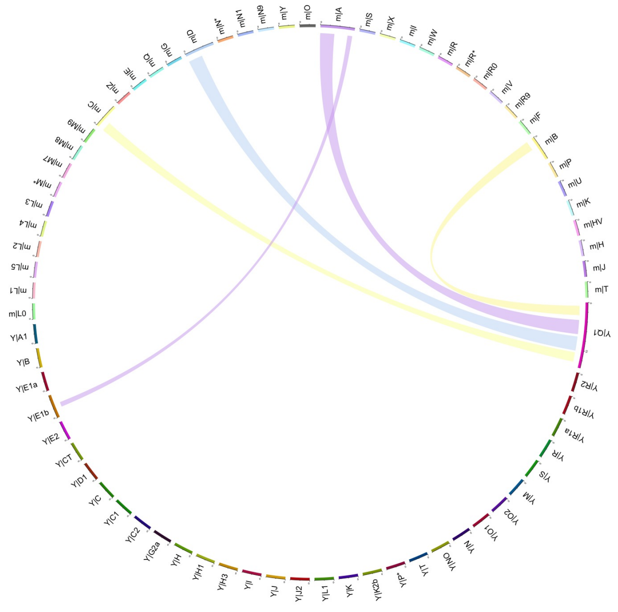

c (NEA)

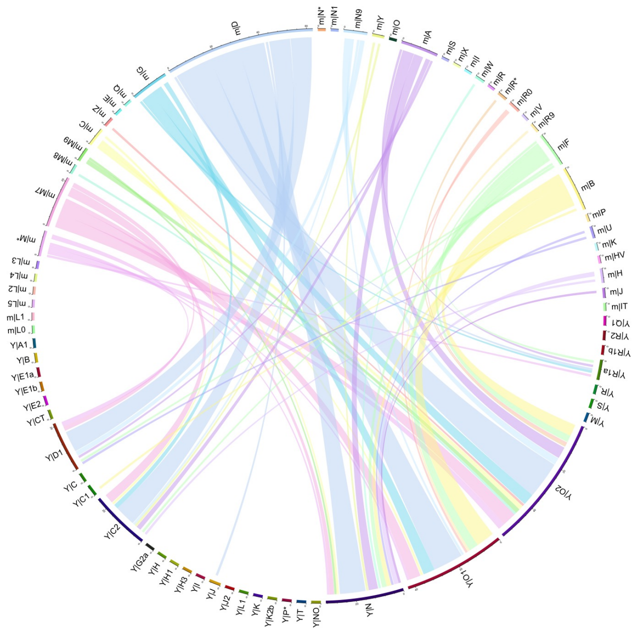

d (OCE)

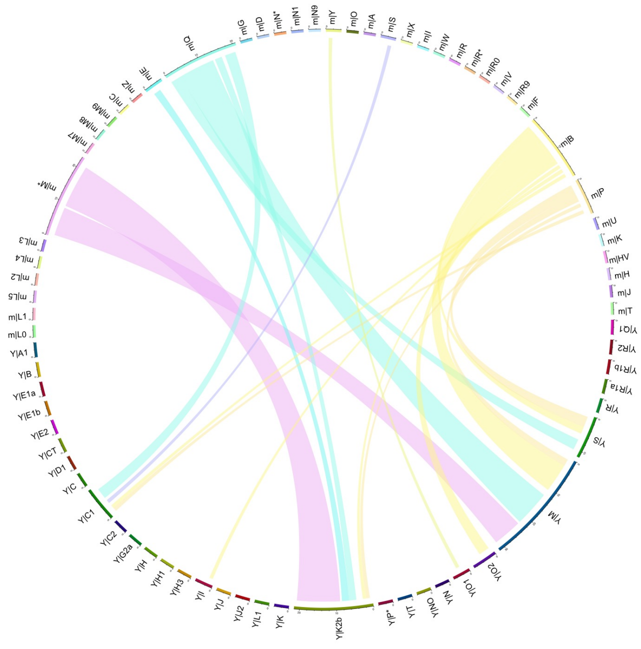

e (SAS)

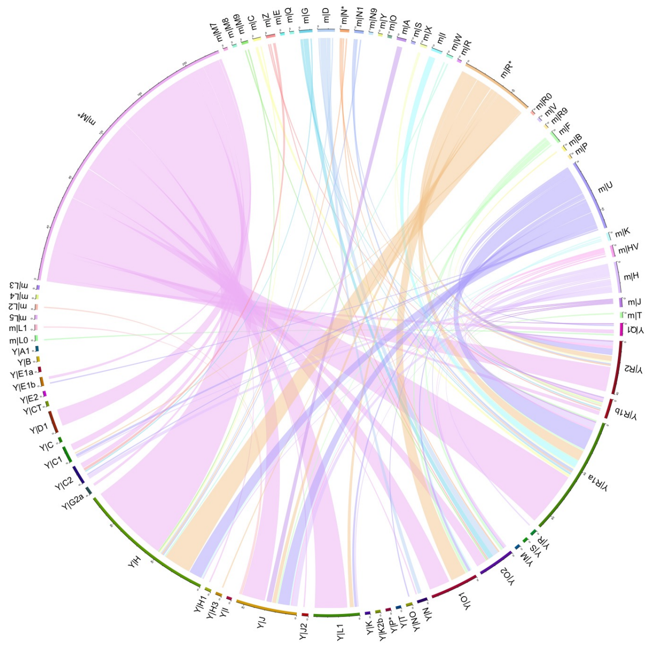
m|M*
m|M7
m|M8
m|M9
m|C
m|Z
m|E
m|Q
m|G
m|D
m|N*
m|N1
m|N9
m|Y
m|O
m|A
m|S
m|X
m|I
m|W
m|R
m|R*
m|R0
m|V
m|R9
m|F
m|B
m|P
m|U
m|K
m|HV
m|H
m|J
m|T
Y|Q1
Y|R2
Y|R1b
Y|R1a
Y|R
Y|S
Y|M
Y|O2
Y|O1
Y|N
Y|NO
Y|T
Y|P
Y|K2b
Y|K
Y|L1
Y|J2
Y|J
Y|I
Y|H3
Y|H1
Y|H
Y|G2a
Y|C2
Y|C1
Y|C
Y|D1
Y|CT
Y|E2
Y|E1b
Y|E1a
Y|B
Y|A1
m|L0
m|L1
m|L5
m|L2
m|L4
m|L3

f (SEA)

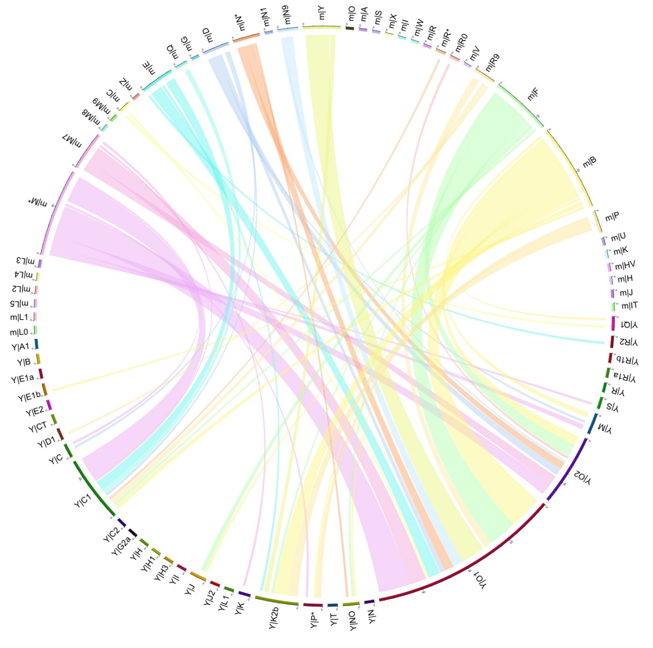
m|M*
m|M7
m|M8
m|M9
m|C
m|Z
m|E
m|Q
m|G
m|D
m|N*
m|N1
m|N9
m|Y
m|O
m|A
m|S
m|X
m|I
m|W
m|R
m|R*
m|R0
m|V
m|R9
m|F
m|B
m|P
m|U
m|K
m|HV
m|H
m|J
m|T
Y|Q1
Y|R2
Y|R1b
Y|R1a
Y|R
Y|S
Y|M
Y|O2
Y|O1
Y|N
Y|NO
Y|T
Y|P*
Y|K2b
Y|K
Y|L1
Y|J2
Y|J
Y|I
Y|H3
Y|H1
Y|H
Y|G2a
Y|C2
Y|C1
Y|C
Y|D1
Y|CT
Y|E2
Y|E1b
Y|E1a
Y|B
Y|A1
m|L0
m|L1
m|L5
m|L2
m|L4
m|L3

g (WER)

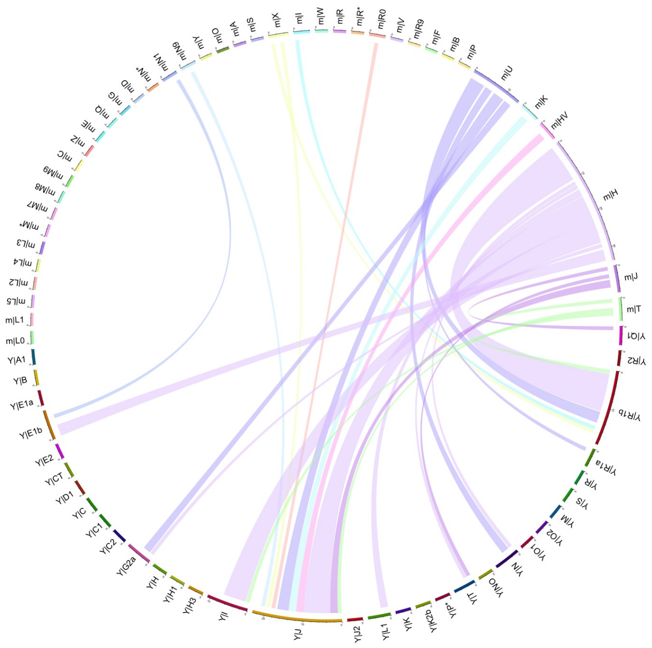

References

Gu Z, Gu L, Eils R, Schlesner M, Brors B. Circlize implements and enhances circular visualization in R. Bioinformatics. 2014 Oct;30(19):2811-2. doi: 10.1093/bioinformatics/btu393. Epub 2014 Jun 14.

Supplementary note S10 – Estimating Neanderthal and Denisovan ancestry

Jeffrey D. Wall[1]
[1]Institute for Human Genetics, University of California, San Francisco, California 94143, USA.

author responsible for this section: Jeff Wall - Jeff.Wall@ucsf.edu

We used two related procedures to identify SNPs and haplotypes likely to be inherited from Neanderthals or Denisovans. For the former, we used a protocol similar to the 'enhanced' D-statistic approach used by Prufer et al. (2014) and Mallick et al. (2016). Specifically, to identify SNPs likely to be inherited from Neanderthals, we first tabulated sites where

1. The reference allele matched the ancestral allele
2. There were no derived alleles in a panel of 49 West and Central African genomes (YRI, GWD, MSL, ESN, MAN, MBU and BIA), and this panel contained < 10% missing genotypes
3. The Neanderthal genotype is homozygous derived
4. The Denisovan genotype is homozygous ancestral

These sites are enriched for mutations inherited from recent admixture with Neanderthals. Note that we implicitly assume there is no Neanderthal admixture into the ancestors of the 49 West and Central Africans. Then, separately for each individual, we tabulated the number of derived alleles (Nalleles) contained at these sites. Since we are counting alleles (and not SNPs), homozygous derived genotypes are counted twice. The number of derived Denisovan alleles (Dalleles) were calculated in an analogous way. For all populations with at least 3 individuals, **Table S10.1** shows the average number of Dalleles and Nalleles.

In a related approach, we also identified putative Neanderthal (and Denisovan) haplotypes by finding groups of correlated SNPs from the Nalleles (and Dalleles) described above (Malaspinas et al. 2016). Specifically, we required

1. 4 or more SNPs with completely correlated genotype calls ($r^2 = 1$)
2. The distance between consecutive SNPs identified in step 1 is ≥ 10 bp and ≤ 10 Kbp
3. The overall frequency of the putative archaic haplotype is > 0.1% and < 50%

While we informally use the word 'haplotype' to refer to these groups of sites, they are

more precisely described as diplotypes, since the analyses only use unphased genotype data. We called the number of putative archaic haplotypes identified by this approach Nhaps and Dhaps, and the average of these for different populations is given in **Table S10.1**. The motivation for these criteria is that true introgressed regions will generally contain several sites in strong linkage disequilibrium (LD) (Wall 2000), and that conditional on introgressed haplotypes surviving to the present day, they are unlikely to be extremely rare or at high frequency. Tabulating putative archaic haplotypes such as Nhaps reduces the effects of incomplete lineage sorting (which causes some SNPs identified as Nalleles to be ones that were segregating in the population ancestral to Neanderthals and modern humans), but at the cost of greater variance in the estimates since for any sample Nhaps << Nalleles. For individual samples, the allele-based and haplotype-based estimates of Neanderthal and Denisovan ancestry are highly correlated, with r^2 values of 0.831 and 0.988 respectively (**Figure S10.1**). Samples from isolated populations tend to have more putative archaic haplotypes than otherwise expected, perhaps because Nhaps and Dhaps have LD-based definitions and isolated populations have higher background levels of LD. The results described below are for average numbers Nalleles and Dalleles in different populations, though all of the claims are also true for analyses of the number of Nhaps and Dhaps.

We note that the many of these populations have admixed with Austronesian language speaking or other migrants within the last several thousand years(4, 5), and it is likely that the map of Denisovan ancestry proportions has changed substantially over that timescale.

Table S10.1. Estimates of archaic ancestry, averaged for each population with n≥3

Population	Nalleles	Dalleles	Nhaps	Dhaps	N%	D%
**Africa (AFR)**						
BAN	729	601	16.3	13.9	0.05	0.00
LUH	657	381	18.7	9	0.04	0.00
JUH	2760	2697	61.9	65.3	0.45	0.74
DIN	461	362	7	6.8	0.00	0.00
MAS	2364	531	74.3	9.4	0.37	0.00
**West Eurasia (WER)**						
MEJ	9386	635	323	14.3	1.74	0.01
PAL	9194	600	326	15.7	1.71	0.00
JOR	8681	500	284.7	9.3	1.61	0.00
FRE	9782	595	318.8	10.5	1.82	0.00
CEU	10814	654	372.5	12	2.02	0.02
SAR	10875	565	379.3	7.3	2.03	0.00
FIN	10470	630	322	11.3	1.96	0.01
GBR	10699	605	359.4	11.2	2.00	0.00
TSI	10546	675	351	19.3	1.97	0.02
IBS	9586	559	308.7	7.7	1.78	0.00
**South Asia (SAS)**						
JAR	13188	1606	433.5	46	2.49	0.36
ONG	12971	1495	429.3	41.3	2.44	0.32
NIC	12922	1273	362.6	29.2	2.43	0.24
SSI	11445	1304	353.6	33.8	2.15	0.25
ITU	11306	1324	353.6	35.5	2.12	0.26
STU	11144	1428	347.9	44.7	2.09	0.29
BLR	11370	1273	357.9	31.6	2.13	0.24
MAA	11403	1258	358.5	33.6	2.14	0.23
SZH	11395	1222	368.3	34	2.14	0.22
ORN	12787	1566	393.3	44.1	2.41	0.34
BIR	12121	1518	359.4	36.8	2.28	0.32
BHM	11712	1691	351.2	49	2.20	0.39
HKR	12346	1624	379.9	42.7	2.32	0.36
KAM	12360	1673	381.3	51.8	2.32	0.38
LOD	12209	1526	379.5	42.4	2.30	0.33
DHR	12183	1670	372.4	45.1	2.29	0.38

MUR	12367	1657	376.6	45.7	2.15	0.40
ABM	12361	1735	366.5	48.1	2.33	0.40
DOR	12551	1621	387.1	45	2.36	0.36
CHN	11636	1325	366.7	36.1	2.18	0.26
KYD	11258	1557	353.9	47.4	2.11	0.34
PNY	12108	1556	381	44.3	2.28	0.34
KTA	11716	1535	376	46	2.20	0.33
TOD	11333	1268	350.7	34.9	2.12	0.24
IRU	11460	1467	361.5	40.5	2.15	0.31
RTH	13186	1441	379.2	35.3	2.49	0.30
HLB	11942	1678	380.5	47.5	2.24	0.38
LAM	11155	1237	351.7	30.6	2.09	0.22
CHK	13087	1366	374.3	38.1	2.47	0.27
JAM	13051	1355	374.2	35.4	2.46	0.27
MOG	13048	1405	384.1	36.9	2.46	0.28
TTO	13003	1429	378.4	35.6	2.45	0.29
KHA	11205	994	363.5	21.5	2.10	0.14
GAU	11987	1479	365.8	48	2.25	0.31
SRB	10983	1121	352	29.5	2.06	0.18
SOB	10490	1077	365.8	33.4	1.96	0.17
WBB	10782	1041	371.8	25.8	2.02	0.15
IYE	11416	1209	367	33.3	2.14	0.21
IYA	11180	1235	363.2	33	2.09	0.22
CHM	11044	1313	347	37.8	2.07	0.25
PJB	11103	1180	357.6	27.4	2.08	0.20
RAJ	11325	1151	359.4	30.1	2.12	0.19
MHR	11644	1314	360.3	33.1	2.18	0.25
NAB	11790	1388	381.3	37.8	2.21	0.28
KNR	12291	1622	373.8	45.9	2.31	0.36
AGH	10854	1211	364.9	33.5	2.03	0.22
BAG	10585	1501	349.7	46.3	1.98	0.32
MNP	11378	1192	390.4	37.4	2.13	0.21
PAT	10647	929	337.9	23	1.99	0.12
BRU	11044	953	350.1	20.1	2.07	0.12
GUJ	10899	1044	343.6	26.3	2.04	0.16
HAZ	11933	992	370.9	25.2	2.24	0.14
SND	10157	985	333.1	21.8	1.89	0.13
BRA	10255	827	333.3	18.5	1.91	0.08

Southeast Asia (SEA)

ATI	13550	4187	392.2	117.1	2.56	1.27
AET	13948	10707	423.3	313.6	2.64	3.59
KEN	13218	1534	383.9	42.3	2.49	0.33
SNS	13077	1374	378.9	34.8	2.47	0.27
TEM	13040	1295	382.8	32	2.46	0.25
KIN	13362	1722	379.4	51.5	2.52	0.40
MLY	12870	1592	371.3	42.7	2.42	0.35
DAI	13263	1193	374	30.7	2.50	0.21
KHV	13307	1254	372.4	32.9	2.51	0.23
MEN	13333	1313	396.3	30.6	2.52	0.25
NIA	13373	1286	379.1	29.1	2.52	0.24
BEN	14081	4757	413.5	137.1	2.66	1.47
CIB	13729	3907	412.7	120	2.59	1.17
RAM	13400	3950	390.2	118.3	2.53	1.19
**Northeast Asia (NEA)**						
HAN	13463	1257	377.6	33.2	2.54	0.23
KHL	13559	1181	403.3	26.4	2.56	0.20
JPN	13399	1242	385.1	33.8	2.53	0.23
KOR	13390	1247	377.7	32.8	2.53	0.23
BUR	13146	1234	381.9	31	2.48	0.22
MNG	13331	1200	376	35.7	2.51	0.21
EVN	13378	1326	431	37.7	2.52	0.26
ESK	13903	1207	413.8	30	2.63	0.21
**Oceania (OCE)**						
BAI	14843	11211	454.7	351.9	2.81	3.77
PAP	14900	11869	456.9	375.1	2.82	4.00
NAK	14286	9131	429	265	2.70	3.03
NKB	14522	8817	450.5	272.5	2.75	2.92
**Americas (AMR)**						
MXE	12947	1203	395	35.3	2.44	0.21
KAR	12673	1099	354.3	25.3	2.39	0.18
QUE	13234	1150	401	31.7	2.50	0.19

Figure S10.1. Scatterplots of the numbers of (**a**) Dhaps vs. Dalleles and (**b**) Nhaps vs. Nalleles for each individual.

a

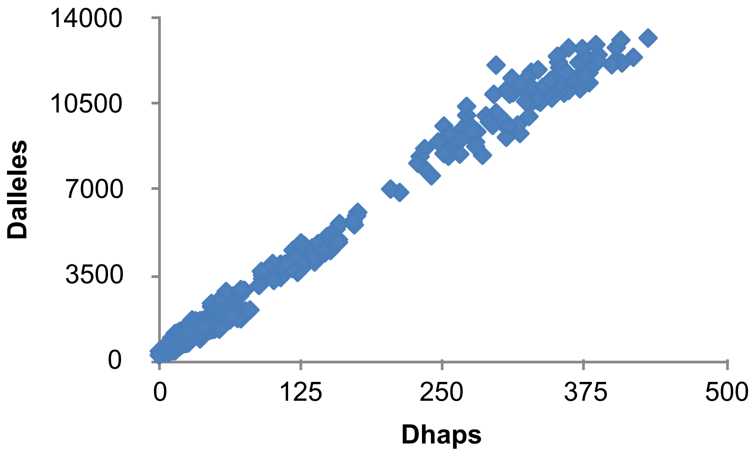

b

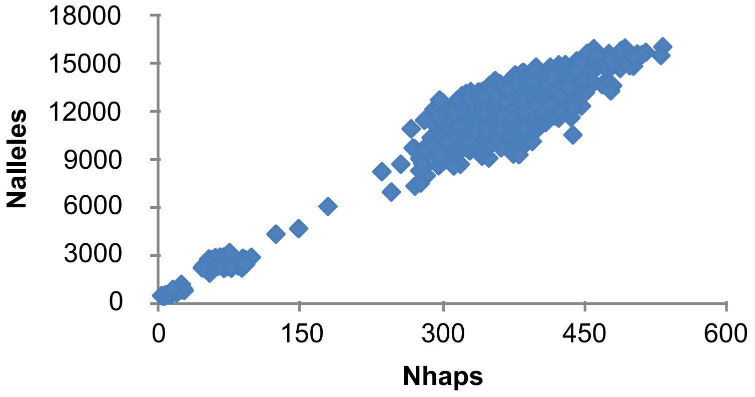

Overall, we found the highest amounts of Neanderthal admixture in Papuan and Melanesian genomes, followed by East and Southeast Asian genomes, South Asian genomes, European genomes and Middle Eastern genomes (**Figure S10.2**). These results are qualitatively similar to those of several previous studies (e.g., Malaspinas et al. 2016, S10; Mallick et al. 2016; Sankararaman et al. 2016). For completeness, we performed the same calculations for sub-Saharan African groups not used in the initial screening process described above. If we assume these individuals do not have any Neanderthal ancestry, then the Nalleles and Nhaps values for the Dinka, Bantu and Luhya give us a qualitative idea of the amount of false positives due to incomplete lineage sorting in the Neanderthal – modern human ancestor. Note that the higher estimate of Neanderthal ancestry in the Maasai is likely due to recent admixture with

non-African groups (Wall et al. 2013; Pickrell et al. 2014), while the higher estimate of Neanderthal ancestry in the Juhoansi is because their older divergence time with West and Central Africans leads to more incomplete lineage sorting (Mallick et al. 2016).

Our estimates of Denisovan admixture across populations also recapitulate the broad-scale results of previous studies. However, the larger number of samples and populations in our study allows us to obtain a more nuanced understanding of the global patterns of Denisovan admixture. We reexamined the pattern of greater Denisovan admixture than expected in South Asian populations, first noted by Sankararaman et al. (2016). For this analysis, we subdivided the South Asian samples into four groups, consisting of Pakistani samples, high caste individuals, other caste individuals and Tribal (Adivasi) individuals. We further subdivided each group into Indo-European language speakers and non-Indo-European language speakers. Our analysis excluded Tibeto-Burman speakers from Northeast India since they cluster genetically with Southeast Asian populations. They also excluded enigmatic non-indo-European Pakistani groups (Burusho and Brahui).

Figure S10.2. Heatmap of estimated levels of Neanderthal (A) and Denisovan (B) ancestry as a function of country of origin.

a

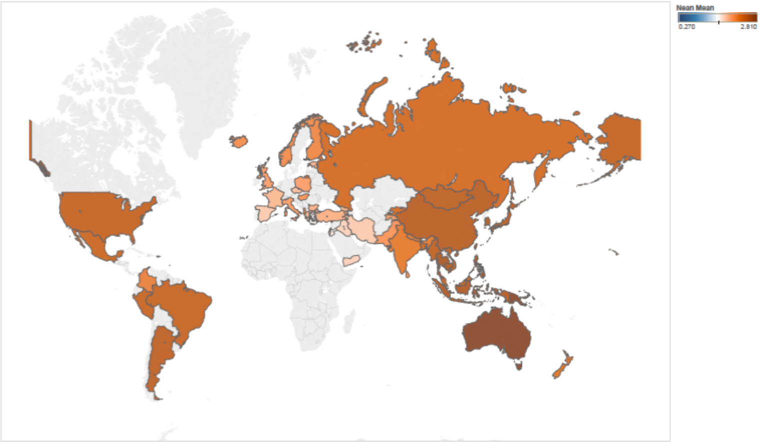

b

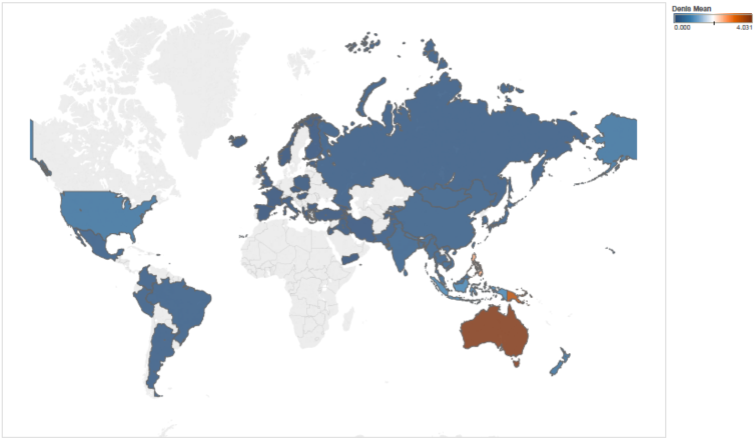

We found a highly significant gradient of Denisovan admixture across groups, with Tribal individuals having more Denisovan ancestry than caste individuals (0.33% vs. 0.25%, $p<<10^{-5}$, Mann-Whitney U test), who have more Denisovan ancestry than high caste individuals (0.25% vs. 0.19%, $p<<10^{-5}$, Mann-Whitney U test), who have more Denisovan ancestry than Pakistani individuals (0.19% vs. 0.13%, $p<<10^{-5}$, Mann-Whitney U test) (**Table S10.2**). Within the 3 Indian groups, non-Indo-European language speakers had significantly more Denisovan ancestry than did Indo-European language speakers, while the situation was reversed in the Pakistani populations. There are a handful of exceptions to this pattern though – the Toda, a Dravidian-language-speaking tribal group do not have elevated Denisovan ancestry, while the Halba (an Indo-European-language speaking tribal group) and the KondaReddy (a South Indian caste group) both do have higher levels of Denisovan ancestry. In absolute terms though the range of Denisovan ancestry proportions in South Asian populations is small (0.1-0.4%).

We note as a caveat that our use of a Mann-Whitney U test for the comparison between populations implicitly assumes that the timing of population splits (within South Asia) are much more recent than the time of Denisovan admixture. If these times were roughly contemporaneous, then genetic drift alone could lead to systematic differences in estimated introgression levels between populations (i.e., each individual's admixture estimate should not be considered as an independent data point). While our assumption is reasonable for South Asian comparisons (where population split times are on the order of thousands of years ago), it would be difficult to make a similar comparison between the Aeta and Papuans, for example.

Our results are broadly consistent with a model where indigenous South Asian groups had elevated levels of Denisovan ancestry (as still found in the Tribal groups), but that immigration from the North and West (primarily of Indo-Aryan groups) diluted this Denisovan ancestry to various degrees in the caste and Pakistani populations included in our study.

Our dense sampling also allowed us to examine the patterns of Denisovan admixture into so-called ‘Negrito’ populations. We theorized that if these groups shared recent common ancestry with Melanesian populations, then they should show elevated levels of Denisovan admixture, as do Melanesian and Papuan groups. We found that the Philippine Negrito groups (Aeta and Ati) do have elevated levels of Denisovan ancestry (3.6% and 1.3% respectively), while the Malay Negrito (Kensiu and Kintak) and Andaman Negrito (Jarawa, Onge and Nicobarese) populations do not (all <0.5%). This is consistent with a model where the Ati have experienced a substantial amount of recent admixture with other groups in the Philippines. We note in passing that though the populations studied here are mostly different from those studied previously, our general observation of substantial Denisovan ancestry in Philippine Negritos but not in Malay or Andaman Negritos is the same as what was found in a previous array-based study (Reich et al. 2011).

Table S10.2. 2-sided Mann-Whitney U test results for comparisons between different South Asian subgroups. The z-score uses a normal approximation for large sample sizes. Average percent estimates of Denisovan ancestry are given in parentheses next to the group names, and all p-values are 2-tailed. Sample sizes are as follows: Adivasi IE: n=30; Adivasi non-IE: n=196; Caste IE: n=68; Caste non-IE: N=155; Upper IE: n=49; Upper non-IE: n=19; Pakistani IE: n=79.

Comparison	U-value	z-score	p-value
All samples			
Adivasi (0.33%) vs. Caste (0.25%)	11814	-9.586	<< 10^{-9}
Caste (0.25%) vs. Upper (0.19%)	3733.5	-6.241	< 10^{-9}
Upper (0.19%) vs. Pakistani (0.13%)	1396.5	-5.840	~$2.6*10^{-9}$
Indo-European language speakers			
Adivasi (0.29%) vs. Caste (0.23%)	630.5	-2.754	0.00596

Caste (0.23%) vs. Upper (0.18%)	759	-4.768	< 10^{-5}
Upper (0.18%) vs. Pakistani (0.13%)	1206	-3.574	0.00036
<u>Non Indo-European language speakers</u>			
Adivasi (0.34%) vs. Caste (0.26%)	7227.5	-8.434	<< 10^{-5}
Caste (0.26%) vs. Upper (0.22%)	966.5	-2.439	0.01468
<u>IE vs. non-IE language speakers</u>			
Adivasi non-IE vs. Adivasi IE	1918	-3.063	0.00222
Caste non-IE vs. Caste IE	4366	-1.558	0.11876
Upper non-IE vs. Upper IE	271.5	-2.645	0.0083

References

Malaspinas AS, Westaway MC, Muller C, Sousa VC, Lao O, et al. (2016) A genomic history of Aboriginal Australia. Nature 538: 207-214.

Mallick S, Li H, Lipson M, Mathieson I, Gymrek M, et al. (2016) The Simons Genome Diversity Project: 300 genomes from 142 diverse populations. Nature 538: 201-206.

Pickrell JK, Patterson N, Loh PR, Lipson M, Berger B, et al. (2014) Ancient west Eurasian ancestry in southern and eastern Africa. Proc Natl Acad Sci USA 111: 2632-2637.

Prufer K, Racimo F, Patterson N, Jay F. Sankararaman S, et al. (2014) The complete genome sequence of a Neanderthal from the Altai Mountains. Nature 505: 43-49.

Reich D, Patterson N, Kircher M, Delfin F, Nandineni MR, et al. (2011) Denisova admixture and the first modern human dispersals into Southeast Asia and Oceania. Am J Hum Genet 89: 516-528.

Sankararaman S, Mallick S, Patterson N, Reich D (2016) The combined landscape of Denisovan and Neanderthal ancestry in present-day humans. Curr Biol 26: 1241-1247.
Wall JD (2000) Detecting ancient admixture in humans using sequence polymorphism

data. Genetics 154: 1271-1279.

Wall JD, Yang MA, Jay F, Kim SK, Durand EY, et al. (2013) Higher levels of Neanderthal ancestry in East Asians than in Europeans. Genetics 194: 199-209.

Supplementary note S11 – Identification of disease-causing variants in GAsP dataset

Ravi Gupta[1], Vedam Ramprasad[1], Sivasankar Malaichamy[1], Sandhya Nair[1], Sameer Phalke[1], Joyner T. George[1], Thiramsetti Sattibabu[1], Vivek Gopalan[1], Somasekar Seshagiri[2]

[1]MedGenome Labs Pvt. Ltd., Bengaluru, Karnataka 560099, India; [2]Dept. of Molecular Biology, Genentech Inc., South San Francisco, CA 94080

author responsible for this section: Ravi Gupta - ravig@medgenome.com

Results

The ~70.2 million variants present in GenomeAsia pilot dataset was annotated against various databases/tools using MedGenome deep annotation tool (VariMAT). After annotation, following steps were followed to identify disease-causing variants in the dataset.

a. **Identification of disease-causing variants** – The variants annotated as disease casing in HGMD Professional and ClinVar databases were first identified. Then, the variants annotated as exonic and splice-site variants were retained. This resulted in 732 variants.

b. **Filtration of common polymorphism** – The filtered variants allele frequency in different population databases 1000 genomes, ExAC, dbSNP, ALSPAC, TWIN-SUK and 1000 Japanese databases was extracted from the annotation file. Then max allele frequency for the variants in these databases was calculated. The variants for which the max allele frequency is < 1% was retained. We obtained 615 variants.

c. **Filtration of common polymorphism in GenomeAsia cohort** – The variant allele frequency of these 615 variants in GenomeAsia cohort was evaluated. We found only one variant for which allele frequency >= 1%. At the end, we obtained 614 variants (SNP – 575, Indel – 39) mapping to 458 genes.

d. **Re-classification of variants using manual curation** – The disease-causing variants were shifted through the literatures mentioned in HGMD, ClinVar, SwissVar databases. The zygosity/inheritance pattern, variant class and functional aspects of the variants were evaluated. Based on the available evidences the variants were re-classified using ACMG guidelines (2015) to ensure that there was enough evidence to call these variants as pathogenic or VUS or benign unequivocally.

American College of Medical Genetics (ACMG) guidelines focus on diseases and variants common in those of European descent. To identify common diagnostically sequenced disease-associated variants that may be more common or found exclusively in Asian populations, we focused on predicted damaging variants found in 124 genes commonly used for prenatal and cancer screening (see Methods). We found 26 disease-associated genes with variants significantly over-represented or exclusive to Asian populations (**Supplementary Table S4a**). The biggest cumulative difference in relative allele frequency (GAsP allele frequency minus the gnomAD non-Asian allele frequency) was found in alleles whose genes are linked to Usher syndrome, NEB-related nemaline myopathy and Familial Mediterranean Fever. Amongst the alleles that we identified in this virtual carrier screening is the A44G mutation in G6PD, previously reported to be found exclusively in tribes in India and linked to glucose-6-phosphate dehydrogenase deficiency(6).

Figure S11.1. Workflow for identification of disease-causing variants. The workflow for identifying the disease-causing variants in the Genome Asia pilot dataset is shown in the figure. First the variants present in our dataset and present as disease-causing variants in Human Gene Mutation Database (HGMD) and ClinVar were identified. Then, the variants that are common (>= 0.01 allele frequency) in any one of the following databases: 1000K phase3, ExAC, dbSNP, ALSPAC, TWIN-SUK, 1000Japanese, were removed. In the last step, we remove variants that are common (>= 0.01 allele frequency) in the Genome Asia pilot dataset.

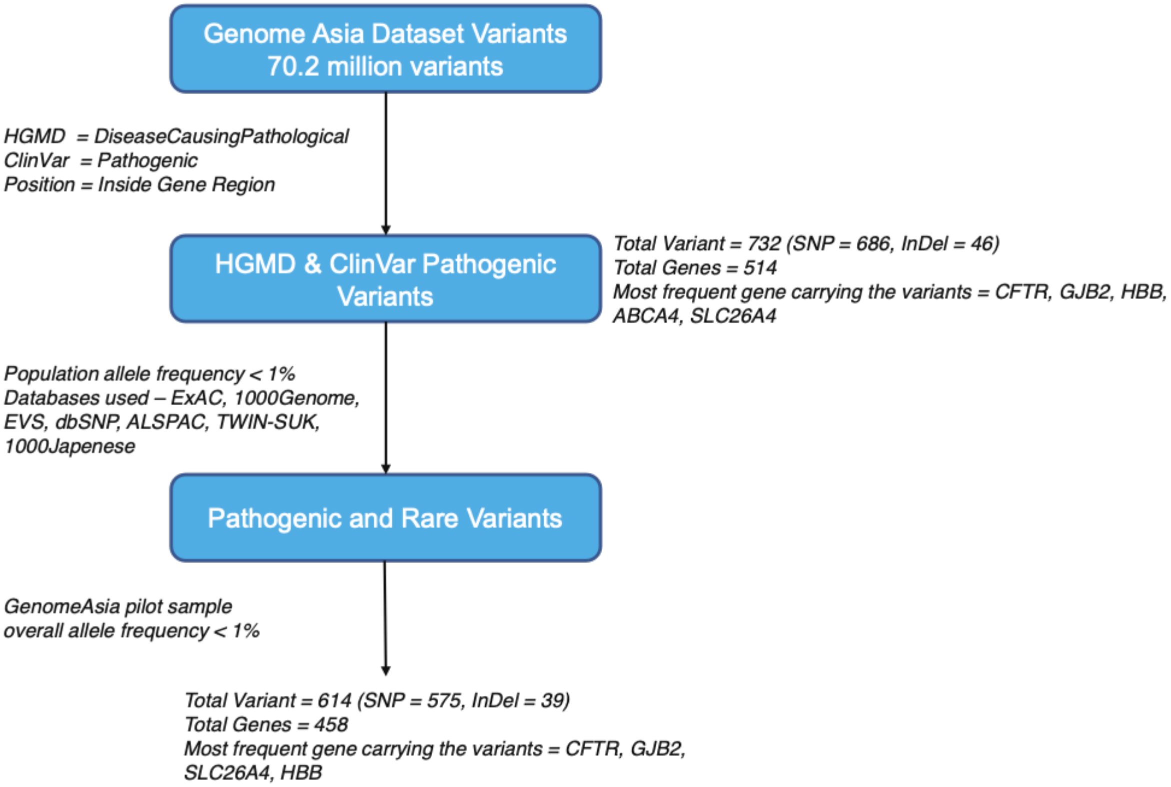

Figure S11.2. Variant class distribution for the disease-causing variants. The 614 disease-causing variants were annotated against the genes. Of the total, 69% are of missense type, 5% affect the splicing site and 19% are of loss of function (Nonsense and Frameshift) type.

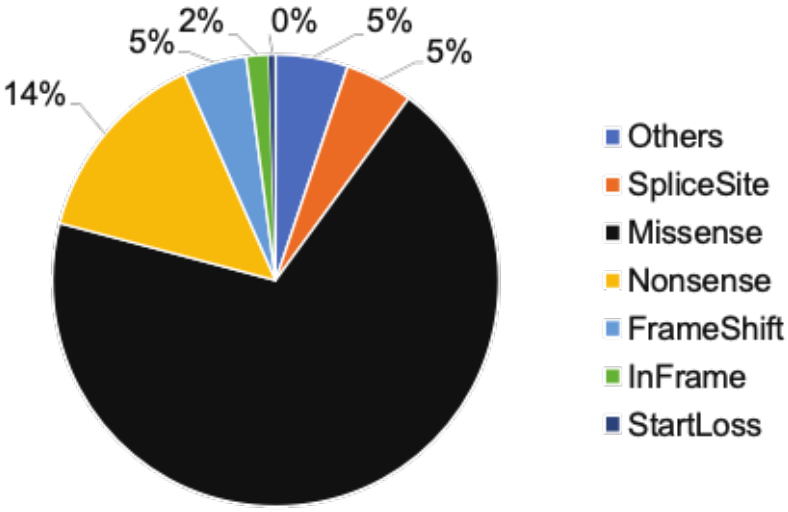

Figure S11.3. Genes with disease-causing variants. Genes with > 2 unique disease-causing variants in the Genome Asia pilot dataset are shown in the figure. The top genes with highest number of disease-causing variants include GJB2, CFTR, SLC26A4, HBB, ABCA4.

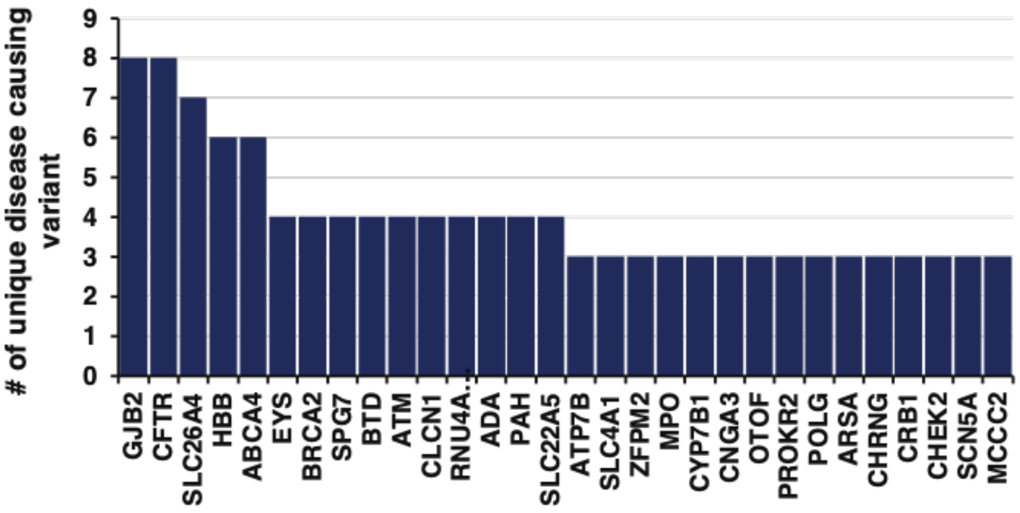

Supplementary note S12 – Identity by descent analysis

Anjali Verma[1], Ramesh Menon[1], Andrew Peterson[2] and Ravi Gupa[1]

[1]MedGenome Labs Pvt. Ltd., Bengaluru, Karnataka 560099, India; [2]Department of Molecular Biology, Genentech Inc., 1 DNA Way, South San Francisco, CA 94080

author responsible for this section - Ravi Gupta – ravig@medgenome.com

Method

IBD analysis. We first phased the whole genome using Shapeit version 2 program[7] with settings window size 0.5. The related samples (**Supplementary Information S1**) were discarded before phasing. After this, the variants which fall within the high-confident regions of the human genome (hg19) provided by Genome In A Bottle – GIAB consortium (www.jimb.stanford.edu/giab/) (version 3.3.2) and biallelic SNVs were retained for the analysis. A total of 20.34 million SNVs were obtained for our downstream analysis. We then performed QC step to remove the related samples. We first performed the Kinship analysis using PLINK v1.9 with the parameter phi-hat cutoff = 0.45[8]. No related samples were identified using this step. We then used SNPrelate v1.14 program[9] with default settings for the samples in each region separately. We then removed the samples for which the first three principal components values match exactly up to three decimal points. This step identified 30 related samples (**Supplementary Information S1**). The IBD segments between sample pair in the group were identified using GERMLINE tool v1.5.3[10] with parameter *-bits 75 -err_hom 0 -err_het 0 -min_m 3*. Eight samples with at least one IBD segment size > 30cM were removed from further analysis (**Supplementary Information S1**). The IBD segments with genetic lengths from 3 to 20 cM obtained using GERMLINE were retained. The IBD segments were further filtered using the Haploscore program 2014 release[6] with settings mean overlap 0.8. The IBD score for each sample pair in the ethnic group and using the method described by Nakatsuka et. al.[6,11] we generated the IBD score for the ethnic groups.

IBD Data Analysis

Shared segment based analysis is a powerful technique to map the disease-causing genes for inherited disorders[1–3]. IBD analysis can be more powerful than association analysis for complex diseases, especially when several disease-causing rare variants are clustered together[1,4]. IBD is also applied in understanding the relatedness of the samples and can predict up to 6-7 generation of relatedness with a good accuracy[5]. With the help of IBD the linkage analysis can

be performed without pedigree. A recent study on South Asia population groups has identified 14 groups that has an estimated population size of more than 1 million[6]. Some of these groups to known to carry a higher burden for various recessive disorders.

We followed the approach as described in a previous study on South Asian population for our IBD analysis[6]. We restricted our data to the high confident callable region and removed the related samples based on known information, Kingship, PCA and IBD analysis (see the method section for details, **Supplementary Information S1**). The IBD was computed between each sample pair in the group and also for each group with >= 3 samples. Overall, we report IBD score of 96 different ethnic/population groups (with at least 3 non-related samples) covering 1,417 samples, 27 countries and 7 regions (SAS, SEA, AMR, OCE, NEA, WER, AFR) of the globe (**Supplementary Information S2**).

Our analysis report IBD score of 1.465, 0.817 for Finnish (FIN) and Great Britain (GBR) group respectively and is on the expected line[6]. The IBD score of the groups was normalized relative to Finnish group and is shown in **Figure 1a** (**Supplementary Information S2**). Our analysis revealed 37 groups with an IBD score greater than Finnish group. Of these, 18 groups (ONG, JAR, TTO, TOD, NIC, BIR, CHN, KAM, SZH, LOD, ABM, DHR, MAA, MUR, HKR, BHM, MOG, JAM) belong to India, 4 groups belong to Malaysia (KIN, SNS, KEN, TEM), 3 groups belong to Papua New Guinea (BAI, NAK, NKB), 2 groups each belong to Indonesia (MEN, BEN), Philippines (AET, ATI), Russia (ESK, EVN), Italy (SAR, TSI) and 1 group each from Mexico (MXE), Pakistan (BRU), Peru (QUE) and Mongolia/China (MNG). As expected, we observed higher IBD score for the tribal as compared to non-tribal groups (**Figure 1b**). Of all the 19 SAS groups which have higher IBD score than Finnish, 15 (78.95%) groups belong to tribal category (**Table 1**). Of these, Onge, Nicobarese, Birhor and Burusho groups have been also reported to have higher IBD than Finns in a previous study[6]. Interestingly, we observe the South Indian and Urban Chennai groups that represents population size of several million samples have higher IBD than Finns. Other interesting groups includes Lodha and Abhuj Maria groups which are tribal.

Reference

1. Browning, S. R. & Thompson, E. A. Detecting Rare Variant Associations by Identity-by-Descent Mapping in Case-Control Studies. *Genetics* **190**, 1521–1531 (2012).

2. Te Meerman, G. J., Van der Meulen, M. A. & Sandkuijl, L. A. Perspectives of identity by descent (IBD) mapping in founder populations. *Clin. Exp. Allergy* **25 Suppl 2**, 97–102 (1995).

3. Moltke, I., Albrechtsen, A., Hansen, T. V. O., Nielsen, F. C. & Nielsen, R. A method for detecting IBD regions simultaneously in multiple individuals--with applications to disease genetics. *Genome Res.* **21**, 1168–1180 (2011).

4. Evans, L. M. *et al.* Narrow-sense heritability estimation of complex traits using identity-by-descent information. *Heredity (Edinb)* (2018). doi:10.1038/s41437-018-0067-0

5. Browning, S. R. & Browning, B. L. Identity by descent between distant relatives: detection and applications. *Annu. Rev. Genet.* **46**, 617–633 (2012).

6. Nakatsuka, N. *et al.* The promise of discovering population-specific disease-associated genes in South Asia. *Nat. Genet.* **49**, 1403–1407 (2017).

7. O'Connell, J. *et al.* A general approach for haplotype phasing across the full spectrum of relatedness. *PLoS Genet.* **10**, e1004234 (2014).

8. Purcell, S. *et al.* PLINK: a tool set for whole-genome association and population-based linkage analyses. *Am. J. Hum. Genet.* **81**, 559–575 (2007).

9. Zheng, X. *et al.* A high-performance computing toolset for relatedness and principal component analysis of SNP data. *Bioinformatics* **28**, 3326–3328 (2012).

10. Gusev, A. *et al.* Whole population, genome-wide mapping of hidden relatedness. *Genome Res.* **19**, 318–326 (2009).

11. Durand, E. Y., Eriksson, N. & McLean, C. Y. Reducing pervasive false-positive identical-by-descent segments detected by large-scale pedigree analysis. *Mol. Biol. Evol.* **31**, 2212–2222 (2014).

Supplementary note S13 – Allele frequencies of key pharmacogene variants

Kushal Suryamohan[1], Steffen Durinck[1,2] , Ramesh Menon[3], Anjali Verma[3], Ravi Gupa[3] and Somasekar Seshagiri[1]

[1]Department of Molecular Biology, Genentech Inc., 1 DNA Way, South San Francisco, CA 94080; [2]Department of Bioinformatics and Computational Biology, Genentech Inc., 1 DNA Way, South San Francisco, CA 94080. [1]MedGenome Labs Pvt. Ltd., Bengaluru, Karnataka 560099, India.

author responsible for this section – Kushal Suryamohan, suryamohan.kushal@gene.com, Ravi Gupta – ravig@medgenome.com and Somasekar Seshagiri – sekar@gene.com

Using published dosing guidelines for several pharmacogenes(7), we estimated the proportion of each population group predicted to be susceptible to adverse drug responses (ADR), focusing on eight drugs where genetic information should be used to change prescribing behavior (Clinical Pharmacogenetics Implementation Consortium (https://cpicpgx.org)).

Allele frequencies of three key pharmacogene variants from our data set (Supplementary Table S13.1) were plotted as heatmaps (Figure S13.1) revealing differences in SNP frequency between population groups. The HLA-B *15:02 variant, associated with risk for development of Steven Johnson Syndrome (SJS) (Roujeau and Stern, 1994; Tangamornsuksan et. al. 2013) occurs at an elevated frequency in certain Southeast Asian populations (63% in the Mentawai of West Sumatra; 46.6% in the Nias of North Sumatra) compared to other groups.

Similarly, *Cyp2c19* variants also show hotspots in our study cohort with high frequency of the *Cyp2c19 *2* variant allele in Oceania populations (97% in the Baining and 50% in the Nakanei Bileki of Papua New Guinea) and a subset of South Asian populations (68% in the Lodha of eastern India; 57% of the Birhor people in eastern India; 55% in the Kamar of central and eastern India; 58% in the Rana Tharu of north India).

The VKORC1 -1639G>A variant, commonly implicated in adverse drug responses to Warfarin (Johnson JA, et. al. 2017), was found in 88% in Buryats of

Mongolia, 100% of the Han Chinese samples in Northeast Asia, while ~92% of Indonesians of Austroindonesian descent and 93% in Kinh Vietnamese samples from Southeast Asia had this variant allele. These findings highlight the need for appropriate pharmacogenomics testing before drug treatment.

Figure S13.1: Pharmacogene variant alleles show hotspots in certain ethnic populations. Frequencies of key variant alleles (indicated by the variant allele's rsID) within each ethnic group are plotted as a heatmap. Refer to Supplemental table SX for additional genes and the frequencies of variants associated with ADRs*. HLA-B*, Human

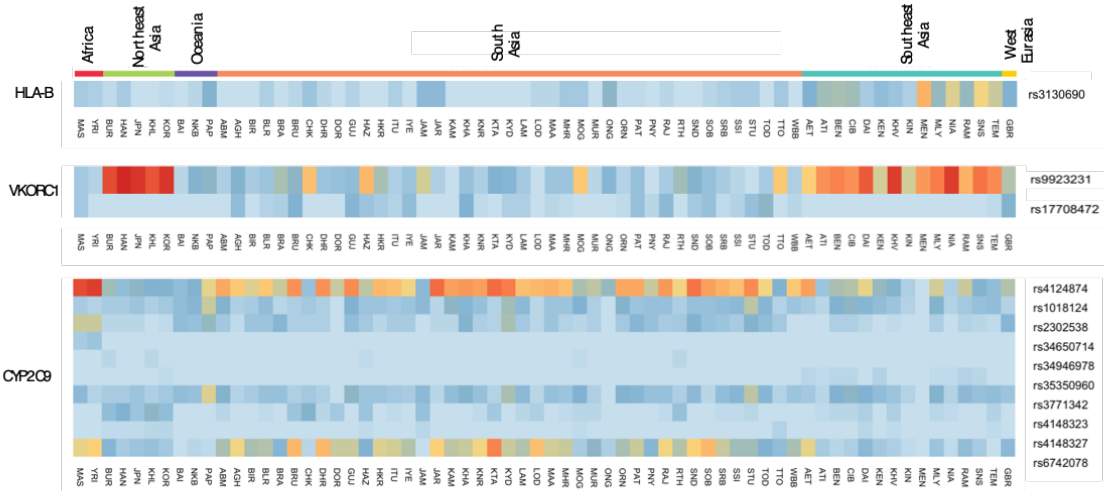

Leukocyte Antigen-B; *VKORC1*, Vitamin K epOxide Reductase Complex; *CYP2C9*, cytochrome P450 family 2 subfamily C member 9

Figure S13.2. Proportion of heterozygous and homozygous variants in the 8 clinically actionable PGx markers across 7 major population group. Population specific proportions of homozygous and heterozygous variants for each drug-gene interaction, and predicted changes in drug dosage, efficacy, metabolism, and toxicity. For each drug indicated in red, variants for associated genes (cyan box) are indicated in blue boxes.

Bibliography

1. Tom, J.A., et al., Identifying and mitigating batch effects in whole genome sequencing data. BMC Bioinformatics, 2017. **18**(1): p. 351.

2. 1000 Genomes Project Consortium, et al., A global reference for human genetic variation. Nature, 2015. **526**(7571): p. 68-74.

3. Wall, J.D., et al., Estimating genotype error rates from high-coverage next-generation sequence data. Genome Res, 2014. **24**(11): p. 1734-9.

4. Diamond, J.M., Express train to Polynesia. Nature, 1988. **336**: p. 307-308.

5. Mijares, A.S.B., The early Austronesian migration to Luzon: perspectives from the Penablanca cave sites. Bulletin of the Indo-Pacific Prehistory Association, 2006. **26**: p. 72-78.

6. Kaeda, J.S., et al., A new glucose-6-phosphate dehydrogenase variant, G6PD Orissa (44 Ala-->Gly), is the major polymorphic variant in tribal populations in India. Am J Hum Genet, 1995. **57**(6): p. 1335-41.

7. Relling, M.V. and T.E. Klein, CPIC: Clinical Pharmacogenetics Implementation Consortium of the Pharmacogenomics Research Network. Clin Pharmacol Ther, 2011. **89**(3): p. 464-7.
