# Supplementary material for: The GenomeAsia 100K Project enables genetic discoveries across Asia
Source: Nature. 2019 Dec 4;576(7785):106–11. doi: 10.1038/s41586-019-1793-z (PMC7054211; doi:10.1038/s41586-019-1793-z)
Supplement: Supplementary file 2 — Reporting Summary [file 41586_2019_1793_MOESM2_ESM.pdf]

nature research

Corresponding author(s): Andrew S. Peterson
Last updated by author(s): Aug 10, 2019

# Reporting Summary

Nature Research wishes to improve the reproducibility of the work that we publish. This form provides structure for consistency and transparency in reporting. For further information on Nature Research policies, see Authors & Referees and the Editorial Policy Checklist.

## Statistics

For all statistical analyses, confirm that the following items are present in the figure legend, table legend, main text, or Methods section.

| n/a | Confirmed | |
|---|---|---|
| ☐ | ☒ | The exact sample size (*n*) for each experimental group/condition, given as a discrete number and unit of measurement |
| ☐ | ☒ | A statement on whether measurements were taken from distinct samples or whether the same sample was measured repeatedly |
| ☐ | ☒ | The statistical test(s) used AND whether they are one- or two-sided<br>*Only common tests should be described solely by name; describe more complex techniques in the Methods section.* |
| ☐ | ☒ | A description of all covariates tested |
| ☐ | ☒ | A description of any assumptions or corrections, such as tests of normality and adjustment for multiple comparisons |
| ☐ | ☒ | A full description of the statistical parameters including central tendency (e.g. means) or other basic estimates (e.g. regression coefficient) AND variation (e.g. standard deviation) or associated estimates of uncertainty (e.g. confidence intervals) |
| ☐ | ☒ | For null hypothesis testing, the test statistic (e.g. *F*, *t*, *r*) with confidence intervals, effect sizes, degrees of freedom and *P* value noted<br>*Give P values as exact values whenever suitable.* |
| ☐ | ☒ | For Bayesian analysis, information on the choice of priors and Markov chain Monte Carlo settings |
| ☐ | ☒ | For hierarchical and complex designs, identification of the appropriate level for tests and full reporting of outcomes |
| ☐ | ☒ | Estimates of effect sizes (e.g. Cohen's *d*, Pearson's *r*), indicating how they were calculated |

*Our web collection on statistics for biologists contains articles on many of the points above.*

## Software and code

Policy information about availability of computer code

| | |
|---|---|
| Data collection | no software was used |
| Data analysis | BWA version 0.7.13 (https://github.com/lh3/bwa);<br>SAMBLASTER version 0.1.22 (https://github.com/GregoryFaust/samblaster) Sambamba version 0.6.1 (https://github.com/lomereiter/sambamba) BAMreport version 0.0.2; (https://github.com/aakrosh/BAMreport) verifyBamID version 1.1.3 (http://genome.sph.umich.edu/wiki/VerifyBamID); GATK version 3.5 (https://software.broadinstitute.org/gatk/);<br>vcfanno version 0.1.0-dev (https://github.com/brentp/vcfanno);<br>htslib version 1.3.1-64-g74bcfd7 (https://github.com/samtools/htslib); vcftools version 0.1.14 (https://vcftools.github.io/index.html);<br>plink version 1.90b3.40 (http://zzz.bwh.harvard.edu/plink/); king version 1.4 (http://people.virginia.edu/~wc9c/KING/); rtg-tools version 3.7 (https://github.com/RealTimeGenomics/rtg-tools);<br>Shapeit v2 (Delaneau et al, 2012);<br>ex- tractPIRs (Delaneau et al, 2013);<br>Eagle2 algorithm (Loh et al. 2016), version 2.3;<br>generate_multihetsep.py, downloaded from https:// github.com/stschiff/msmc-tools;<br>Admixture v.1.3.0 (Alexander et al, 2009);<br>EIGENSTRAT v.6.1.4 (Price et al, 2006);<br>Selscan v. 1.1.0 (Szpiech and Hernandez 2014);<br>BEAST v.1.8.4 (Drummond et al. 2012);<br>PLINK v1.9 |

For manuscripts utilizing custom algorithms or software that are central to the research but not yet described in published literature, software must be made available to editors/reviewers. We strongly encourage code deposition in a community repository (e.g. GitHub). See the Nature Research guidelines for submitting code & software for further information.

# Data

Policy information about availability of data

All manuscripts must include a data availability statement. This statement should provide the following information, where applicable:

- Accession codes, unique identifiers, or web links for publicly available datasets
- A list of figures that have associated raw data
- A description of any restrictions on data availability

For each variant, summary data for genotype quality, allele depth and population specific allele counts were calculated before removing all genotype data. This data set is available without requirement for login or other form of restriction for browsing or for download at (https://browser.genomeasia100k.org). Individual level VCF data files representing 1,180 newly sequenced genomes from individuals in 74 population groups are freely available to any qualified investigator without restriction. Chinese samples sequenced were from Corriell cell lines and are not subject to the Chinese regulation. The data are available from the European Genome Archive (EGA) under accession number EGAS00001002921.

The procedure for accessing individual level data is as follows:
Access forms obtained from the GenomeAsia website (https://browser.genomeasia100k.org), once filled out and returned to dataaccess@genomeasia100k.org will undergo administrative review and instructions for download will be returned to the requestor. Access to individual level data from Malaysian samples are subject to additional restrictions.
The complete data set of sequences of unrelated individuals (1,667 samples) has been phased and can be used for imputation through the Michigan Imputation Server (https://imputationserver.sph.umich.edu/index.html)
The goal of the GenomeAsia100K consortium is to facilitate and accelerate genetic studies in Asian populations by coordinating sequencing efforts amongst its members. To achieve this goal we are committed to continuing to make data publicly available and accessible. As data is contributed to the consortium by individual members it will be made immediately available in summary form or as imputation reference panels where appropriate. Data will be made available in individual form wherever possible and not limited by the bounds of informed consent, national privacy laws and regulations, or other external restrictions that may apply.

# Field-specific reporting

Please select the one below that is the best fit for your research. If you are not sure, read the appropriate sections before making your selection.

☒ Life sciences ☐ Behavioural & social sciences ☐ Ecological, evolutionary & environmental sciences

For a reference copy of the document with all sections, see nature.com/documents/nr-reporting-summary-flat.pdf

# Life sciences study design

All studies must disclose on these points even when the disclosure is negative.

| | |
|---|---|
| Sample size | No statistical methods were used to predetermine sample size. and the investigators were not blinded to the allocation during analysis. |
| Data exclusions | data was not excluded unless it failed essential QC metrics |
| Replication | results were not externally replicated |
| Randomization | The experiments were not randomized. |
| Blinding | Investigators were not blinded to the allocation during analysis. |

# Reporting for specific materials, systems and methods

We require information from authors about some types of materials, experimental systems and methods used in many studies. Here, indicate whether each material, system or method listed is relevant to your study. If you are not sure if a list item applies to your research, read the appropriate section before selecting a response.

## Materials & experimental systems

| n/a | Involved in the study |
|---|---|
| ☒ | ☐ Antibodies |
| ☒ | ☐ Eukaryotic cell lines |
| ☒ | ☐ Palaeontology |
| ☒ | ☐ Animals and other organisms |
| ☐ | ☒ Human research participants |
| ☒ | ☐ Clinical data |

## Methods

| n/a | Involved in the study |
|---|---|
| ☒ | ☐ ChIP-seq |
| ☒ | ☐ Flow cytometry |
| ☒ | ☐ MRI-based neuroimaging |

## Human research participants

Policy information about studies involving human research participants

| | |
|---|---|
| Population characteristics | 200 populations groups were included in our study and study participants included equal numbers of both genders |
| Recruitment | participants were recruited based on self and external identification as member of a specific population groups |
| Ethics oversight | Nanyang Technological University institutional review board (IRB- 2014-12-011) |

Note that full information on the approval of the study protocol must also be provided in the manuscript.
